# Chromosome-level genome of the venomous snail *Kalloconus canariensis*: a valuable model for venomics and comparative genomics

**DOI:** 10.1093/gigascience/giad075

**Published:** 2023-09-30

**Authors:** Ana Herráez-Pérez, José Ramón Pardos-Blas, Carlos M L Afonso, Manuel J Tenorio, Rafael Zardoya

**Affiliations:** Departamento de Biodiversidad y Biología Evolutiva, Museo Nacional de Ciencias Naturales (MNCN-CSIC), José Gutiérrez Abascal 2, 28006 Madrid, Spain; Departamento de Biodiversidad y Biología Evolutiva, Museo Nacional de Ciencias Naturales (MNCN-CSIC), José Gutiérrez Abascal 2, 28006 Madrid, Spain; Centre of Marine Sciences (CCMAR), Universidade do Algarve, Campus de Gambelas, 8005–139 Faro, Portugal; Departamento CMIM y Q. Inorgánica-INBIO, Facultad de Ciencias, Universidad de Cádiz, 11510 Puerto Real, Cádiz, Spain; Departamento de Biodiversidad y Biología Evolutiva, Museo Nacional de Ciencias Naturales (MNCN-CSIC), José Gutiérrez Abascal 2, 28006 Madrid, Spain

**Keywords:** Cone snails, *Kalloconus canariensis*, chromosome-level genome, comparative genomics, Omni-C

## Abstract

**Background:**

Genomes are powerful resources to understand the evolutionary mechanisms underpinning the origin and diversification of the venoms of cone snails (Conidae: Caenogastropoda) and could aid in the development of novel drugs.

**Findings:**

Here, we used PacBio continuous long reads and Omni-C data to assemble the chromosome-level genome of *Kalloconus canariensis*, a vermivorous cone endemic to the Canary Islands. The final genome size was 2.87 Gb, with a N50 of 79.75 Mb and 91% of the reads located into the 35 largest scaffolds. Up to 55.80% of the genome was annotated as repetitive regions, being class I of transposable elements (16.65%) predominant. The annotation estimated 34,287 gene models. Comparative analysis of this genome with the 2 cone snail genomes released to date (*Dendroconus betulinus* and *Lautoconus ventricosus*) revealed similar genome sizes and organization, although chromosome sizes tended to be shorter in *K. canariensis*. Phylogenetic relationships within subclass Caenogastropoda were recovered with strong statistical support. The family Conidae was recovered as a clade, with *K. canariensis* plus *L. ventricosus* sister to *D. betulinus*.

**Conclusions:**

Despite the great diversity of cone snails (>900 species) and their venoms (hundreds of peptides per species), only 2 recently reported genomes are available for the group. The high-quality chromosome-level assembly of *K. canariensis* will be a valuable reference for studying the origin and evolution of conotoxin genes as well as whole-genome duplication events during gastropod evolution.

## Background

Cones (Caenogastropoda: Conidae) are venomous marine snails that live in tropical and subtropical seas worldwide [1]. Cones produce complex venoms to capture worms, snails, and fishes, as well as to defend against predators [2, 3]. The venom is composed of short peptides termed *conotoxins*, which directly block ion channels and neuromuscular receptors in their prey and thus are the subject of intense research for novel drug development and disease treatment [4, 5]. Transcriptomics and proteomics of cone venom ducts have revealed an extraordinary diversity in the composition of venom cocktails. High-throughput long-read sequencing has opened the door to scaffold cone genomes to the chromosome level. As more cone genomes are assembled, it will be possible to perform detailed comparative genomics studies, which have the potential to unravel key details about the genetic basis of conotoxin diversity and evolution.

Here, we report *de novo* chromosome-level genome assembly of a vermivorous cone snail endemic to the Canary Islands, *Kalloconus canariensis* (NCBI:txid2750724) [6], and compare it with the only 2 other Conidae genomes released thus far, those of *Dendroconus betulinus* [7] and *Lautoconus ventricosus* [8]. The comparison of these 3 high-quality assemblies allows for the first time inferring patterns of genome evolution within this group. Their genome sizes are comparable and range from 2.87 to 3.59 Gb as well as have similar organization (into 35 pseudo-chromosomes), showing long stretches of conserved synteny.

## Materials and methods

### Sample collection

Five specimens of *K. canariensis* were collected in Playa de Porís, Tenerife, Canary Islands (Spain; GPS coordinates: 28.16447, −16.43185), in September 2020. Each individual was taken out of the shell and dissected to collect foot muscle and venom gland. The foot muscles were flash frozen in liquid nitrogen and stored at −80ºC, for subsequent high molecular weight (HMW) DNA extraction. The venom glands were preserved in RNAlater (Thermo Fisher Scientific) at −20ºC, for transcriptome assembly and genome annotation.

### Genome sequencing

The HMW DNA extraction from the foot, library preparation, long-read sequencing, contig assembly, and scaffolding were performed by Dovetail Genomics as previously described [8], except that Chicago+HiC libraries were substituted by an OmniC library, which ensures a more homogeneous digestion of chromatin and coverage (see Supplementary Methods for full details).

Long-read sequencing of HMW DNA isolated from individual TF39 (the shell was deposited as a voucher in the MNCN collection under accession number MNCN15.05/94850) was performed on PacBio Sequel II Single Molecule, Real-Time (SMRT) cells using the continuous long read (CLR) sequencing mode. A Dovetail Omni-C library to obtain proximity ligation data was generated and sequenced on an Illumina HiSeqX (see Supplementary Methods).

### Genome assembly and scaffolding

Long-read CLR sequences were *de novo* assembled using wtdgb2 v.2.5 [9] with default parameters. This software provides faster assembly speeds for large genomes with comparable contiguity and assembly accuracy and tends to have less duplicates than other assemblers [9]. The data from the *de novo* assembly and the OmniC library were used for scaffolding using HiRiSE [10]. QUAST (RRID:SCR_001228) v5.0.2 [11] and BUSCO (RRID:SCR_015008) v5.1.3. [12] were used to obtain general metrics and completeness assessment of the final genome assembly, respectively. Potential sources of DNA contamination were checked with BlobToolKit (RRID:SCR_023351) v4.1.5 [13].

### Genome annotation

RNA sequencing (RNA-seq) reads of 3 muscle (foot) and 2 venom glands of *K. canariensis* specimens were generated and pooled together for gene annotation. Repeat families were identified *de novo* and classified using RepeatModeler (RRID:SCR_015027) v2.0.1 [14]. These regions were masked with RepeatMasker (RRID:SCR_012954) v4.1.0 [15] for further genome analyses. RNA-seq data from other cone snail species downloaded from the Sequence Read Archive (SRA) database and the newly generated RNA-seq reads from *K. canariensis* were used to train 2 independent *ab initio* models for annotation using SNAP v2006-07-28 [16] and AUGUSTUS (RRID:SCR_008417) v2.5.5 [17], respectively. RNA-seq reads were mapped onto the genome using the STAR (RRID:SCR_004463) v2.7 aligner software [18], and intron hints were generated with bam2hints tools within AUGUSTUS. Gene predictions were made using both SNAP and AUGUSTUS (with intron–exon boundary hints provided from RNA-seq) with MAKER (RRID:SCR_005309) v3.01.01 [19] (see Supplementary Methods).

In addition, an alternative annotation was performed with BRAKER v2.1.6 [20–30]. The clean reads after trimmomatic of each sample (3 foot and 2 venom glands) of *K. canariensis* were mapped onto the genome assembly using STAR v2.7.10 and combined into a single sorted bam file. A set of proteins was incorporated to the analysis from the annotation of *L. ventricosus* and the Metazoa dataset from OrthoDB v.11 (RRID:SCR_011980), which are both partitions available for direct running in BRAKER2 (in -etpmode). Completeness of the annotated gene models was assessed with BUSCO v5.1.3 and metazoan OrthoDB v. 10 [12].

### Synteny analyses

**Figure 1:**
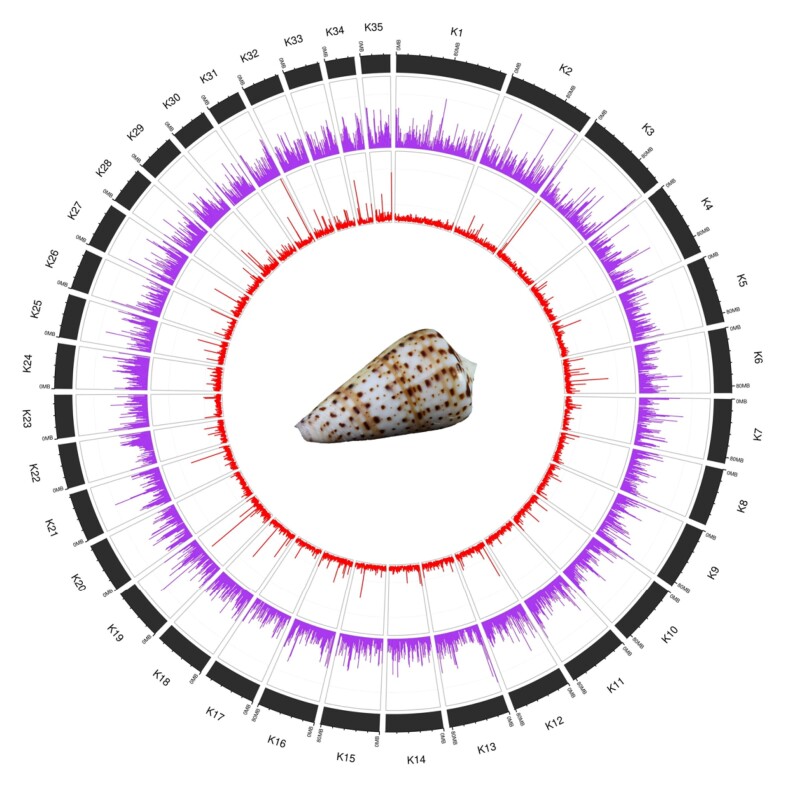
Chromosome-level genome organization of *K. canariensis*. The 35 pseudo-chromosomes are shown in black. In the inner rings, gene density is in purple (percentage of genes per Mb, normalized to the maximum number of genes, about 60) and coverage is in red (median coverage per 10 kb, normalized to sequencing depth, 130×).

All analyses were based exclusively on the 35 pseudo-chromosomes (Fig. 1, Supplementary Table S1, and Supplementary Fig. S1). Gene annotations of the *K. canariensis* and *L. ventricosus* genomes were used to compare number and length of genes, exons, and intergenic regions in both genomes (Supplementary Table S2 and Supplementary Fig. S2). The comparison with the *D. betulinus* genome was not possible, as the annotation of genes per pseudo-chromosome was not reported [7].

**Figure 2:**
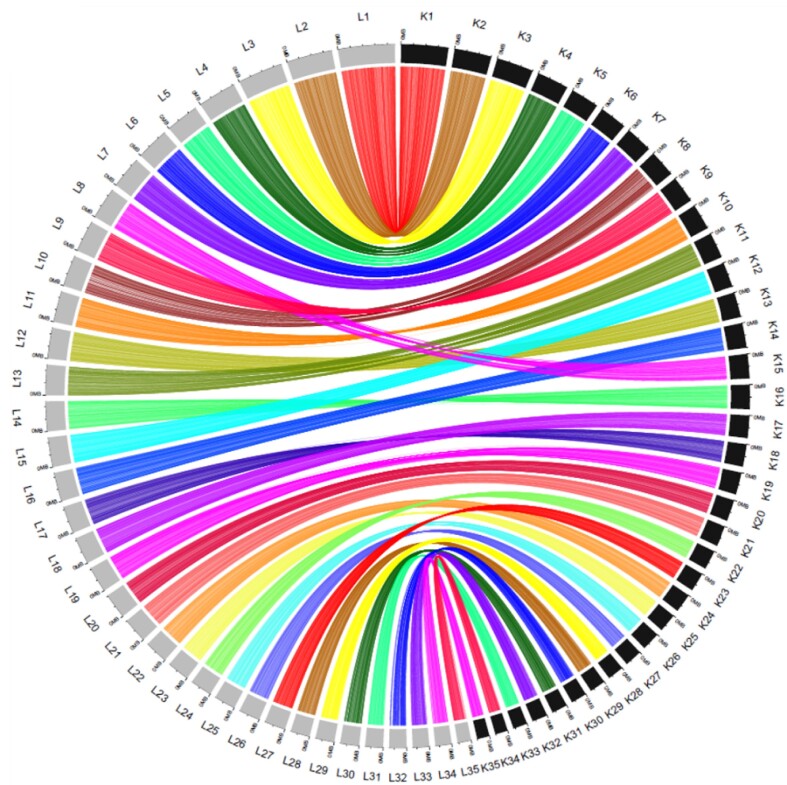
Plot of conserved synteny of the 35 pseudo-chromosomes between *K. canariensis* (right black; K1 to K35) and *L. ventricosus* (left gray; L1 to L35).

The genomes of *K. canariensis* and *L. ventricosus* were aligned using Minimap2 v2.24-r1122 (RRID:SCR_018550) [31] to infer homologue scaffolds between both species (Fig. 2 and Supplementary Fig. S3). Each scaffold of *K. canariensis* was mapped onto its homologue of *L. ventricosus* with Satsuma2 [32]. A synteny plot was generated with shinyCircos [33] for whole-genome comparison between both species (Fig. 2), and D-genies (RRID:SCR_018967) v1.3.1 [34] was used for pairwise scaffold analysis (Supplementary Fig. S3). To simplify whole-genome plots, matches <0.8 of identity and short links <1 kb were filtered out and adjacent links (within 10 Mb) were merged using bundlelinks [33].

### Phylogenomic tree reconstruction

A phylogeny of Caenogastropoda was reconstructed based on sequence data from the genomes of *K. canariensis* and *L. ventricosus* plus RNA-seq data downloaded from the SRA at NCBI from another 16 caenogastropods, plus 1 Heterobranchia (*Fiona pinnata*) and 1 Neritimorpha (*Nerita melanotragus*) as outgroup taxa (Supplementary Fig. S4 and Supplementary Table S3).

RNA-seq raw reads retrieved from SRA NCBI were assembled using Trinity (RRID:SCR_013048) v2.12.0 [35] to generate the transcriptome of each of the 18 species. For each transcriptome, the longest open reading frames were predicted and translated into protein sequences using TransDecoder (RRID:SCR_017647) v5.5.0 [36] with default settings. Protein sets were clustered and isoforms were removed with CD-HIT (RRID:SCR_007105) v4.8.1 [37] using default options and a sequence identity threshold of 0.98. Orthogroups were inferred from the protein sets using OrthoFinder (RRID:SCR_017118) v2.5.4 [38]. Single-copy orthogroups shared by at least 18 of the 20 species (occupancy of 90%) were selected using Prequal v.1.02 [39] and aligned using MAFFT (RRID:SCR_011811) v7.487 [40]. Phylogenetic informative regions were selected using BMGE v 1.12 [41]. Phylogenomic analyses based on curated amino acid alignments were performed using IQ-TREE (RRID:SCR_017254) v2.1.2 [42, 43] under maximum likelihood (ML) with 1,000 ultrafast bootstrap pseudo-replicates. The ModelFinder module of IQ-TREE [42] was used to select LG+R4 as the best-fit model, according to the Bayesian information criterion.

## Results and Discussion

### Genome assembly and scaffolding

The chromosome-level genome of *K. canariensis* was assembled and scaffolded using 371.2 Gb of PacBio CLR reads (130× coverage) and 81.1 Gb of Omni-C paired-end reads (28× coverage), respectively. PacBio long reads were *de novo* assembled into 25,961 contigs (the longest was 4.98 Mb; N50 was 646.47 kb). The HiRise scaffolding led to 18,572 scaffolds (the largest was 153.13 Mb; N50 was 79.65 Mb; see Table 1). Little signature of potential exogenous DNA contamination from bacteria was detected using the BlobToolKit viewer [13] v4.1.0 (Supplementary Fig. S5).

**Table 1:** Assembly statistics and annotation parameters of *K. canariensis* genome. All metrics are based on contigs of size ≥500 bp.

**Contig Assembly**					
	Number of reads	31,761,787 (371.2 Gb)			
	Estimated genome size	3.6 Gb			
	Total length (bp)	2,867,696,795			
	Number of contigs	25,961			
	Longest contig (bp)	4,977,278			
	N50 (bp)	646,466			
	N90 (bp)	59,506			
	GC (%)	43.84			
	BUSCO v5.1.3	(metazoa_odb10)	*n* = 954		
	Complete	93.10%	888		
	Complete single copy	87.20%	832		
	Complete duplicated	5.90%	56		
	Fragmented	4.20%	40		
	Missing	2.70%	26		
**Scaffold Assembly**					
	Total length (bp)	2,868,185,268			
	Total No. scaffolds	18,573			
	Scaffolds (≥1,000 bp)	18,495			
	Largest scaffold	153,129,599			
	N50 (bp)	79,645,777			
	N90 (bp)	40,485,963			
	GC (%)	43.84			
	BUSCO v5.1.3	(metazoa_odb10)	*n* = 954		
	Complete	93.50%	892		
	Complete single copy	87.50%	935		
	Complete duplicated	6%	57		
	Fragmented	3.80%	36		
	Missing	2.70%	26		
**Genome Annotation**					
*Repeats Masked*	Total genome masked	55.80%			
	Class I TE repeats	16.65%			
	Class II TE repeats	6.06%			
	Low-complexity repeats	0.82%			
	Simple repeats	11.19%			
		**MAKER**		**BRAKER**	
*Gene Prediction*	Total number of genes	34,250		34,287	
	Total coding region (bp)	37,756,940 (37.756 Mb)		50,351,333 (50.351 Mb)	
	Number of single-exon genes	10,549		—	
*Protein Assessment*	BUSCO v5.1.3	(metazoa_odb10)	*n* = 954	(metazoa_odb10)	*n* = 954
	Complete	81.60%	779	90.90%	867
	Complete single copy	80.90%	772	82.30%	785
	Complete duplicated	0.70%	7	8.60%	82
	Fragmented	5.10%	49	6.10%	58
	Missing	13.30%	126	3.00%	29

The final genome assembly was 2.87 Gb in length, which is smaller than those of *D. betulinus* (3.43 Gb) [7] and *L. ventricosus* (3.59 Gb) [8] but similar to the inferred genome sizes of *Kioconus tribblei* (2.76 Gb) [44] and *Textilia bullata* (2.56 Gb) [45].

The final scaffolding grouped 91.2% of the total assembled contigs into 35 scaffolds or pseudo-chromosomes, which varied in size from 153 to 40 Mb (Fig. 1 and Supplementary Fig. S1). BUSCO scores were used to assess genome completeness [12]. A total of 892 complete genes (93.5%) of the Metazoan ortholog database (odb 10) were recovered (Table 1), a value that is higher than those reported for *D. betulinus* (89.8%) [7] and *L. ventricosus* (84.9%) [8], indicating an overall higher quality of the newly reported assembly. These BUSCO metrics are also consistently higher than those inferred for *Pionoconus consors* [46] and *K. tribblei* [44] (Supplementary Fig. S6).

### Genome annotation

Repeat regions occupied 50.80% of the total genome, with class I and II of transposable elements (TEs) and simple repeats representing 16.65%, 6.06%, and 11.19%, respectively (Table 1). These proportions matched well with those of *L. ventricosus* (53.36% of the genome) [8] but were higher than those reported for the *D. betulinus* genome (38.56% of the total assembly) [7].

Gene annotation predicted a total of 34,250 genes in the MAKER genome annotation, which occupied 37.76 Mb (1.32% of the genome), whereas in the BRAKER genome annotation, the total protein coding genes predicted were to 34,287 (50.35 Mb, 1.76% of the genome). The number of genes was similar in the *L. ventricosus* genome (32,675 [8]) but considerably lower in the *D. betulinus* genome (22,698 [7]). The MAKER/BRAKER genome annotations contained 779 (81.6%)/867 (90.9%) single-copy and 7 (0.7%)/82 (8.6%) duplicated complete genes, as well as 49 (5.10%)/58 (6.1%) fragmented genes of the BUSCO Metazoan ortholog database (odb) 10, respectively (Table 1). These BUSCO metrics were similar to other molluscan genomes available at NCBI (Supplementary Table S4).

### Genome organization

Each pseudo-chromosome of *K. canariensis* had a counterpart in *L. ventricosus*, thus revealing the same genome organization and conserved macrosynteny (Fig. 2). Homologous pseudo-chromosomes in *K. canariensis* were consistently shorter than in *L. ventricosus*, with this difference particularly pronounced in pseudo-chromosomes 15 and 35 of *K. canariensis*, which were 22.45% and 23.17% smaller, respectively (Supplementary Table S1). Yet, the number of genes predicted per homologous pseudo-chromosome was slightly higher in 28 out of the 35 pseudo-chromosomes of the *K. canariensis* genome (Supplementary Table S2 and Supplementary Fig. S2).

### Gene structure

The average length of complete genes (including exons plus introns) was 41% smaller in *K. canariensis*. The genes in *K. canariensis* had fewer (10% less) but larger (8% more) exons (Supplementary Table S2 and Supplementary Fig. S2). Total intergenic regions added up to 2.48 Gb in the *K. canariensis* genome, whereas this number was 14.52% higher in the *L. ventricosus* genome. Therefore, differences in length between both genomes were concentrated in the intergenic regions, likely associated with TEs and other repetitive regions, as has been previously shown in other mollusks [47]. Further comparative analyses on repetitive landscapes between cone genomes should shed light on the nature of expansions and contractions of repetitive elements and their potential association to genome dynamics and evolution.

### Gene synteny

Pairwise comparisons between homologous pseudo-chromosomes of *K. canariensis* and *L. ventricosus* showed longer stretches of synteny in several of the largest pseudo-chromosomes (e.g., 1, 3, and 6), whereas the smallest ones tended to present more dynamic regions with lower synteny levels (e.g., 26, 30 to 35). The comparison of syntenic regions also revealed potential rearrangements within scaffolds, including large inversions, which were located in the central regions (e.g., 4, 5, 8, and 11) or at 1 terminal region (e.g., 2, 15, 16, and 29) (Supplementary Fig. S3). However, it was not possible to identify specific contigs that map across the inversion points, and thus, this result will need further confirmation as more cone genomes are generated using sequencing technologies with higher accuracy such as PacBio HiFi [48]. In any case, the high degree of synteny conservation among the 3 cone genomes offers an excellent opportunity to study the birth and death of many different gene families, as well as understand their evolutionary dynamics. In particular, they will be crucial for uncovering the main processes underpinning the generation of conotoxin diversity, which will be reported elsewhere. Furthermore, as more chromosome-level genomes of cones are reported, it will be possible also to identify those genomic regions associated with diversification and adaptation.

### Phylogenomic reconstruction

There have been different attempts to reconstruct phylogenetic relationships among caenogastropod orders based on morphology (e.g., [49]), mitogenomes (e.g., [50]), and few fragments of nuclear ribosomal RNA (rRNA) and mitochondrial genes (e.g., [51]), but they have proven to be difficult to resolve. Here, an ML phylogeny of Caenogastropoda was reconstructed based on 57 single-copy proteins and 18 species of caenogastropods representing 12 orders (Supplementary Table S3). The reconstructed phylogeny showed maximal or strong (>90%) statistical support in all but 2 nodes (Supplementary Fig. S4). The order Ampullarioidea was recovered as the first diverging branch among the caenogastropod taxa analyzed. The next order that branched off was Cerithioidea. The order Truncatelloidea was sister to 2 clades: (i) Epitonioidea sister, with low support to a well-resolved monophyletic group, including Abyssochrysoidea sister to Littorinoidea plus Naticoidea, and (ii) Stromboidea sister to Velutionoidea plus Neogastropoda (Supplementary Fig. S4). The relative phylogenetic positions here recovered are totally consistent with those based on mitogenomes [50] and a combined data set of partial rRNA and mitochondrial sequences [51] but having stronger statistical support, thus providing a robust phylogenetic framework for evolutionary studies within Caenogastropoda. For instance, it is useful for testing in combination with chromosomal-level genomes a predicted whole-genome duplication (WGD) event that predated the origin of Neogastropoda [52]. The number of chromosomes (35 in both cone species vs. 14 in the caenogastropod *Pomacea canaliculata* [53]), together with the synteny relationships between *K. canariensis* and *L. ventricosus* (this work) and between *L. ventricosus* and *P. canaliculata* [8], strongly supports such a WGD event (Supplementary Fig. S4).

Another controversy within Caenogastropoda is related to the monophyly and internal phylogenetic relationships at the superfamily level of Neogastropoda [50, 54]. Here, the monophyly of Neogastropoda is recovered with maximal support, although many important superfamilies are missing, and thus we cannot add new insights on the relative phylogenetic position of superfamilies such as Volutoidea, Tonnoidea, and Ficoidea, which are the center of a long-standing debate [50, 54]. The monophyly of the superfamily Conoidea received maximal support, and within the family Conidae, *D. betulinus* was recovered as sister to *L. ventricosus* plus *K. canariensis*, in agreement with previous phylogenies based on mitogenomes [55].

## Conclusions

Until now, only 2 genomes were available for cone snails. In this study, we provide a high-quality chromosome-level assembly of *K. canariensis*, an endemic cone snail of the Canary Islands. This new genome represents a valuable resource for comparative genomics among venomous gastropods, which are essential to understand the genetic basis of the origin and diversification of toxins and their use in novel drug development. Moreover, this annotated genome would serve as a helpful support for the assembly of other genomes within the family Conidae. Finally, because of the lack of genomic resources available for gastropods, this cone snail genome will be useful for evolutionary studies in gastropod evolution.

## Supplementary Material

giad075_GIGA-D-23-00046_Original_Submission

giad075_GIGA-D-23-00046_Revision_1

giad075_Response_to_Reviewer_Comments_Original_Submission

giad075_Reviewer_1_Report_Original_Submission Richard Lewis -- 3/21/2023 Reviewed

giad075_Reviewer_1_Report_Revision_1 Richard Lewis -- 7/10/2023 Reviewed

giad075_Reviewer_2_Report_Original_Submission Luis Javier Chueca, PhD -- 3/21/2023 Reviewed

giad075_Supplemental_File

## Data Availability

Raw reads are available at SRA-NCBI under BioProject PRJNA843968 with accession numbers SRR19919783 and SRR20083570 for PacBio and OmniC reads, respectively. The Whole Genome Shotgun project has been deposited at GenBank under accession number JAMYXO000000000. All supporting data and materials are available in the *GigaScience* GigaDB [56].

## Additional Files

**Supplementary Methods**. Whole-genome assembly, scaffolding, and annotation of *Kalloconus canariensis*.

**Supplementary Fig. S1**. Link density histogram (35 pseudo-chromosomes).

**Supplementary Fig. S2**. Differences in gene and intergenic region lengths between *L. ventricosus* (left blue, Lven) and *K. canariensis* (right orange, Kcan).

**Supplementary Fig. S3**. Synteny maps between *L. ventricosus* (L1 to L35) and *K. canariensis* (K1 to K35) pseudo-chromosomes.

**Supplementary Fig. S4**. Maximum likelihood phylogenomic relationships of Caenogastropoda (green branches) based on 57 single-copy proteins of 20 gastropod species. Neritimorpha and Heterobranchia were used as outgroups. Neogastropoda and Conidae are depicted with slashed purple and red boxes, respectively. Numbers at nodes are boostrap support values. The circle represents an ancestral WGD event. The reconstructed tree was visualized with FigTree v1.4.4 (RRID:SCR_008515).

**Supplementary Fig. S5**. DNA sources in the *Kalloconus canariensis* genome.

**Supplementary Fig. S6**. BUSCO assessment of the available assembled genomes of cone snails. BUSCO v.5.1.3 and Metazoan Ortholog Database (odb10, *n* = 954) were used.

**Supplementary Table S1**. Sizes of homologous pseudo-chromosomes of *K. canariensis* and *L. ventricosus*.

**Supplementary Table S2**. Comparison of number and length of genes and intergenic regions of homologous pseudo-chromosomes of *K. canariensis* and *L. ventricosus*.

**Supplementary Table S3**. Taxonomy of gastropods and source of sequences included in phylogenomic analyses.

**Supplementary Table S4**. Genome assembly and BUSCO metrics of genome annotations of molluscs available at NCBI.

## Abbreviations

BMGE: Block Mapping and Gathering Entropy; BLAST: Basic Local Alignment Search Tool; BUSCO: Benchmarking Universal Single-Copy Orthologs; CLR: continuous long read; Gb: gigabase pairs; HiFi: High-Fidelity; HMW: high molecular weight; kb: kilobase pairs; MAFFT: Multiple Alignment using Fast Fourier Transform; Mb: megabase pairs; ML: maximum likelihood; NCBI: National Center for Biotechnology Information; PacBio: Pacific BioSciences; QUAST: QUality ASsessment Tool; RNA-seq: RNA sequencing; rRNA: ribosomal RNA; SMRT: Single Molecule Real-Time; SNAP: Semi-hidden Markov Model–Based Nucleic Acid Parser; SRA: Sequencing Read Archive; STAR: Spliced Transcripts Alignment to a Reference; TE: transposable element; WGD: whole-genome duplication.
