# Supplementary material for: Chromosome-level genome of the venomous snail Kalloconus canariensis: a valuable model for venomics and comparative genomics
Source: Gigascience. 2023 Sep 30;12:giad075. doi: 10.1093/gigascience/giad075 (PMC10541794; doi:10.1093/gigascience/giad075)
Supplement: giad075_GIGA-D-23-00046_Original_Submission [file giad075_giga-d-23-00046_original_submission.pdf]

GigaScience

Chromosome-Level Genome of the Venomous Snail Kalloconus canariensis: A Valuable Model for Venomics and Comparative Genomics

--Manuscript Draft--

Manuscript Number:	GIGA-D-23-00046
Full Title:	Chromosome-Level Genome of the Venomous Snail Kalloconus canariensis: A Valuable Model for Venomics and Comparative Genomics
Article Type:	Data Note
Funding Information:	Ministerio de Ciencia e Innovación (PID2019-103947GB-C22) — Prof. Rafael Zardoya<br>Ministerio de Ciencia e Innovación (BES2017–081195) — Dr. José Ramón Pardos-Blas<br>Ministerio de Ciencia e Innovación (PRE2020-095119) — Ms Ana Herráez-Pérez
Abstract:	Background: Genomes are powerful resources to understand the evolutionary mechanisms underpinning the origin and diversification of the venoms of cone snails (Conidae: Caenogastropoda), and could aid in the development of novel drugs. Findings: Here, we used PacBio CLR reads and Omni-C data to assemble the chromosome-level genome of Kalloconus canariensis, a vermivorous cone endemic to the Canary Islands. The final genome size was 2.87 Gb, with a N50 of 79.75 Mb and 91 % of the reads located into the 35 largest scaffolds. Up to 49.55 % of the genome was annotated as repetitive regions, being Class I of transposable elements (18.56%) predominant. The annotation estimated 34,241 gene models. Comparative analysis of this genome with the two cone snail genomes released to date (Dendroconus betulinus and Lautoconus ventricosus) revealed similar genome sizes and organization, although chromosome sizes tended to be shorter in K. canariensis. Phylogenetic relationships within subclass Caenogastropoda were recovered with strong statistical support. The family Conidae was recovered as a clade, with K. canariensis plus L. ventricosus sister to D. betulinus. Conclusions: Despite the great diversity of cone snails (>900 species) and their venoms (hundreds of peptides per species), only two recently reported genomes are available for the group. The high-quality chromosome-level assembly of K. canariensis will be a valuable reference for studying the origin and evolution of conotoxin genes as well as whole genome duplication (WGD) events during gastropod evolution.
Corresponding Author:	Rafael Zardoya, PhD<br>Museo Nacional de Ciencias Naturales-CSIC<br>Madrid, Madrid SPAIN
Corresponding Author Secondary Information:	
Corresponding Author's Institution:	Museo Nacional de Ciencias Naturales-CSIC
Corresponding Author's Secondary Institution:	
First Author:	Ana Herráez-Pérez
First Author Secondary Information:	
Order of Authors:	Ana Herráez-Pérez<br>José Ramón Pardos-Blas<br>Carlos Manuel Lourenço Afonso<br>Manuel J. Tenorio<br>Rafael Zardoya, PhD
Order of Authors Secondary Information:	
Additional Information:	

Powered by Editorial Manager® and ProduXion Manager® from Aries Systems Corporation

Question	Response
Are you submitting this manuscript to a special series or article collection?	No
**Experimental design and statistics**<br><br>Full details of the experimental design and statistical methods used should be given in the Methods section, as detailed in our Minimum Standards Reporting Checklist. Information essential to interpreting the data presented should be made available in the figure legends.<br><br>Have you included all the information requested in your manuscript?	Yes
**Resources**<br><br>A description of all resources used, including antibodies, cell lines, animals and software tools, with enough information to allow them to be uniquely identified, should be included in the Methods section. Authors are strongly encouraged to cite Research Resource Identifiers (RRIDs) for antibodies, model organisms and tools, where possible.<br><br>Have you included the information requested as detailed in our Minimum Standards Reporting Checklist?	Yes
**Availability of data and materials**<br><br>All datasets and code on which the conclusions of the paper rely must be either included in your submission or deposited in publicly available repositories (where available and ethically appropriate), referencing such data using a unique identifier in the references and in the "Availability of Data and Materials" section of your manuscript.	Yes

Have you have met the above requirement as detailed in our Minimum Standards Reporting Checklist?	

Click here to access/download;Manuscript;K.canariensis data report.pdf

To be submitted to:
GigaScience
Data Note
Version: 20/02/2023

Chromosome-Level Genome of the Venomous Snail *Kalloconus canariensis*: A Valuable Model for Venomics and Comparative Genomics

Ana Herráez-Pérez[1], José Ramón Pardos-Blas[1], Carlos M.L. Afonso[2], Manuel J. Tenorio[3] and Rafael Zardoya[1]*

[1] Departamento de Biodiversidad y Biología Evolutiva, Museo Nacional de Ciencias Naturales (MNCN-CSIC), José Gutiérrez Abascal 2, 28006 Madrid, Spain.

[2] Centre of Marine Sciences (CCMAR), Universidade do Algarve, Campus de Gambelas, 8005–139 Faro, Portugal.

[3] Departamento CMIM y Q. Inorgánica-INBIO, Facultad de Ciencias, Universidad de Cádiz, 11510 Puerto Real, Cádiz, Spain.

[*] **Correspondence address.** Rafael Zardoya, Departamento de Biodiversidad y Biología Evolutiva, Museo Nacional de Ciencias Naturales (MNCN-CSIC),

José Gutiérrez Abascal 2, 28006 Madrid, Spain. E-mail: rafaz@mncn.csic.es

http://orcid.org/0000-0001-6212-9502

Abstract

Background: Genomes are powerful resources to understand the evolutionary mechanisms underpinning the origin and diversification of the venoms of cone snails (Conidae: Caenogastropoda), and could aid in the development of novel drugs. **Findings:** Here, we used PacBio CLR reads and Omni-C data to assemble the chromosome-level genome of *Kalloconus canariensis*, a vermivorous cone endemic to the Canary Islands. The final genome size was 2.87 Gb, with a N50 of 79.75 Mb and 91 % of the reads located into the 35 largest scaffolds. Up to 49.55 % of the genome was annotated as repetitive regions, being Class I of transposable elements (18.56%) predominant. The annotation estimated 34,241 gene models. Comparative analysis of this genome with the two cone snail genomes released to date (*Dendroconus betulinus* and *Lautoconus ventricosus*) revealed similar genome sizes and organization, although chromosome sizes tended to be shorter in *K. canariensis*. Phylogenetic relationships within subclass Caenogastropoda were recovered with strong statistical support. The family Conidae was recovered as a clade, with *K. canariensis* plus *L. ventricosus* sister to *D. betulinus*. **Conclusions:** Despite the great diversity of cone snails (>900 species) and their venoms (hundreds of peptides per species), only two recently reported genomes are available for the group. The high-quality chromosome-level assembly of *K. canariensis* will be a valuable reference for studying the origin and evolution of conotoxin genes as well as whole genome duplication (WGD) events during gastropod evolution.

Keywords: Cone snails, *Kalloconus canariensis*, chromosome-level genome, comparative genomics, Omni-C.

Background

Cones (Caenogastropoda: Conidae) are venomous marine snails that live in tropical and subtropical seas worldwide [1]. Cones produce complex venoms to capture worms, snails, and fishes, as well as to defend against predators [2, 3]. The venom is composed of short peptides termed conotoxins, which directly block ion channels and neuromuscular receptors in their preys and thus, are the subject of intense research for novel drug development and disease treatment [4, 5]. Transcriptomics and proteomics of cone venom ducts have revealed an extraordinary diversity in the composition of venom cocktails. High-throughput long read sequencing have opened the door to scaffold cone genomes to the chromosome level. As more cone genomes are assembled, it will be possible to perform detailed comparative genomics studies, which have the potential to unravel key details about the genetic basis of conotoxin diversity and evolution.

Here, we report *de novo* chromosome-level genome assembly of a vermivorous cone snail endemic to the Canary Islands, *Kalloconus canariensis* [6], and compare it with the only two other Conidae genomes released thus far, those of *Dendroconus betulinus* [7] and *Lautoconus ventricosus* [8]. The comparison of these three high-quality assemblies allows for the first time inferring patterns of genome evolution within this group. They are similar in size (around 3 Gb) and organization (into 35 pseudo-chromosomes), showing long stretches of conserved synteny.

Materials and Methods

Sample collection

Five specimens of *K. canariensis* were collected in Tenerife, Canary Islands (Spain) in September 2020. Each individual was taken out of the shell and dissected to collect foot

muscle and venom gland. The foot muscles were flash frozen in liquid nitrogen and stored at -80ºC, for subsequent high molecular weight (HMW) DNA extraction. and the venom glands were preserved in RNAlater (Thermo Fisher Scientific, Waltham, MA, USA) at -20ºC, for transcriptome assembly and genome annotation.

Genome sequencing

The HMW DNA extraction, library preparation, long read sequencing, contig assembly and scaffolding were performed by Dovetail Genomics (Scotts Valley, CA, USA) as previously described [8], except that Chicago+HiC libraries were substituted by an OmniC library, which ensures a more homogeneous digestion of chromatin and coverage (see Supplementary methods, for full details).

Long read sequencing of HMW DNA isolated from individual TF39 (the shell was deposited as voucher in the MNCN collection under accession number (MNCN15.05/94850) was performed on PacBio Sequel II Single Molecule, Real-Time (SMRT) cells using the Continuous Long Read (CLR) sequencing mode. A Dovetail Omni-C library to obtain proximity ligation data was generated and was sequenced on an Illumina HiSeqX (see Supplementary methods).

Genome assembly and scaffolding

Long read CLR sequences were *de novo* assembled using wtdgb2 v.2.5 [9]. The data from the *de novo* assembly and the OmniC library were used for scaffolding using HiRiSE™ [10]. QUAST (QUAST, RRID:SCR_001228) v5.0.2 [11] and BUSCO (BUSCO, RRID:SCR_015008) v5.1.3. [12] were used to obtain general metrics and completeness assessment of the final genome assembly, respectively.

Genome annotation

Foot muscles and venom glands were used to generate the corresponding transcriptomes for genome annotation. The complete protocol is described in Supplementary methods. Repeat families were identified *de novo* and classified using RepeatModeler (RepeatModeler, RRID:SCR_015027) v2.0.1. [13]. These regions were masked with RepeatMasker (RepeatMasker, RRID:SCR_012954) v4.1.0 [14] for further genome analyses. Coding sequences from other cone snail species and the transcriptomes generated in this work were used to train two independent *ab initio* models for annotation using SNAP v2006-07-28 [15] and AUGUSTUS (Augustus, RRID:SCR_008417) v2.5.5 [16], respectively. RNAseq reads were mapped onto the genome using the STAR (STAR, RRID:SCR_004463) v2.7 aligner software [17] and intron hints were generated with bam2hints tools within AUGUSTUS. Gene predictions were made using both SNAP and AUGUSTUS (with intron-exon boundary hints provided from RNA-Seq) with MAKER (MAKER, RRID:SCR_005309) v3.01.01 [18] (see Supplementary methods). Completeness of the annotated gene models was assessed with BUSCO v5.1.3. [12], and an additional TBLASTN similarity search of the genome assembly against the translated missing proteins from BUSCO results was performed (>70% of sequence identity).

Synteny analyses

All statistics were based exclusively on the 35 pseudo-chromosomes (Fig. 1, Supplementary Table S1 and Fig. S1). Gene annotations of the *K. canariensis* and *L. ventricosus* genomes were used to compare number and length of genes, exons and intergenic regions in both genomes (Supplementary Table S2 and Fig. S2). The

comparison with the *D. betulinus* genome was not possible, as the annotation of genes per pseudo-chromosome was not reported [7].

The genomes of *K. canariensis* and *L. ventricosus* were aligned using Minimap2 v2.24-r1122 (Minimap2, RRID:SCR_018550) [19] to infer homologue scaffolds between both species (Fig 2 and Supplementary Fig. S3). Each scaffold of *K. canariensis* was mapped onto its homologue of *L. ventricosus* with Satsuma2 [20]. A synteny plot was generated with shinyCircos [21] for whole genome comparison between both species (Fig. 2), and D-genies (D-genies, RRID:SCR_018967) v1.3.1 [22] was used for pairwise scaffold analysis (Supplementary Fig. S3). To simplify whole genome plots, matches <0.8 of identity and short links <1kb were filtered out and adjacent links (within 10 Mb) were merged using bundlelinks [21].

Phylogenomic Tree Reconstruction

A phylogeny of Caenogastropoda was reconstructed based on sequence data from the genomes of *K. canariensis* and *L. ventricosus* plus RNA-seq data downloaded from the Sequence Read Archive (SRA) at NCBI (https://www.ncbi.nlm.nih.gov/sra) from another 16 caenogastropods, plus one Heterobranchia (*Fiona pinnata*) and one Neritimorpha (*Nerita melanotragus*) as outgroup taxa (Supplementary Fig. S4 and Table S3).

Transcriptomes were assembled using SPAdes (Spades, RRID:SCR_000131) v3.15.4 [23]. For each transcriptome, the longest ORFs were predicted and translated into protein sequences using TransDecoder (TransDecoder, RRID:SCR_017647) v5.5.0 [24] with default settings. Protein sets were clustered and isoforms were removed with CD-HIT (CD-HIT, RRID:SCR_007105) v4.8.1 [25] using default options and a sequence identity threshold of 0.98. Orthogroups were inferred from the protein sets using

Orthofinder (OrthoFinder, RRID:SCR_017118) v2.5.4 [26]. Single-copy orthogroups shared by at least 18 of the 20 species (occupancy of 90%) were selected using Prequal v.1.02 [27] and aligned using MAFFT (MAFFT, RRID:SCR_011811) v7.487 [28]. Phylogenetic informative regions were selected using BMGE v 1.12 [29]. Phylogenomic analyses based on curated amino acid alignments were performed using IQ-TREE (IQ-TREE, RRID:SCR_017254) v2.1.2 [30, 31] under maximum likelihood (ML) with 1000 ultrafast bootstrap pseudo-replicates. The selected best-fit model was LG+R4 according to the Bayesian Information Criterion (BIC).

Results and Discussion

Genome assembly, and scaffolding

The chromosome–level genome of *K. canariensis* was assembled and scaffolded using 371.2 Gb of PacBio CLR reads (130x coverage) and 81.1 Gb of Omni-C paired-end reads (28x coverage), respectively. PacBio long reads were *de novo* assembled into 25,961 contigs (the longest was 4.98 Mb; N50 was 646.47 kb). The HiRise scaffolding led to 18,572 scaffolds (the largest was 153.13 Mb; N50 was 79.65 Mb; see Table 1).

Table 1: Assembly statistics and annotation parameters of *K. canariensis* genome. All metrics are based on contigs of size ≥ 500 bp.

The final genome assembly was 2.87 Gb in length, which is smaller than those of *D. betulinus* (3.43 Gb) [7] and *L. ventricosus* (3.59 Gb) [8], but similar to the inferred genome sizes of *Kioconus tribblei* (2.76 Gb) [32] and *Textilia bullata* (2.56 Gb) [33].

The final scaffolding grouped 91.2% of the total assembled contigs into 35 scaffolds or pseudo-chromosomes, which varied in size from 153 to 40 Mb (Fig. 1 and

Supplementary Fig. S1). BUSCO scores were used to assess genome completeness [12]. A total of 892 complete genes (93.5%) of the Metazoan ortholog database (odb 10) were recovered (Table 1), a value that is higher than those reported for *D. betulinus* (89.8%) [7] and *L. ventricosus* (84.9%) [8], indicating an overall higher quality of the newly reported assembly. These BUSCO metrics are also consistently higher than those inferred for the early assembled genomes of *Pionoconus consors* [34] and *Kioconus tribblei* [32] (Supplementary Fig. S5).

Figure 1: Chromosome-level genome organization of *K. canariensis*. The 35 pseudo-chromosomes are shown in black. In the inner rings, gene density in purple (percentage of genes per Mb, normalized to the maximum number of genes, about 60) and coverage in red (median coverage per 10 kb, normalized to sequencing depth, 130x).

Genome annotation

Repeat regions occupied 49.55% of the total genome, with Class I and II of transposable elements (TEs) and simple repeats representing 18.56, 10.51, and 7.58%, respectively (Table 1). These proportions matched well with those of *L. ventricosus* (53.36% of the genome) [8] but were higher than those reported for the *D. betulinus* genome (38.56% of the total assembly) [7].

Gene annotations predicted a total of 34,241 gene models, which occupied 37.76 Mb (1.32% of the genome). The number of genes was similar in the *L. ventricosus* genome (32,675 [8]) but considerably lower in the *D. betulinus* genome (22,698 [7]). The genome annotation contained 779 single copy (81.6 %) and 7 duplicated (0.7 %) complete genes, as well as 49 fragmented genes (5.10 %) of the BUSCO Metazoan ortholog database (odb) 10 (table 1). The remaining 126 (13.3%) missing BUSCOs

were search by aligning the genome assembly against translated proteins to recover 1 complete protein and 28 fragmented, reaching up to 89.9 % of the total BUSCO gene models (complete plus fragmented genes).

These BUSCO metrics for genome annotations were similar to those of other molluscan genomes available at NCBI (Supplementary Fig.S6 and Table S4). Differences in BUSCO metrics may be due to low conservation of orthologues between *K. canariensis* and the metazoan odb reference set, compared to other molluscs, or may be explained by a certain degree of fragmentation in the gene models.

Genome organization

Each pseudo-chromosome of *K. canariensis* had a counterpart in *L. ventricosus*, thus revealing the same genome organization and conserved macrosynteny (Fig. 2). Homologous pseudo-chromosomes in *K. canariensis* were consistently shorter than in *L. ventricosus,* being this difference particularly pronounced in pseudo-chromosomes 15 and 35 of *K. canariensis,* which were *22.45%* and *23.17%* smaller, respectively (Supplementary Table S1). Yet, the number of genes predicted per homologous pseudo-chromosome was slightly higher in 28 out of the 35 pseudo-chromosomes of the *K. canariensis* genome (Supplementary Table S2 and Fig. S2).

Figure 2: Plot of conserved synteny of the 35 pseudo-chromosomes between *K. canariensis* (right black; K1 to K35) and *L. ventricosus* (left grey; L1 to L35).

Gene structure

The average length of complete genes (including exons plus introns) was 41% smaller in *K. canariensis.* The genes in *K. canariensis* had fewer (10% less) but larger (8%

more) exons (Supplementary Table S2 and suppl. Fig. S2). Total intergenic regions added up to 2.48 Gb in the *K. canariensis* genome, whereas this number was 14.52% higher in the *L. ventricosus* genome. Therefore, differences in length between both genomes were concentrated in the intergenic regions, likely associated to transposable elements and other repetitive regions, as has been previously shown in other mollusks [35]. Further comparative analyses on repetitive landscapes between cone genomes should shed light on the nature of expansions and contractions of repetitive elements and their potential association to genome dynamics and evolution.

Gene synteny

Pairwise comparisons between homologous pseudo-chromosomes of *K. canariensis* and *L. ventricosus* showed longer stretches of synteny in several of the largest pseudo-chromosomes (e.g., 1, 3, and 6), whereas the smallest ones tend to present more dynamic regions with lower synteny levels (e.g., 26, 30 to 35). The comparison of syntenic regions also revealed potential rearrangements within scaffolds, including large inversions, which were located in the central (e.g., 4, 5, 8, and 11) or at one terminal (e.g., 2, 15, 16, and 29) regions (Supplementary Fig. S3). However, it was not possible to identify specific contigs that map across the inversion points and thus, this result will need further confirmation as more cone genomes are generated using sequencing technologies with higher accuracy such as PacBio HiFi [36]. In any case, the high degree of synteny conservation among the three cone genomes offers an excellent opportunity to study the birth and death of many different gene families, and understand their evolutionary dynamics. In particular, they will be crucial for uncovering the main processes underpinning the generation of conotoxin diversity, which will be reported elsewhere. Furthermore, as more chromosome-level genomes of cones are reported, it

will be possible also to identify those genomic regions associated with diversification and adaptation.

Phylogenomic reconstruction

There have been different attempts to reconstruct phylogenetic relationships among caenogastropod orders based on morphology (e.g., [37]), mitogenomes (e.g., [38]), and few fragments of nuclear rRNA and mitochondrial genes (e.g., [39]), but they have proven to be difficult to resolve. Here, a maximum likelihood (ML) phylogeny of Caeogastropoda was reconstructed based on 57 single copy proteins and 18 species of caenogastropods representing 12 orders (Supplementary Table S3). The reconstructed phylogeny showed maximal or strong (>90%) statistical support in all but two nodes (Supplementary Fig. S4). The order Ampullarioidea was recovered as the first diverging branch among the caenogastropod taxa analyzed. The next order that branched off was Cerithioidea. The order Truncatelloidea was sister to two clades: (1) Epitonioidea sister with low support to a well-resolved monophyletic group including Abyssochrysoidea sister to Littorinoidea plus Naticoidea; (2) Stromboidea sister to Velutionoidea plus Neogastropoda (Supplementary Fig. S4). The relative phylogenetic positions here recovered are totally consistent with those based on mitogenomes [38] and a combined data set of partial rRNA and mitochondrial sequences [39] but having stronger statistical support, and thus providing a robust phylogenetic framework for evolutionary studies within Caenogastropoda. For instance, it is useful for testing in combination with chromosomal-level genomes a predicted WGD event that predated the origin of Neogastropoda [40]. The number of chromosomes (35 in both cone species *versus* 14 in the caenogastrod *Pomacea canaliculata* [41]) together with the synteny relationships

between *K. canariensis* and *L. ventricosus* (this work) and between *L. ventricosus* and *P. canaliculata* [8] strongly support such WGD event (Supplementary Fig. S4).

Another controversy within Caenogastropoda is related with the monophyly and internal phylogenetic relationships at the superfamily level of Neogastropoda [38, 42]. Here, the monophyly of Neogastropoda is recovered with maximal support, although many important superfamilies are missing, and thus we cannot add new insights on the relative phylogenetic position of superfamilies such as Volutoidea, Tonnoidea and Ficoidea, which are the center of a long-standing debate [38, 42]. The monophyly of the superfamily Conoidea received maximal support and within the family Conidae, *D. betulinus* was recovered as sister to *L. ventricosus* plus *K. canariensis*, in agreement with previous phylogenies based on mitogenomes [43].

Conclusions

Until now only two reference genomes were available for cone snails. In this study, we provide a high-quality chromosome-level assembly of *Kalloconus canariensis*, an endemic cone snail of the Canary Islands. This new genome represents a valuable resource for comparative genomics among venomous gastropods, which are essential to understand the genetic basis of the origin and diversification of toxins and its use in novel drug development. Moreover, this annotated genome would serve as a helpful support for the assembly of other genomes within the family Conidae. Finally, because of the lack of genomic resources available for gastropods, this cone snail genome will be useful for evolutionary studies in gastropod evolution.

Additional Files

Supplementary Methods: Whole-genome assembly, scaffolding, and annotation of *Kalloconus canariensis.*

Supplementary Figure S1: Link density histogram (35 pseudochromosomes).

Supplementary Figure S2: Differences in gene and intergenic regions lengths between *L. ventricosus* (left blue, Lven) and *K. canariensis* (right orange, Kcan).

Supplementary Figure S3: Synteny maps between *L. ventricosus* (L1 to L35) and *K. canariensis* (K1 to K35) pseudo-chromosomes.

Supplementary Figure S4: Maximum likelihood phylogenomic relationships of Caenogastropoda (green branches) based on 57 single copy proteins of 20 gastropod species. Neritimorpha and Heterobranchia were used as outgroups. Neogastropoda and Conidae are depicted with slashed purple and red boxes, respectively. Numbers at nodes are boostrap support values. The circle represents an ancestral Whole Genome Duplication (WGD) event [40]. The reconstructed tree was visualized with FigTree v1.4.4 (http://tree.bio.ed.ac.uk/software/figtree/).

Supplementary Figure S5: BUSCO assessment of the available assembled genomes of Cone Snails. BUSCO v.5.1.3. and Metazoan Ortholog Database (odb10, n=954) were used.

Supplementary Figure S6: BUSCO assessment of the genome annotations of Molluscs available at NCBI (https://www.ncbi.nlm.nih.gov/sra). BUSCO v.5.1.3. and Metazoan Ortholog Database (odb10, n=954) were used.

Supplementary Table S1: Sizes of homologous pseudo-chromosomes of *K. canariensis* and *L. ventricosus*.

Supplementary Table S2: Comparison of number and length of genes and intergenics regions of homologous pseudo-chromosomes of *K. canariensis* and *L. ventricosus*.

Supplementary Table S3: Taxonomy of gastropods and source of sequences included in phylogenomic analyses.

Supplementary Table S4: Genome assembly and BUSCO metrics of genome annotations of molluscs available at NCBI (https://www.ncbi.nlm.nih.gov/sra).

Data Availability

Raw reads are available at SRA-NCBI under BioProject PRJNA843968 with accession numbers SRR19919783 and SRR20083570 for PacBio and OmniC reads, respectively. The Whole Genome Shotgun project has been deposited at GenBank under accession number JAMYXO000000000. All supporting data and materials are available in the *GigaScience* GigaDB.

Abbreviations

BMGE: Block Mapping and Gathering Entropy; BIC: Bayesian Information Criterion; BLAST: Basic Local Alignment Search Tool; BUSCO: Benchmarking Universal Single-Copy Orthologs; CLR: continuous long read; Gb: gigabase pairs; HiFi: High-Fidelity; HMMs: Hidden Markov Models; HMW: high-molecular weight; kb: kilobase pairs; MAFFT: Multiple Alignment using Fast Fourier Transform; Mb: megabase pairs; ML: maximum likelihood; NCBI: National Center for Biotechnology Information; ORF: open reading frame; PacBio: Pacific BioSciences; QUAST: QUality ASsessment Tool; RNA-seq: RNA sequencing; rRNA: ribosomal RNA; SMRT: Single Molecule Real-Time; SNAP: Semi-HMM-based Nucleic Acid Parser; SPAdes: St. Petersburg genome assembler; SRA: Sequencing Read Archive; STAR: Spliced Transcripts Alignment to a Reference; TE: transposable element; WGD: whole-genome duplication.

Competing Interests

The authors declare that they have no competing interests

Funding

This work was supported by the Spanish Ministry of Science and Innovation [PID2019-103947GB-C22 to R.Z.; BES2017–081195 to J.R.P.-B. and PRE2020-095119 to A.H.-P].

Authors' Contributions

R.Z. conceived the study and designed the analyses. M.J.T. and C.M.L.A. obtained the individuals, performed sample dissections, and provided information on cone snail biology. A.H.-P. and J.R.P.-B performed the bioinformatics analyses. R.Z. wrote the initial draft, and all authors read, revised, and approved the manuscript final version.

Acknowledgments

We thank Juan E. Uribe for help during field sampling and with phylogenomic reconstruction. We are grateful to Vadim A. Pisarenco and Julio Rozas for help with synteny analysis and providing access to the Hercules computer cluster, respectively.

References

1. Tucker JM, Tenorio MJ. Illustrated catalog of the living cone shells. MdM Publishing: Wellington; 2013.
2. Dutertre S, Jin A-H, Vetter I, et al. Evolution of separate predation- and defence-evoked venoms in carnivorous cone snails. *Nat commun*. 2014;**5**:3521.
3. Puillandre N, Bouchet P, Duda TF, et al. Molecular phylogeny and evolution of the cone snails (Gastropoda, Conoidea). *Mol Phylogenet Evol*. 2014:**78**:290-303.
4. Lewis RJ, Dutertre S, Vetter I, et al. *Conus* venom peptide pharmacology. *Pharmacol Rev.* 2012;**64**:259–98.
5. Robinson SD, Norton RS. Conotoxin gene superfamilies. *Mar Drugs*. 2014;**12**:6058-101.
6. Tenorio MJ, Abalde S, Pardos-Blas JR, et al. Taxonomic revision of West African cone snails (Gastropoda: Conidae) based upon mitogenomic studies: implications for conservation. *Eur J Taxon.* 2020;**663**:1-89.
7. Peng C, Huang Y, Bian C, et al. The first *Conus* genome assembly reveals a primary genetic central dogma of conopeptides in *C. betulinus. Cell Discov*. 2021;**7**:11.
8. Pardos-Blas JR et al. The genome of the venomous snail *Lautoconus ventricosus* sheds light on the origin of conotoxin diversity. *GigaScience*. 2021;**10**, doi: 10.1093/gigascience/giab037.
9. Ruan J, Li H. Fast and accurate long-read assembly with wtdbg2. *Nat Methods*. 2020;**17**:155–8.
10. Putnam NH, O'Connell BL, Stites JC, et al. Chromosome scale shotgun assembly using an in vitro method for long-range linkage. *Genome Res*. 2016;**26**:342–50.

11. Gurevich A, Saveliev V, Vyahhi N, et al. QUAST: quality assessment tool for genome assemblies. *Bioinformatics*. 2013;**29**(8):1072–5.

12. Manni M, Berkeley MR, Seppey M, et al. BUSCO update: novel and streamlined workflows along with broader and deeper phylogenetic coverage for scoring of eukaryotic, prokaryotic, and viral genomes. *Mol Biol Evol*. 2021;**38**(10): 4647-54.

13. Flynn JM, Hubley R, Goubert C, et al. RepeatModeler2 for automated genomic discovery of transposable element families. *Proc Natl Acad Sci USA*. 2020;**117**(17):9451–7.

14. Smit AFA, Hubley R, Green P. RepeatMasker Open-4.1.0. Available from: http://www.repeatmasker.org/. 2019-2020.

15. Korf I. Gene finding in novel Genomes. *BMC Bioinformatics*. 2004;**5**:59

16. Stanke M, Steinkamp R, Waack S, et al. AUGUSTUS: a web server for gene finding in eukaryotes. *Nucleic Acids Res*. 2005;**32**:W309–12.

17. Dobin A, Davis CA, Schlesinger F, et al. STAR: ultrafast universal RNA-seq aligner. *Bioinformatics*. 2013;**29**:15–21.

18. Holt C, Yandell M. MAKER2: an annotation pipeline and genome-database management tool for second-generation genome projects. *BMC Bioinformatics.* 2011;**12**:491.

19. Li H. Minimap2: pairwise alignment for nucleotide sequences. *Bioinformatics*. 2018;**34**:3094-100.

20. Grabherr MG, Russell P, Meyer M, et al. Genome-wide synteny through highly sensitive sequence alignment: Satsuma. *Bioinformatics*. 2010;**26**(9):1145-51.

21. Yu Y, Ouyang Y, Yao W. shinyCircos: an R/Shiny application for interactive creation of Circos plot. *Bioinformatics*. 2018;**34**:1229–31.

22. Cabanettes F, Klopp C. D-GENIES: dot plot large genomes in an interactive, efficient and simple way. *PeerJ.* 2018;**6**:e4958

23. Bushmanova E, Antipov D, Lapidus A, et al. rnaSPAdes: a de novo transcriptome assembler and its application to RNA-Seq data, *GigaScience*. 2019;**8**, doi: 10.1093/gigascience/giz100.

24. Haas BJ, Papanicolaou A, Yassour M, et al. De novo transcript sequence reconstruction from RNA-seq using the Trinity platform for reference generation and analysis. *Nat Protoc.* 2013;**8**(8): 1494-512.

25. Fu L, Niu B, Zhu Z, et al. CD-HIT: accelerated for clustering the next generation sequencing data. *Bioinformatics*. 2012;**28**(23):3150-2.

26. Emms DM, Kelly S. OrthoFinder: phylogenetic orthology inference for comparative genomics. *Genome Biol.* 2019;**20**:238.

27. Whelan S, Irisarri I, Burki F. PREQUAL: detecting non-homologous characters in sets of unaligned homologous sequences, *Bioinformatics*. 2018;**34**:3929–30.

28. Katoh K, Standley DM. MAFFT multiple sequence alignment software version 7: improvements in performance and usability. *Mol Biol Evol.* 2013;**30**:772–80.

29. Criscuolo A, Gribaldo S. BMGE (Block Mapping and Gathering with Entropy): a new software for selection of phylogenetic informative regions from multiple sequence alignments. *BMC Evol Biol*. 2010;**10**:210.

30. Kalyaanamoorthy S, Minh BQ, Wong TKF, et al. ModelFinder: Fast model selection for accurate phylogenetic estimates. *Nat Methods*, 2017;**14**:587-9.

31. Nguyen LT, Schmidt HA, von Haeseler A, et al. IQ-TREE: A fast and effective stochastic algorithm for estimating maximum likelihood phylogenies. *Mol Biol Evol.* 2015;**32**:268-74.

32. Barghi N, Concepcion GP, Olivera BM, et al. Structural features of conopeptide genes inferred from partial sequences of the *Conus tribblei* genome. *Mol Genet Genom.* 2016;**291**:411–22.

33. Hu H, Bandyopadhyay PK, Olivera BM, et al. Characterization of the *Conus bullatus* genome and its venom-duct transcriptome. *BMC Genomics.* 2011;**12**:60.

34. Andreson R,Roosaare M,Kaplinski L, et al. Gene content of the fish-hunting cone snail *Conus consors*. *bioRxiv* 2019, doi:10.1101/590695

35. Adachi K, Yoshizumi A, Kuramochi T, et al. Novel insights into the evolution of genome size and AT content in mollusks. *Mar Biol* 2021;**168**:25, doi:10.1007/s00227-021-03826-x.

36. Wenger AM, Peluso P, Rowel WJ, et al. Accurate circular consensus long-read sequencing improves variant detection and assembly of a human genome. *Nature Biotech*. 2019;**37**:1155–62.

37. Simone LRL. Phylogeny of the Caenogastropoda (Mollusca), based on comparative morphology. *Arq Zool*. 2011;**42**:161–323.

38. Osca D, Templado J, Zardoya R. Caenogastropod mitogenomics. *Mol Phylogenet Evol.* 2015.;**93**:118-28

39. Takano T, Waré A, Kano Y. Phylogenetic position of the deep-sea snail family Haloceratidae and new insights into caenogastropod relationships. *J Mollusc Stud*, 2022;**88**, doi:10.1093/mollus/eyac012.

40. Hallinan NM, Lindberg DR. Comparative analysis of chromosome counts infers three paleopolyploidies in the Mollusca. *Genome Biol Evol* . 2011;**3**:1150–63.

41. Liu C, Zhang Y, Ren Y, et al. The genome of the golden apple snail *Pomacea canaliculata* provides insight into stress tolerance and invasive adaptation. *Gigascience*. 2018;**7**:1–13, doi:10.1093/gigascience/giy101.

42. Lemarcis T, Fedosov AE, Kantor YI, et al. Neogastropod (Mollusca, Gastropoda) phylogeny: a step forward with mitogenomes. *Zool Scri*, 2022.;**51**:550-61, doi:10.1111/zsc.12552.

43. Abalde S, Tenorio MJ, Uribe JE, et al. Conidae phylogenomics and evolution. *Zool* Scr. 2019;**48**:194– 214, doi:10.1111/zsc.12329.

Table 1: Assembly statistics and annotation parameters of *K. canariensis* genome. All metrics are based on contigs of size ≥ 500 bp.

**Contig Assembly**			
	Number of Reads	31,761,787 (371.2 Gb)	
	Estimated genome size	3.6 Gb	
	Total length (bp)	2,867,696,795	
	Number of Contigs	25,961	
	Longest contig (bp)	4,977,278	
	N50 (bp)	646,466	
	N90 (bp)	59,506	
	GC (%)	43.84	
	BUSCOv5.1.3	(metazoa_odb10)	n=954
	Complete	93.1%	888
	Complete single copy	87.2%	832
	Complete duplicated	5.9%	56
	Fragmented	4.2%	40
	Missing	2.7%	26
**Scaffold Assembly**			
	Total length (bp)	2,868,115,334	
	Total No. Scaffolds	18,547	
	Scaffolds (>= 1000 bp)	18,494	
	Largest Scaffold	153,129,599	
	N50 (bp)	79,645,777	
	N90 (bp)	40,485,963	
	GC (%)	43.84	
	BUSCOv5.1.3	(metazoa_odb10)	n=954
	Complete	93.5%	892
	Complete single copy	87.5%	935
	Complete duplicated	6%	57
	Fragmented	3.8%	36
	Missing	2.7%	26
**Genome Annotation**			
Repeats Masked	Total genome masked	49.55%	
	Class I TEs repeats	18.56%	
	Class II TEs repeats	7.58%	
	Low complexity repeats	0.97%	
	Simple repeats	10.51%	
Gene Prediction	Total number of genes	34,241	
	Total coding region (bp)	37,756,940 (37.756 Mb)	
	Number of single-exon genes	10,549	
Protein assessment	BUSCOv5.1.3	(metazoa_odb10)	n=954
	Complete	81.6%	779
	Complete single copy	80.9%	772
	Complete duplicated	0.7%	7
	Fragmented	5.1%	49
	Missing	13.3%	126

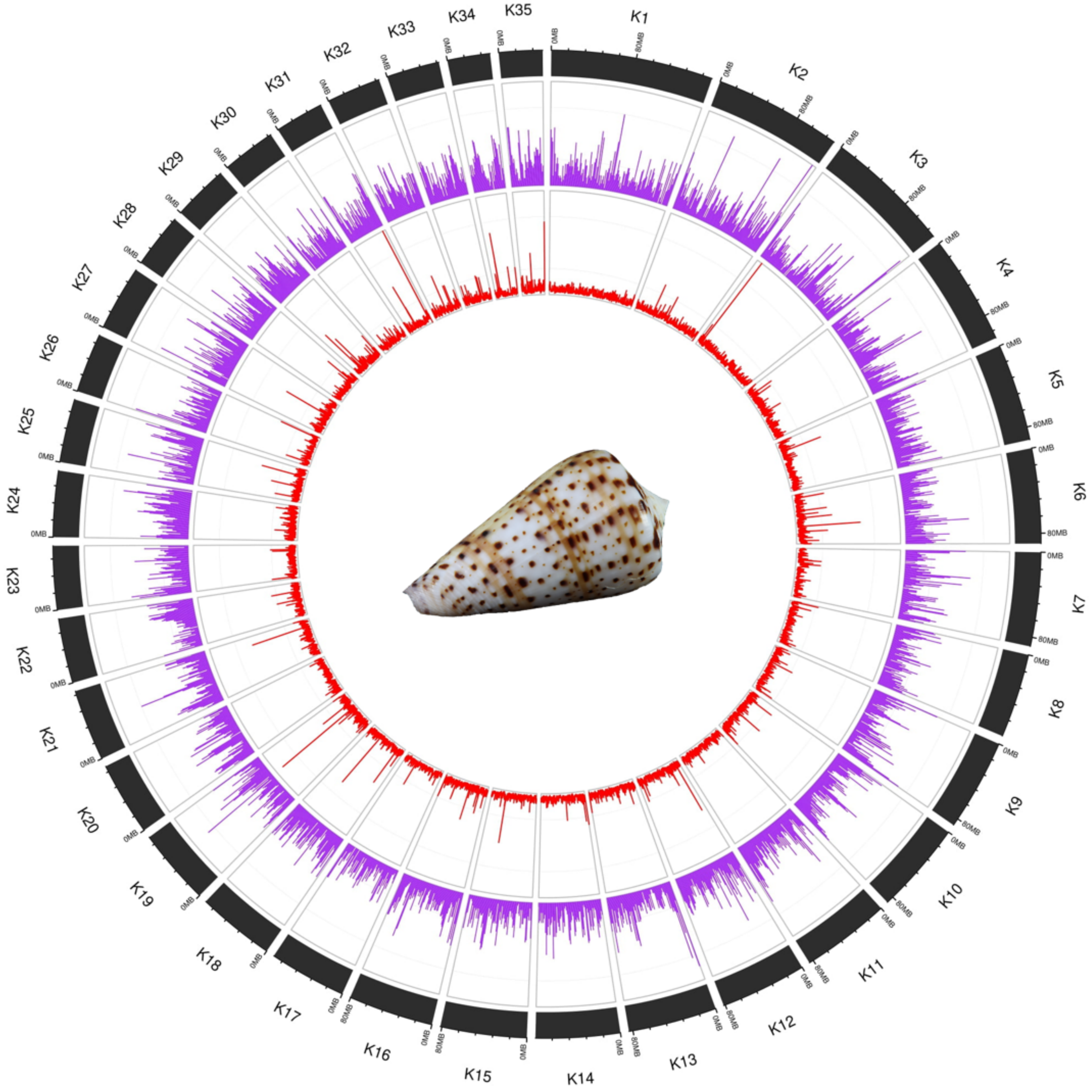
K1
K2
K3
K4
K5
K6
K7
K8
K9
K10
K11
K12
K13
K14
K15
K16
K17
K18
K19
K20
K21
K22
K23
K24
K25
K26
K27
K28
K29
K30
K31
K32
K33
K34
K35

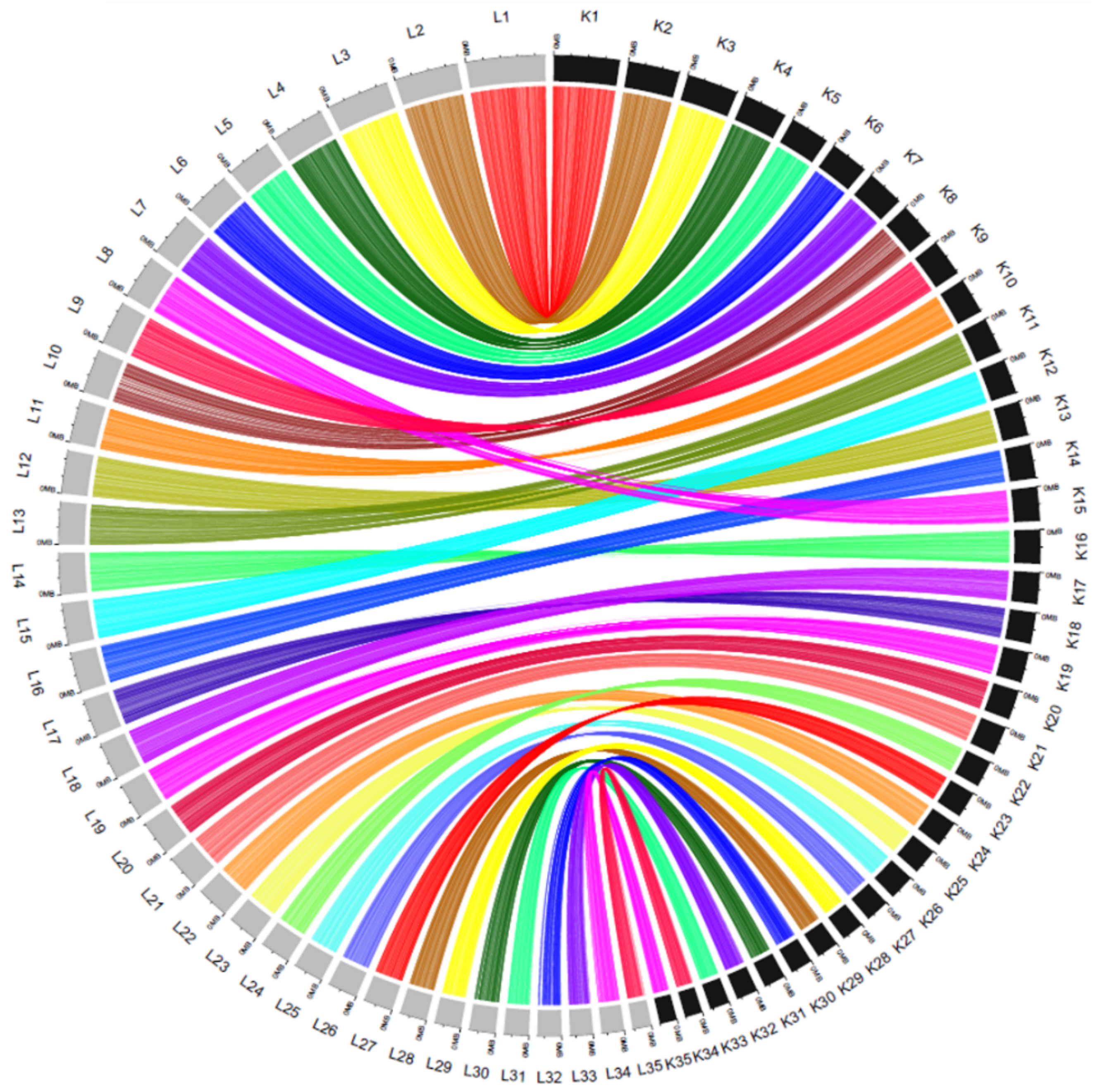
L1
L2
L3
L4
L5
L6
L7
L8
L9
L10
L11
L12
L13
L14
L15
L16
L17
L18
L19
L20
L21
L22
L23
L24
L25
L26
L27
L28
L29
L30
L31
L32
L33
L34
L35
K1
K2
K3
K4
K5
K6
K7
K8
K9
K10
K11
K12
K13
K14
K15
K16
K17
K18
K19
K20
K21
K22
K23
K24
K25
K26
K27
K28
K29
K30
K31
K32
K33
K34
K35

Click here to access/download
Supplementary Material
Supp Mat. Kcan.pdf
