# Supplementary material for: Chromosome-level genome of the venomous snail Kalloconus canariensis: a valuable model for venomics and comparative genomics
Source: Gigascience. 2023 Sep 30;12:giad075. doi: 10.1093/gigascience/giad075 (PMC10541794; doi:10.1093/gigascience/giad075)
Supplement: giad075_GIGA-D-23-00046_Revision_1 [file giad075_giga-d-23-00046_revision_1.pdf]

GigaScience

Chromosome-Level Genome of the Venomous Snail Kalloconus canariensis: A Valuable Model for Venomics and Comparative Genomics

--Manuscript Draft--

**Manuscript Number:**	GIGA-D-23-00046R1
**Full Title:**	Chromosome-Level Genome of the Venomous Snail Kalloconus canariensis: A Valuable Model for Venomics and Comparative Genomics
**Article Type:**	Data Note
**Funding Information:**	Ministerio de Ciencia e Innovación (PID2019-103947GB-C22) — Prof. Rafael Zardoya<br>Ministerio de Ciencia e Innovación (BES2017–081195) — Dr. José Ramón Pardos-Blas<br>Ministerio de Ciencia e Innovación (PRE2020-095119) — Ms Ana Herráez-Pérez
**Abstract:**	Background: Genomes are powerful resources to understand the evolutionary mechanisms underpinning the origin and diversification of the venoms of cone snails (Conidae: Caenogastropoda), and could aid in the development of novel drugs. Findings: Here, we used PacBio CLR reads and Omni-C data to assemble the chromosome-level genome of Kalloconus canariensis, a vermivorous cone endemic to the Canary Islands. The final genome size was 2.87 Gb, with a N50 of 79.75 Mb and 91 % of the reads located into the 35 largest scaffolds. Up to 55,80 % of the genome was annotated as repetitive regions, being Class I of transposable elements (16,65%) predominant. The annotation estimated 34,287 gene models. Comparative analysis of this genome with the two cone snail genomes released to date (Dendroconus betulinus and Lautoconus ventricosus) revealed similar genome sizes and organization, although chromosome sizes tended to be shorter in K. canariensis. Phylogenetic relationships within subclass Caenogastropoda were recovered with strong statistical support. The family Conidae was recovered as a clade, with K. canariensis plus L. ventricosus sister to D. betulinus. Conclusions: Despite the great diversity of cone snails (>900 species) and their venoms (hundreds of peptides per species), only two recently reported genomes are available for the group. The high-quality chromosome-level assembly of K. canariensis will be a valuable reference for studying the origin and evolution of conotoxin genes as well as whole genome duplication (WGD) events during gastropod evolution.
**Corresponding Author:**	Rafael Zardoya, PhD<br>Museo Nacional de Ciencias Naturales-CSIC<br>Madrid, Madrid SPAIN
**Corresponding Author Secondary Information:**	
**Corresponding Author's Institution:**	Museo Nacional de Ciencias Naturales-CSIC
**Corresponding Author's Secondary Institution:**	
**First Author:**	Ana Herráez-Pérez
**First Author Secondary Information:**	
**Order of Authors:**	Ana Herráez-Pérez<br>José Ramón Pardos-Blas<br>Carlos Manuel Lourenço Afonso<br>Manuel J. Tenorio<br>Rafael Zardoya, PhD
**Order of Authors Secondary Information:**	
**Response to Reviewers:**	Response to Reviewers

Powered by Editorial Manager® and ProduXion Manager® from Aries Systems Corporation

Reviewer #1:
The paper by Ana Herráez-Pérez et al. provides a high quality genome of a new Conus species. All research appears to be conducted appropriately except the authors used SPAdes for de novo transcriptome assembly tool, which has inferior performance to Trinity, particularly in venom/toxins encoding species.

We appreciate all reviewer comments and positive feedback. Regarding the choice of de novo transcriptome assembly software, comparative analyses of different assembly methods showed that rnaSPAdes does not present remarkable differences regarding quality annotation compared to Trinity (Bushmanova et al. 2019; GigaScience 8, giz100). Nonetheless, in order to address the referee suggestion, we have assembled the transcriptomes with Trinity and repeated the phylogenomic analysis, obtaining consistent results. The new phylogenomic tree has been incorporated into the article.

The authors also report using these transcriptomes to guide genome assembly but the sequences identified in the venom gland transcriptome need to be presented in the paper and discussed as part of the paper. An explanation for the lack of any venom genomes also needs to be provided, or if these were identified they need to be included in the manuscript.

We obtained RNA-seq data from three muscle (foot) and two venom glands samples of Kalloconus canariensis. These raw reads together with those from other cone snails available at SRA were used to train ab initio models for annotation. This has been clarified in the main text and in supplementary materials.
This is a Data Note, not a full research paper. Thus, we focus on describing the gene content of K. canariensis but cannot get into an in-depth analysis of venom genes and synteny comparisons with other cone snail genomes, which is the subject of a coming paper.

Reviewer #2:
The authors of the manuscript entitled "Chromosome-Level Genome of the Venomous Snail Kalloconus canariensis: A Valuable Model for Venomics and Comparative Genomics" provided the first reference genome for the species based on PacBio CLR long reads and Omni-C data. The genome presented in this work, showed very high standards in terms of contiguity (chromosome level) and completeness. Overall, the manuscript is well explained and data generated will be very useful, not only for researchers interested in this group of molluscs, but any interested in venomics.

We thank the reviewer for the kind words and the constructive suggestions.

However, I have a couple of issues that in my opinion should be addressed before the acceptance of the manuscript. First of all, I would strongly recommend to include some kind of contamination analysis (e.g. blobtools https://github.com/DRL/blobtools). It is something fast and easy to run and could discard any contings-sccaffolds that come from other organisms. I don't think that data here presented showed any problem of contamination, but I think is an approach that should always be done to check the quality of raw data.

Following the suggestion of the referee we checked potential contaminations with BlobToolKit. In the revised version, we describe this analysis in the main text and include the blobplot as supplementary figure S5.

My second major concern is related to annotation quality, that showed a 13% of missing BUSCOs. The high contiguity of the genome here provided, with very good completeness (only 2.7% of missing BUSCOs), makes me think that the annotation could be improved. This idea is also supported by the use of an old version of Augustus (v2.5.5.), because since many time ago the version 3.0 is available and it has showed to recover better predictions. In any case, I would recommend to authors to try another annotation pipelines such as GeMoMa, EVidenceModeller or BRAKER2 to try to recover a better annotation scheme.

Following the suggestion of the reviewer, we ran an alternative annotation with BRAKER2. This is now mentioned in the 'genome annotation' section of Materials &

	Methods (pages 5-6, new lines 24-5). New results and discussion are also included in page 9, lines 7-22.<br><br>I also have attached some minor comments in the pdf version of the main manuscript and supplementary material that could help to improve the manuscript:<br>Here I would be more precise saying something like: "Their genomes sizes are comparable and range from 2.87 to 3.59 Gb". This difference of 700 Mb is important since there are many species (including mollucks, e.g. Chrysomallon squamiferum) that they have genome sizes within this value.<br><br>We agree with the reviewer. Changed as suggested in page 3, new lines 18-19.<br><br>You could also say "we collected the specimens off the coast of Tenerife" to be more specific about the location. Giving geographical coordinates could also be helpful in providing precise information about where the specimens were collected.<br><br>Page 3, new lines 24-25 "...were collected in Playa de Porís, Tenerife, Canary Islands (Spain; GPS coordinates: 28.16447, -16.43185) in September 2020."<br><br>Remove and<br><br>Removed! (page 4, line 3).<br><br>I consider this step fundamental. Did you provide any estimated genome size? any other parameter? if not indicate that was run under default parameters.<br><br>As suggested, the clarification is now added in page 4, lines 21-22 "using wtdgb2 v.2.5 with default parameters."<br><br>Why did you select directly this tool? Did you check any other long-reads assemblers such as flye or canu?<br><br>As the reviewer mention, there are multiple alternatives for genome assembly based on long reads. Wtdgb2 was chosen because it provides faster assembly speeds for large genomes with comparable contiguity and assembly accuracy and tend to have less duplicates (Ruan & Li, 2020; Nat Methods 2020 17, 155-158), making it a good choice for our genome. Besides that, it provided us with excellent results when assembling the genome of Lautoconus ventricosus (the reasons for choosing Wtdgb2 are now mentioned in the main text (page 4, lines 22-23).<br><br>In my opinion, the approach used for transcriptome assembly is not clear. Did you combine all foot and venom glands from the 5 specimens together to obtain two transcriptomes (foot and venom)? or did you assembled all transcriptomes independently?<br><br>Actually, the reviewer is right and the procedure was not well explained. We use all raw reads from three muscle (foot) and two venom glands to directly guide the genome annotation with both MAKER and BRAKER2. Thus, we have modified the statement accordingly (page 5, lines 7-8). "RNA-sequence reads of three muscle (foot) and two venom glands of K. canariensis were pooled together and used for gene annotation."<br><br>This is a pretty old version of Augustus (March 2012)... could be the reason why you have this low BUSCO values in the annotation? I strongly recommend to use and updated version of augustus (3.4; 3.5…)<br><br>Following the suggestion of the reviewer, we updated to v3.5.0 of Augustus and reran the annotation step. The new assembly annotation retrieves 90.9% of complete genes, 6.1 % of fragmented genes and 3.0% of missing genes. The main text of the paper has been updated accordingly.<br><br>I think is not clear how you assembled transcriptomes. See my previous comment.<br><br>Following the advice of the reviewer, we have clarified the procedure that we followed for the assembly of the transcriptomes that were used for phylogenomic reconstruction.

	We now explain in the M&M section: "RNA-seq raw reads retrieved from SRA NCBI were assembled using Trinity v2.12.0 to generate the transcriptome of each of the 18 species."<br><br>This model was obtained with IQ-TREE or did you use other tool such as PartitionFinder or jModelTest?<br><br>We now explain in page 7, lines 20-22 "The ModelFinder module was used to select LG+R4 as the best-fit model, according to the Bayesian Information Criterion (BIC)."<br><br>Protein Coding Genes?<br><br>Changed by "genes" in page 9, line 12<br><br>I would say by aligning the translated proteins against the genome assembly<br><br>This statement was deleted, as we made a new genome annotation recovering a higher percentage of genes.<br><br>Taking into account that you recovered a high-quality genome assembly, I found this value really low. In the table S4 that you provide, not many species have 10% of missing BUSCOs (and two that have that values are at contig level).<br><br>We agree with the reviewer. The new annotation has a lower percentage (3%) of missing BUSCOs. The text has been changed accordingly.<br><br>It is true but the only reference at chormosome level has 100% of BUSCO. There are other gastropoda genomes assemblies in other databases at chromosome level. It would be interesting if it something really extraordinary of Pomacea. Chrysomallon squamiferum: https://datadryad.org/stash/dataset/doi:10.5061/dryad.24053dn; Achatina fulica: http://gigadb.org/dataset/100647<br><br>We agree with the reviewer, now we include in table S4 the BUSCOs for the proteins of Chrysomallon squamiferum (86.3% with eukaryota odb10 and 83.8% with metazoa odb10) and Achatina fulica (57.3% with eukaryota odb10 and 59.7% with metazoa odb10).<br><br>This value could indicate that annotation is not as good as expected...<br>As we mentioned before, these values have been improved with the new annotation. We now included it in Table 1.
**Additional Information:**	
**Question**	**Response**
Are you submitting this manuscript to a special series or article collection?	No
**Experimental design and statistics**<br><br>Full details of the experimental design and statistical methods used should be given in the Methods section, as detailed in our Minimum Standards Reporting Checklist. Information essential to interpreting the data presented should be made available in the figure legends.<br><br>Have you included all the information requested in your manuscript?	Yes

Powered by Editorial Manager® and ProduXion Manager® from Aries Systems Corporation

**Resources**<br><br>A description of all resources used, including antibodies, cell lines, animals and software tools, with enough information to allow them to be uniquely identified, should be included in the Methods section. Authors are strongly encouraged to cite Research Resource Identifiers (RRIDs) for antibodies, model organisms and tools, where possible.<br><br>Have you included the information requested as detailed in our Minimum Standards Reporting Checklist?	Yes
**Availability of data and materials**<br><br>All datasets and code on which the conclusions of the paper rely must be either included in your submission or deposited in publicly available repositories (where available and ethically appropriate), referencing such data using a unique identifier in the references and in the “Availability of Data and Materials” section of your manuscript.<br><br>Have you have met the above requirement as detailed in our Minimum Standards Reporting Checklist?	Yes

Manuscript

Click here to access/download;Manuscript;Kcanariensis_report_GigaScienc

To be submitted to:
GigaScience
Data Note
Version: 04/07/2023

Chromosome-Level Genome of the Venomous Snail *Kalloconus canariensis*: A Valuable Model for Venomics and Comparative Genomics

Ana Herráez-Pérez[1], José Ramón Pardos-Blas[1], Carlos M.L. Afonso[2], Manuel J. Tenorio[3] and Rafael Zardoya[1]*

[1] Departamento de Biodiversidad y Biología Evolutiva, Museo Nacional de Ciencias Naturales (MNCN-CSIC), José Gutiérrez Abascal 2, 28006 Madrid, Spain.

[2] Centre of Marine Sciences (CCMAR), Universidade do Algarve, Campus de Gambelas, 8005–139 Faro, Portugal.

[3] Departamento CMIM y Q. Inorgánica-INBIO, Facultad de Ciencias, Universidad de Cádiz, 11510 Puerto Real, Cádiz, Spain.

* **Correspondence address.** Rafael Zardoya, Departamento de Biodiversidad y Biología Evolutiva, Museo Nacional de Ciencias Naturales (MNCN-CSIC), José Gutiérrez Abascal 2, 28006 Madrid, Spain. E-mail: rafaz@mncn.csic.es

Ana Herráez-Pérez [0000-0003-3061-4890];

José Ramón Pardos-Blas [0000-0001-7139-3153];

Carlos Manuel Lourenço Afonso [0000-0002-9084-2177];

Manuel J Tenorio [0000-0003-4088-4958];

Rafael Zardoya [0000-0001-6212-9502].

Abstract

Background: Genomes are powerful resources to understand the evolutionary mechanisms underpinning the origin and diversification of the venoms of cone snails (Conidae: Caenogastropoda), and could aid in the development of novel drugs. **Findings:** Here, we used PacBio CLR reads and Omni-C data to assemble the chromosome-level genome of *Kalloconus canariensis*, a vermivorous cone endemic to the Canary Islands. The final genome size was 2.87 Gb, with a N50 of 79.75 Mb and 91 % of the reads located into the 35 largest scaffolds. Up to 55,80 % of the genome was annotated as repetitive regions, being Class I of transposable elements (16,65%) predominant. The annotation estimated 34,287 gene models. Comparative analysis of this genome with the two cone snail genomes released to date (*Dendroconus betulinus* and *Lautoconus ventricosus*) revealed similar genome sizes and organization, although chromosome sizes tended to be shorter in *K. canariensis*. Phylogenetic relationships within subclass Caenogastropoda were recovered with strong statistical support. The family Conidae was recovered as a clade, with *K. canariensis* plus *L. ventricosus* sister to *D. betulinus*. **Conclusions:** Despite the great diversity of cone snails (>900 species) and their venoms (hundreds of peptides per species), only two recently reported genomes are available for the group. The high-quality chromosome-level assembly of *K. canariensis* will be a valuable reference for studying the origin and evolution of conotoxin genes as well as whole genome duplication (WGD) events during gastropod evolution.

Keywords: Cone snails, *Kalloconus canariensis*, chromosome-level genome, comparative genomics, Omni-C.

Background

Cones (Caenogastropoda: Conidae) are venomous marine snails that live in tropical and subtropical seas worldwide [1]. Cones produce complex venoms to capture worms, snails, and fishes, as well as to defend against predators [2, 3]. The venom is composed of short peptides termed conotoxins, which directly block ion channels and neuromuscular receptors in their preys and thus, are the subject of intense research for novel drug development and disease treatment [4, 5]. Transcriptomics and proteomics of cone venom ducts have revealed an extraordinary diversity in the composition of venom cocktails. High-throughput long-read sequencing has opened the door to scaffold cone genomes to the chromosome level. As more cone genomes are assembled, it will be possible to perform detailed comparative genomics studies, which have the potential to unravel key details about the genetic basis of conotoxin diversity and evolution.

Here, we report *de novo* chromosome-level genome assembly of a vermivorous cone snail endemic to the Canary Islands, *Kalloconus canariensis* (NCBI:txid2750724) [6], and compare it with the only two other Conidae genomes released thus far, those of *Dendroconus betulinus* [7] and *Lautoconus ventricosus* [8]. The comparison of these three high-quality assemblies allows for the first time inferring patterns of genome evolution within this group. Their genome sizes are comparable and range from 2.87 to 3.59 Gb as well as have similar organization (into 35 pseudo-chromosomes), showing long stretches of conserved synteny.

Materials and Methods

Sample collection

Five specimens of *K. canariensis* were collected in Playa de Porís, Tenerife, Canary Islands (Spain; GPS coordinates: 28.16447, -16.43185) in September 2020. Each individual was taken out of the shell and dissected to collect foot muscle and venom gland. The foot muscles were flash frozen in liquid nitrogen and stored at -80ºC, for subsequent high molecular weight (HMW) DNA extraction. The venom glands were preserved in RNAlater (Thermo Fisher Scientific, Waltham, MA, USA) at -20ºC, for transcriptome assembly and genome annotation.

Genome sequencing

The HMW DNA extraction from the foot, library preparation, long-read sequencing, contig assembly and scaffolding were performed by Dovetail Genomics (Scotts Valley, CA, USA) as previously described [8], except that Chicago+HiC libraries were substituted by an OmniC library, which ensures a more homogeneous digestion of chromatin and coverage (see Supplementary methods, for full details).

Long-read sequencing of HMW DNA isolated from individual TF39 (the shell was deposited as voucher in the MNCN collection under accession number (MNCN15.05/94850) was performed on PacBio Sequel II Single Molecule, Real-Time (SMRT) cells using the Continuous Long Read (CLR) sequencing mode. A Dovetail Omni-C library to obtain proximity ligation data was generated and was sequenced on an Illumina HiSeqX (see Supplementary methods).

Genome assembly and scaffolding

Long-read CLR sequences were *de novo* assembled using wtdgb2 v.2.5 [9] with default parameters. This software provides faster assembly speeds for large genomes with comparable contiguity and assembly accuracy and tend to have less duplicates than

other assemblers [9]. The data from the *de novo* assembly and the OmniC library were used for scaffolding using HiRiSE™ [10]. QUAST (QUAST, RRID:SCR_001228) v5.0.2 [11] and BUSCO (BUSCO, RRID:SCR_015008) v5.1.3. [12] were used to obtain general metrics and completeness assessment of the final genome assembly, respectively. Potential sources of DNA contamination were checked with BlobToolkit (BlobToolKit, RRID:SCR_023351) v4.1.5. [13].

Genome annotation

RNA-sequence reads of three muscle (foot) and two venom glands of *K. canariensis* specimens were generated and pooled together for gene annotation. Repeat families were identified *de novo* and classified using RepeatModeler (RepeatModeler, RRID:SCR_015027) v2.0.1. [14]. These regions were masked with RepeatMasker (RepeatMasker, RRID:SCR_012954) v4.1.0 [15] for further genome analyses. RNA-seq data from other cone snail species downloaded from the SRA database and the newly generated RNA-seq reads from *K. canariensis* were used to train two independent *ab initio* models for annotation using SNAP v2006-07-28 [16] and AUGUSTUS (Augustus, RRID:SCR_008417) v2.5.5 [17], respectively. RNA-seq reads were mapped onto the genome using the STAR (STAR, RRID:SCR_004463) v2.7 aligner software [18] and intron hints were generated with bam2hints tools within AUGUSTUS. Gene predictions were made using both SNAP and AUGUSTUS (with intron-exon boundary hints provided from RNA-Seq) with MAKER (MAKER, RRID:SCR_005309) v3.01.01 [19] (see Supplementary methods).

In addition, an alternative annotation was performed with BRAKER v2.1.6 [20 - 30]. The clean reads after trimmomatic of each of the samples (three feet and two venom glands) of *K. canariensis* were map onto the genome assembly using STAR v2.7.10 and

combined into a single sorted bam file. A set of proteins was incorporated to the analysis from the annotation of *L. ventricosus* and the Metazoa dataset from OrthoDB v.11 (OrthoDB, RRID:SCR_011980), which are both partitions available for direct running in BRAKER2 (in -etpmode). Completeness of the annotated gene models was assessed with BUSCO v5.1.3. and metazoan OrthoDB v. 10 [12].

Synteny analyses

All analyses were based exclusively on the 35 pseudo-chromosomes (Fig. 1, Supplementary Table S1 and Fig. S1). Gene annotations of the *K. canariensis* and *L. ventricosus* genomes were used to compare number and length of genes, exons and intergenic regions in both genomes (Supplementary Table S2 and Fig. S2). The comparison with the *D. betulinus* genome was not possible, as the annotation of genes per pseudo-chromosome was not reported [7].

The genomes of *K. canariensis* and *L. ventricosus* were aligned using Minimap2 v2.24-r1122 (Minimap2, RRID:SCR_018550) [31] to infer homologue scaffolds between both species (Fig 2 and Supplementary Fig. S3). Each scaffold of *K. canariensis* was mapped onto its homologue of *L. ventricosus* with Satsuma2 [32]. A synteny plot was generated with shinyCircos [33] for whole genome comparison between both species (Fig. 2), and D-genies (D-genies, RRID:SCR_018967) v1.3.1 [34] was used for pairwise scaffold analysis (Supplementary Fig. S3). To simplify whole genome plots, matches <0.8 of identity and short links <1kb were filtered out and adjacent links (within 10 Mb) were merged using bundlelinks [33].

Phylogenomic Tree Reconstruction

A phylogeny of Caenogastropoda was reconstructed based on sequence data from the genomes of *K. canariensis* and *L. ventricosus* plus RNA-seq data downloaded from the Sequence Read Archive (SRA) at NCBI from another 16 caenogastropods, plus one Heterobranchia (*Fiona pinnata*) and one Neritimorpha (*Nerita melanotragus*) as outgroup taxa (Supplementary Fig. S4 and Table S3).

RNA-seq raw reads retrieved from SRA NCBI were assembled using Trinity (Trinity, RRID:SCR_013048) v2.12.0 [35] to generate the transcriptome of each of the 18 species. For each transcriptome, the longest ORFs were predicted and translated into protein sequences using TransDecoder (TransDecoder, RRID:SCR_017647) v5.5.0 [36] with default settings. Protein sets were clustered and isoforms were removed with CD-HIT (CD-HIT, RRID:SCR_007105) v4.8.1 [37] using default options and a sequence identity threshold of 0.98. Orthogroups were inferred from the protein sets using Orthofinder (OrthoFinder, RRID:SCR_017118) v2.5.4 [38]. Single-copy orthogroups shared by at least 18 of the 20 species (occupancy of 90%) were selected using Prequal v.1.02 [39] and aligned using MAFFT (MAFFT, RRID:SCR_011811) v7.487 [40]. Phylogenetic informative regions were selected using BMGE v 1.12 [41].

Phylogenomic analyses based on curated amino acid alignments were performed using IQ-TREE (IQ-TREE, RRID:SCR_017254) v2.1.2 [42, 443] under maximum likelihood (ML) with 1000 ultrafast bootstrap pseudo-replicates. The ModelFinder module of IQ-TREE [42] was used to select LG+R4 as the best-fit model, according to the Bayesian Information Criterion (BIC).

Results and Discussion

Genome assembly, and scaffolding

The chromosome–level genome of *K. canariensis* was assembled and scaffolded using 371.2 Gb of PacBio CLR reads (130x coverage) and 81.1 Gb of Omni-C paired-end reads (28x coverage), respectively. PacBio long reads were *de novo* assembled into 25,961 contigs (the longest was 4.98 Mb; N50 was 646.47 kb). The HiRise scaffolding led to 18,572 scaffolds (the largest was 153.13 Mb; N50 was 79.65 Mb; see Table 1). Little signature of potential exogenous DNA contamination from bacteria was detected using the BlobToolKit viewer [13] v4.1.0 (Supplementary Fig. S5).

Table 1: Assembly statistics and annotation parameters of *K. canariensis* genome. All metrics are based on contigs of size ≥ 500 bp.

The final genome assembly was 2.87 Gb in length, which is smaller than those of *D. betulinus* (3.43 Gb) [7] and *L. ventricosus* (3.59 Gb) [8], but similar to the inferred genome sizes of *Kioconus tribblei* (2.76 Gb) [44] and *Textilia bullata* (2.56 Gb) [45].

The final scaffolding grouped 91.2% of the total assembled contigs into 35 scaffolds or pseudo-chromosomes, which varied in size from 153 to 40 Mb (Fig. 1 and Supplementary Fig. S1). BUSCO scores were used to assess genome completeness [12]. A total of 892 complete genes (93.5%) of the Metazoan ortholog database (odb 10) were recovered (Table 1), a value that is higher than those reported for *D. betulinus* (89.8%) [7] and *L. ventricosus* (84.9%) [8], indicating an overall higher quality of the newly reported assembly. These BUSCO metrics are also consistently higher than those inferred for *Pionoconus consors* [466] and *Kioconus tribblei* [44] (Supplementary Fig. S6).

Figure 1: Chromosome-level genome organization of *K. canariensis*. The 35 pseudo-chromosomes are shown in black. In the inner rings, gene density in purple (percentage of genes per Mb, normalized to the maximum number of genes, about 60) and coverage in red (median coverage per 10 kb, normalized to sequencing depth, 130x).

Genome annotation

Repeat regions occupied 50.80% of the total genome, with Class I and II of transposable elements (TEs) and simple repeats representing 16.65, 6.06, and 11.19%, respectively (Table 1). These proportions matched well with those of *L. ventricosus* (53.36% of the genome) [8] but were higher than those reported for the *D. betulinus* genome (38.56% of the total assembly) [7].

Gene annotation predicted a total of 34,250 genes in the MAKER genome annotation, which occupied 37.76 Mb (1.32% of the genome). While in the BRAKER genome annotation, the total protein coding genes predicted were to 34,287 (50.35 Mb, 1.76% of the genome). The number of genes was similar in the *L. ventricosus* genome (32,675 [8]) but considerably lower in the *D. betulinus* genome (22,698 [7]). The MAKER/BRAKER genome annotations contained 779 (81.6%)/ 867 (90.9%) single copy and 7 (0.7 %)/ 82 (8.6%) duplicated complete genes, as well as 49 (5.10 %)/ 58 (6.1%) fragmented genes of the BUSCO Metazoan ortholog database (odb) 10, respectively (table 1). These BUSCO metrics were similar to other molluscan genomes available at NCBI (Supplementary Table S4).

Genome organization

Each pseudo-chromosome of *K. canariensis* had a counterpart in *L. ventricosus*, thus revealing the same genome organization and conserved macrosynteny (Fig. 2).

Homologous pseudo-chromosomes in *K. canariensis* were consistently shorter than in *L. ventricosus*, being this difference particularly pronounced in pseudo-chromosomes 15 and 35 of *K. canariensis*, which were 22.45% and 23.17% smaller, respectively (Supplementary Table S1). Yet, the number of genes predicted per homologous pseudo-chromosome was slightly higher in 28 out of the 35 pseudo-chromosomes of the *K. canariensis* genome (Supplementary Table S2 and Fig. S2).

Figure 2: Plot of conserved synteny of the 35 pseudo-chromosomes between *K. canariensis* (right black; K1 to K35) and *L. ventricosus* (left grey; L1 to L35).

Gene structure

The average length of complete genes (including exons plus introns) was 41% smaller in *K. canariensis*. The genes in *K. canariensis* had fewer (10% less) but larger (8% more) exons (Supplementary Table S2 and suppl. Fig. S2). Total intergenic regions added up to 2.48 Gb in the *K. canariensis* genome, whereas this number was 14.52% higher in the *L. ventricosus* genome. Therefore, differences in length between both genomes were concentrated in the intergenic regions, likely associated to transposable elements and other repetitive regions, as has been previously shown in other mollusks [47]. Further comparative analyses on repetitive landscapes between cone genomes should shed light on the nature of expansions and contractions of repetitive elements and their potential association to genome dynamics and evolution.

Gene synteny

Pairwise comparisons between homologous pseudo-chromosomes of *K. canariensis* and *L. ventricosus* showed longer stretches of synteny in several of the largest pseudo-

chromosomes (e.g., 1, 3, and 6), whereas the smallest ones tend to present more dynamic regions with lower synteny levels (e.g., 26, 30 to 35). The comparison of syntenic regions also revealed potential rearrangements within scaffolds, including large inversions, which were located in the central (e.g., 4, 5, 8, and 11) or at one terminal (e.g., 2, 15, 16, and 29) regions (Supplementary Fig. S3). However, it was not possible to identify specific contigs that map across the inversion points and thus, this result will need further confirmation as more cone genomes are generated using sequencing technologies with higher accuracy such as PacBio HiFi [48]. In any case, the high degree of synteny conservation among the three cone genomes offers an excellent opportunity to study the birth and death of many different gene families, and understand their evolutionary dynamics. In particular, they will be crucial for uncovering the main processes underpinning the generation of conotoxin diversity, which will be reported elsewhere. Furthermore, as more chromosome-level genomes of cones are reported, it will be possible also to identify those genomic regions associated with diversification and adaptation.

Phylogenomic reconstruction

There have been different attempts to reconstruct phylogenetic relationships among caenogastropod orders based on morphology (e.g., [49]), mitogenomes (e.g., [50]), and few fragments of nuclear rRNA and mitochondrial genes (e.g., [51]), but they have proven to be difficult to resolve. Here, a maximum likelihood (ML) phylogeny of Caeogastropoda was reconstructed based on 57 single copy proteins and 18 species of caenogastropods representing 12 orders (Supplementary Table S3). The reconstructed phylogeny showed maximal or strong (>90%) statistical support in all but two nodes (Supplementary Fig. S4). The order Ampullarioidea was recovered as the first diverging

branch among the caenogastropod taxa analyzed. The next order that branched off was Cerithioidea. The order Truncatelloidea was sister to two clades: (1) Epitonioidea sister with low support to a well-resolved monophyletic group including Abyssochrysoidea sister to Littorinoidea plus Naticoidea; (2) Stromboidea sister to Velutionoidea plus Neogastropoda (Supplementary Fig. S4). The relative phylogenetic positions here recovered are totally consistent with those based on mitogenomes [50] and a combined data set of partial rRNA and mitochondrial sequences [51] but having stronger statistical support, and thus providing a robust phylogenetic framework for evolutionary studies within Caenogastropoda. For instance, it is useful for testing in combination with chromosomal-level genomes a predicted WGD event that predated the origin of Neogastropoda [52]. The number of chromosomes (35 in both cone species *versus* 14 in the caenogastrop *Pomacea canaliculata* [53]) together with the synteny relationships between *K. canariensis* and *L. ventricosus* (this work) and between *L. ventricosus* and *P. canaliculata* [8] strongly support such WGD event (Supplementary Fig. S4).

Another controversy within Caenogastropoda is related with the monophyly and internal phylogenetic relationships at the superfamily level of Neogastropoda [50, 54]. Here, the monophyly of Neogastropoda is recovered with maximal support, although many important superfamilies are missing, and thus we cannot add new insights on the relative phylogenetic position of superfamilies such as Volutoidea, Tonnoidea and Ficoidea, which are the center of a long-standing debate [50, 54]. The monophyly of the superfamily Conoidea received maximal support and within the family Conidae, *D. betulinus* was recovered as sister to *L. ventricosus* plus *K. canariensis*, in agreement with previous phylogenies based on mitogenomes [55].

Conclusions

Until now only two genomes were available for cone snails. In this study, we provide a high-quality chromosome-level assembly of *Kalloconus canariensis*, an endemic cone snail of the Canary Islands. This new genome represents a valuable resource for comparative genomics among venomous gastropods, which are essential to understand the genetic basis of the origin and diversification of toxins and its use in novel drug development. Moreover, this annotated genome would serve as a helpful support for the assembly of other genomes within the family Conidae. Finally, because of the lack of genomic resources available for gastropods, this cone snail genome will be useful for evolutionary studies in gastropod evolution.

Additional Files

Supplementary Methods: Whole-genome assembly, scaffolding, and annotation of *Kalloconus canariensis.*

Supplementary Figure S1: Link density histogram (35 pseudochromosomes).

Supplementary Figure S2: Differences in gene and intergenic regions lengths between *L. ventricosus* (left blue, Lven) and *K. canariensis* (right orange, Kcan).

Supplementary Figure S3: Synteny maps between *L. ventricosus* (L1 to L35) and *K. canariensis* (K1 to K35) pseudo-chromosomes.

Supplementary Figure S4: Maximum likelihood phylogenomic relationships of Caenogastropoda (green branches) based on 57 single copy proteins of 20 gastropod species. Neritimorpha and Heterobranchia were used as outgroups. Neogastropoda and Conidae are depicted with slashed purple and red boxes, respectively. Numbers at nodes are boostrap support values. The circle represents an ancestral Whole Genome Duplication (WGD) event [52]. The reconstructed tree was visualized with FigTree v1.4.4 (FigTree, RRID:SCR_008515) .

Supplementary Figure S5: DNA sources in the *Kalloconus canariensis* genome.

Supplementary Figure S6: BUSCO assessment of the available assembled genomes of Cone Snails. BUSCO v.5.1.3. and Metazoan Ortholog Database (odb10, n=954) were used.

Supplementary Table S1: Sizes of homologous pseudo-chromosomes of *K. canariensis* and *L. ventricosus*.

Supplementary Table S2: Comparison of number and length of genes and intergenics regions of homologous pseudo-chromosomes of *K. canariensis* and *L. ventricosus*.

Supplementary Table S3: Taxonomy of gastropods and source of sequences included in phylogenomic analyses.

Supplementary Table S4: Genome assembly and BUSCO metrics of genome annotations of molluscs available at NCBI.

Data Availability

Raw reads are available at SRA-NCBI under BioProject PRJNA843968 with accession numbers SRR19919783 and SRR20083570 for PacBio and OmniC reads, respectively. The Whole Genome Shotgun project has been deposited at GenBank under accession number JAMYXO000000000. All supporting data and materials are available in the *GigaScience* GigaDB [56].

Abbreviations

BMGE: Block Mapping and Gathering Entropy; BIC: Bayesian Information Criterion; BLAST: Basic Local Alignment Search Tool; BUSCO: Benchmarking Universal Single-Copy Orthologs; CLR: continuous long read; Gb: gigabase pairs; HiFi: High-Fidelity; HMMs: Hidden Markov Models; HMW: high-molecular weight; kb: kilobase pairs; MAFFT: Multiple Alignment using Fast Fourier Transform; Mb: megabase pairs;

ML: maximum likelihood; NCBI: National Center for Biotechnology Information; ORF: open reading frame; PacBio: Pacific BioSciences; QUAST: QUality ASsessment Tool; RNA-seq: RNA sequencing; rRNA: ribosomal RNA; SMRT: Single Molecule Real-Time; SNAP: Semi-HMM-based Nucleic Acid Parser; SRA: Sequencing Read Archive; STAR: Spliced Transcripts Alignment to a Reference; TE: transposable element; WGD: whole-genome duplication.

Competing Interests

The authors declare that they have no competing interests

Funding

This work was supported by the Spanish Ministry of Science and Innovation [PID2019-103947GB-C22 to R.Z.; BES2017–081195 to J.R.P.-B. and PRE2020-095119 to A.H.-P].

Authors' Contributions

R.Z. conceived the study and designed the analyses. M.J.T. and C.M.L.A. obtained the individuals, performed sample dissections, and provided information on cone snail biology. A.H.-P. and J.R.P.-B performed the bioinformatics analyses. R.Z. wrote the manuscript initial draft, and all authors read, revised, and approved the manuscript final version.

Acknowledgments

We are grateful to Richard Lewis and Luis J. Chueca for providing insightful comments on an earlier version of the manuscript. We thank Juan E. Uribe for help during field

sampling and with phylogenomic reconstruction. We are grateful to Vadim A. Pisarenco and Julio Rozas for help with synteny analysis, contamination check and providing access to the Hercules computer cluster, respectively.

References

1. Tucker JM, Tenorio MJ. Illustrated catalog of the living cone shells. MdM Publishing: Wellington; 2013.
2. Dutertre S, Jin A-H, Vetter I, et al. Evolution of separate predation- and defence-evoked venoms in carnivorous cone snails. *Nat commun*. 2014;**5**:3521.
3. Puillandre N, Bouchet P, Duda TF, et al. Molecular phylogeny and evolution of the cone snails (Gastropoda, Conoidea). *Mol Phylogenet Evol*. 2014:**78**:290-303.
4. Lewis RJ, Dutertre S, Vetter I, et al. *Conus* venom peptide pharmacology. *Pharmacol Rev*. 2012;**64**:259–98.
5. Robinson SD, Norton RS. Conotoxin gene superfamilies. *Mar Drugs*. 2014;**12**:6058-101.
6. Tenorio MJ, Abalde S, Pardos-Blas JR, et al. Taxonomic revision of West African cone snails (Gastropoda: Conidae) based upon mitogenomic studies: implications for conservation. *Eur J Taxon.* 2020;**663**:1-89.
7. Peng C, Huang Y, Bian C, et al. The first *Conus* genome assembly reveals a primary genetic central dogma of conopeptides in *C. betulinus. Cell Discov*. 2021;**7**:11.
8. Pardos-Blas JR et al. The genome of the venomous snail *Lautoconus ventricosus* sheds light on the origin of conotoxin diversity. *GigaScience*. 2021;**10**, doi: 10.1093/gigascience/giab037.
9. Ruan J, Li H. Fast and accurate long-read assembly with wtdbg2. *Nat Methods*. 2020;**17**:155–8.
10. Putnam NH, O’Connell BL, Stites JC, et al. Chromosome scale shotgun assembly using an in vitro method for long-range linkage. *Genome Res*. 2016;**26**:342–50.

11. Gurevich A, Saveliev V, Vyahhi N, et al. QUAST: quality assessment tool for genome assemblies. *Bioinformatics*. 2013;**29**(8):1072–5.

12. Manni M, Berkeley MR, Seppey M, et al. BUSCO update: novel and streamlined workflows along with broader and deeper phylogenetic coverage for scoring of eukaryotic, prokaryotic, and viral genomes. *Mol Biol Evol*. 2021;**38**(10): 4647-54.

13. Challis R, Richards E, Rajan J, et al. BlobToolKit – Interactive Quality Assessment of Genome Assemblies. *G3*. 2020;**10**:4:1361–74. doi:10.1534/g3.119.400908

14. Flynn JM, Hubley R, Goubert C, et al. RepeatModeler2 for automated genomic discovery of transposable element families. *Proc Natl Acad Sci USA*. 2020;**117**(17):9451–7.

15. Smit AFA, Hubley R, Green P. RepeatMasker Open-4.1.0. Available from: http://www.repeatmasker.org/. 2019-2020.

16. Korf I. Gene finding in novel Genomes. *BMC Bioinformatics*. 2004;**5**:59

17. Stanke M, Steinkamp R, Waack S, et al. AUGUSTUS: a web server for gene finding in eukaryotes. *Nucleic Acids Res*. 2005;**32**:W309–12.

18. Dobin A, Davis CA, Schlesinger F, et al. STAR: ultrafast universal RNA-seq aligner. *Bioinformatics*. 2013;**29**:15–21.

19. Holt C, Yandell M. MAKER2: an annotation pipeline and genome-database management tool for second-generation genome projects. *BMC Bioinformatics*. 2011;**12**:491.

20. Hoff KJ, Lange S, Lomsadze A, et al. BRAKER1: unsupervised RNA-Seq-based genome annotation with GeneMark-ET and AUGUSTUS. *Bioinformatics*. 2016;**32**(5):767-9.

21. Bruna T, Hoff KJ, Lomsadze A, et al. BRAKER2: Automatic Eukaryotic Genome Annotation with GeneMark-EP+ and AUGUSTUS Supported by a Protein Database. *NAR Genomics and Bioinformatics*. 2021;**3**(1), lqaa108.

22. Hoff KJ, Lomsadze A, Borodovsky M, et al. Whole-genome annotation with BRAKER. *Gene prediction: methods and protocols*. 2019;65-95.

23. Bruna T, Lomsadze A, Borodovsky M. GeneMark-EP+: eukaryotic gene prediction with self-training in the space of genes and proteins. *NAR Genomics and Bioinformatics*. 2020;**2**(2), lqaa026.

24. Lomsadze A, Ter-Hovhannisyan V, Chernoff YO, et al. Gene identification in novel eukaryotic genomes by self-training algorithm. *Nucleic acids research*. 2005;**33**(20):6494-506.

25. Buchfink B, Xie C, Huson DH. Fast and sensitive protein alignment using DIAMOND. *Nature Methods*. 2015;**12**(1):59.

26. Gotoh O. A space-efficient and accurate method for mapping and aligning cDNA sequences onto genomic sequence. *Nucleic acids research*. 2008;**36**(8):2630-8.

27. Iwata H, Gotoh O. Benchmarking spliced alignment programs including Spaln2, an extended version of Spaln that incorporates additional species-specific features. *Nucleic acids research*.2012;**40**(20):e161-e161.

28. Li H, Handsaker B, Wysoker A, et al. The sequence alignment/map format and SAMtools. *Bioinformatics*. 2009;**25**(16):2078-9.

29. Barnett DW, Garrison EK, Quinlan AR, et al. BamTools: a C++ API and toolkit for analyzing and managing BAM files. *Bioinformatics*. 2011;**27**(12):1691-2.

30. Lomsadze A, Burns PD, Borodovsky M. Integration of mapped RNA-Seq reads into automatic training of eukaryotic gene finding algorithm. *Nucleic acids research*. 2014;**42**(15):e119-e119.

31. Li H. Minimap2: pairwise alignment for nucleotide sequences. *Bioinformatics*. 2018;**34**:3094-100.

32. Grabherr MG, Russell P, Meyer M, et al. Genome-wide synteny through highly sensitive sequence alignment: Satsuma. *Bioinformatics*. 2010;**26**(9):1145-51.

33. Yu Y, Ouyang Y, Yao W. shinyCircos: an R/Shiny application for interactive creation of Circos plot. *Bioinformatics*. 2018;**34**:1229–31.

34. Cabanettes F, Klopp C. D-GENIES: dot plot large genomes in an interactive, efficient and simple way. *PeerJ*. 2018;**6**:e4958

35. Grabherr MG, Haas BJ, Yassour M, et al. Full-length transcriptome assembly from RNA-Seq data without a reference genome. *Nat Biotechnol*. 2011;**29**:644-52.

36. Haas BJ, Papanicolaou A, Yassour M, et al. De novo transcript sequence reconstruction from RNA-seq using the Trinity platform for reference generation and analysis. *Nat Protoc*. 2013;**8**(8): 1494-512.

37. Fu L, Niu B, Zhu Z, et al. CD-HIT: accelerated for clustering the next generation sequencing data. *Bioinformatics*. 2012;**28**(23):3150-2.

38. Emms DM, Kelly S. OrthoFinder: phylogenetic orthology inference for comparative genomics. *Genome Biol*. 2019;**20**:238.

39. Whelan S, Irisarri I, Burki F. PREQUAL: detecting non-homologous characters in sets of unaligned homologous sequences, *Bioinformatics*. 2018;**34**:3929–30.

40. Katoh K, Standley DM. MAFFT multiple sequence alignment software version 7: improvements in performance and usability. *Mol Biol Evol*. 2013;**30**:772–80.

41. Criscuolo A, Gribaldo S. BMGE (Block Mapping and Gathering with Entropy): a new software for selection of phylogenetic informative regions from multiple sequence alignments. *BMC Evol Biol*. 2010;**10**:210.

42. Kalyaanamoorthy S, Minh BQ, Wong TKF, et al. ModelFinder: Fast model selection for accurate phylogenetic estimates. *Nat Methods*, 2017;**14**:587-9.

43. Nguyen LT, Schmidt HA, von Haeseler A, et al. IQ-TREE: A fast and effective stochastic algorithm for estimating maximum likelihood phylogenies. *Mol Biol Evol.* 2015;**32**:268-74.

44. Barghi N, Concepcion GP, Olivera BM, et al. Structural features of conopeptide genes inferred from partial sequences of the *Conus tribblei* genome. *Mol Genet Genom.* 2016;**291**:411–22.

45. Hu H, Bandyopadhyay PK, Olivera BM, et al. Characterization of the *Conus bullatus* genome and its venom-duct transcriptome. *BMC Genomics.* 2011;**12**:60.

46. Andreson R,Roosaare M,Kaplinski L, et al. Gene content of the fish-hunting cone snail *Conus consors*. *bioRxiv* 2019, doi:10.1101/590695

47. Adachi K, Yoshizumi A, Kuramochi T, et al. Novel insights into the evolution of genome size and AT content in mollusks. *Mar Biol* 2021;**168**:25, doi:10.1007/s00227-021-03826-x.

48. Wenger AM, Peluso P, Rowel WJ, et al. Accurate circular consensus long-read sequencing improves variant detection and assembly of a human genome. *Nature Biotech*. 2019;**37**:1155–62.

49. Simone LRL. Phylogeny of the Caenogastropoda (Mollusca), based on comparative morphology. *Arq Zool*. 2011;**42**:161–323.

50. Osca D, Templado J, Zardoya R. Caenogastropod mitogenomics. *Mol Phylogenet Evol.* 2015.;**93**:118-28

51. Takano T, Waré A, Kano Y. Phylogenetic position of the deep-sea snail family Haloceratidae and new insights into caenogastropod relationships. *J Mollusc Stud*, 2022;**88**, doi:10.1093/mollus/eyac012.

52. Hallinan NM, Lindberg DR. Comparative analysis of chromosome counts infers three paleopolyploidies in the Mollusca. *Genome Biol Evol* . 2011;**3**:1150–63.

53. Liu C, Zhang Y, Ren Y, et al. The genome of the golden apple snail *Pomacea canaliculata* provides insight into stress tolerance and invasive adaptation. *Gigascience*. 2018;**7**:1–13, doi:10.1093/gigascience/giy101.

54. Lemarcis T, Fedosov AE, Kantor YI, et al. Neogastropod (Mollusca, Gastropoda) phylogeny: a step forward with mitogenomes. *Zool Scri*, 2022.;**51**:550-61, doi:10.1111/zsc.12552.

55. Abalde S, Tenorio MJ, Uribe JE, et al. Conidae phylogenomics and evolution. *Zool* Scr. 2019;**48**:194– 214, doi:10.1111/zsc.12329.

56. Herraez-Perez A, Pardos-Blas JR, Afonso CML, et, al. Supporting data for "Chromosome-Level Genome of the Venomous Snail Kalloconus canariensis: A Valuable Model for Venomics and Comparative Genomics" GigaScience Database. 2023. http://dx.doi.org/10.5524/102421

Table 1: Assembly statistics and annotation parameters of *K. canariensis* genome. All metrics are based on contigs of size ≥ 500 bp.

**Contig Assembly**					
	Number of Reads	31,761,787 (371.2 Gb)			
	Estimated genome size	3.6 Gb			
	Total length (bp)	2,867,696,795			
	Number of Contigs	25,961			
	Longest contig (bp)	4,977,278			
	N50 (bp)	646,466			
	N90 (bp)	59,506			
	GC (%)	43.84			
	BUSCOv5.1.3	(metazoa_odb10)	n=954		
	Complete	93.10%	888		
	Complete single copy	87.20%	832		
	Complete duplicated	5.90%	56		
	Fragmented	4.20%	40		
	Missing	2.70%	26		
**Scaffold Assembly**					
	Total length (bp)	2,868,185,268			
	Total No. Scaffolds	18,573			
	Scaffolds (>= 1000 bp)	18,495			
	Largest Scaffold	153,129,599			
	N50 (bp)	79,645,777			
	N90 (bp)	40,485,963			
	GC (%)	43.84			
	BUSCOv5.1.3	(metazoa_odb10)	n=954		
	Complete	93.50%	892		
	Complete single copy	87.50%	935		
	Complete duplicated	6%	57		
	Fragmented	3.80%	36		
	Missing	2.70%	26		
**Genome Annotation**					
Repeats Masked	Total genome masked	49.55%			
	Class I TEs repeats	18.56%			
	Class II TEs repeats	7.58%			
	Low complexity repeats	0.97%			
	Simple repeats	10.51%			
		**MAKER**		**BRAKER**	
Gene Prediction	Total number of genes	34,250		34,287	
	Total coding region (bp)	37,756,940 (37.756 Mb)		50,351,333 (50.351 Mb)	
	Number of single-exon genes	10,549		-	
Protein assessment	BUSCOv5.1.3	(metazoa_odb10)	n=954	(metazoa_odb10)	n=954
	Complete	81.60%	779	90.90%	867
	Complete single copy	80.90%	772	82.30%	785

	Complete duplicated	0.70%	7	8.60%	82
	Fragmented	5.10%	49	6.10%	58
	Missing	13.30%	126	3.00%	29

Table 1 Click here to access/download;Table;Table 1.xlsx

Table 1: Assembly statistics and annotation parameters of *K. canariensis* genome. All metrics are based on contigs of size≥500

**Contig Assembly**					
	Number of Reads	31,761,787 (371.2 Gb)			
	Estimated genome size	3.6 Gb			
	Total length (bp)	2,867,696,795			
	Number of Contigs	25,961			
	Longest contig (bp)	4,977,278			
	N50 (bp)	646,466			
	N90 (bp)	59,506			
	GC (%)	43.84			
	BUSCOv5.1.3	(metazoa_odb10)	n=954		
	Complete	93.10%	888		
	Complete single copy	87.20%	832		
	Complete duplicated	5.90%	56		
	Fragmented	4.20%	40		
	Missing	2.70%	26		
**Scaffold Assembly**					
	Total length (bp)	2,868,185,268			
	Total No. Scaffolds	18,573			
	Scaffolds (>= 1000 bp)	18,495			
	Largest Scaffold	153,129,599			
	N50 (bp)	79,645,777			
	N90 (bp)	40,485,963			
	GC (%)	43.84			
	BUSCOv5.1.3	(metazoa_odb10)	n=954		
	Complete	93.50%	892		
	Complete single copy	87.50%	935		
	Complete duplicated	6%	57		
	Fragmented	3.80%	36		
	Missing	2.70%	26		
**Genome Annotation**					
Repeats Masked	Total genome masked	49.55%			
	Class I TEs repeats	18.56%			
	Class II TEs repeats	7.58%			
	Low complexity repeats	0.97%			
	Simple repeats	10.51%			
		**MAKER**		**BRAKER**	
Gene Prediction	Total number of genes	34,250		34,287	
	Total coding region (bp)	37,756,940 (37.756 Mb)		50,351,333 (50.351 Mb)	
	Number of single-exon genes	10,549		-	
Protein assessment	BUSCOv5.1.3	(metazoa_odb10)	n=954	(metazoa_odb10)	n=954
	Complete	81.60%	779	90.90%	867
	Complete single copy	80.90%	772	82.30%	785
	Complete duplicated	0.70%	7	8.60%	82

	Fragmented	5.10%	49	6.10%	58
	Missing	13.30%	126	3.00%	29

C:90.9%[S:82.3%,D:8.6%],F:6.1%,M:3.0%,n:954

867	Complete BUSCOs (C)
785	Complete and single-copy BUSCOs (S)

82	Complete and duplicated BUSCOs (D)
58	Fragmented BUSCOs (F)
29	Missing BUSCOs (M)

Figure 1

Click here to access/download;Figure;Fig.1.pdf

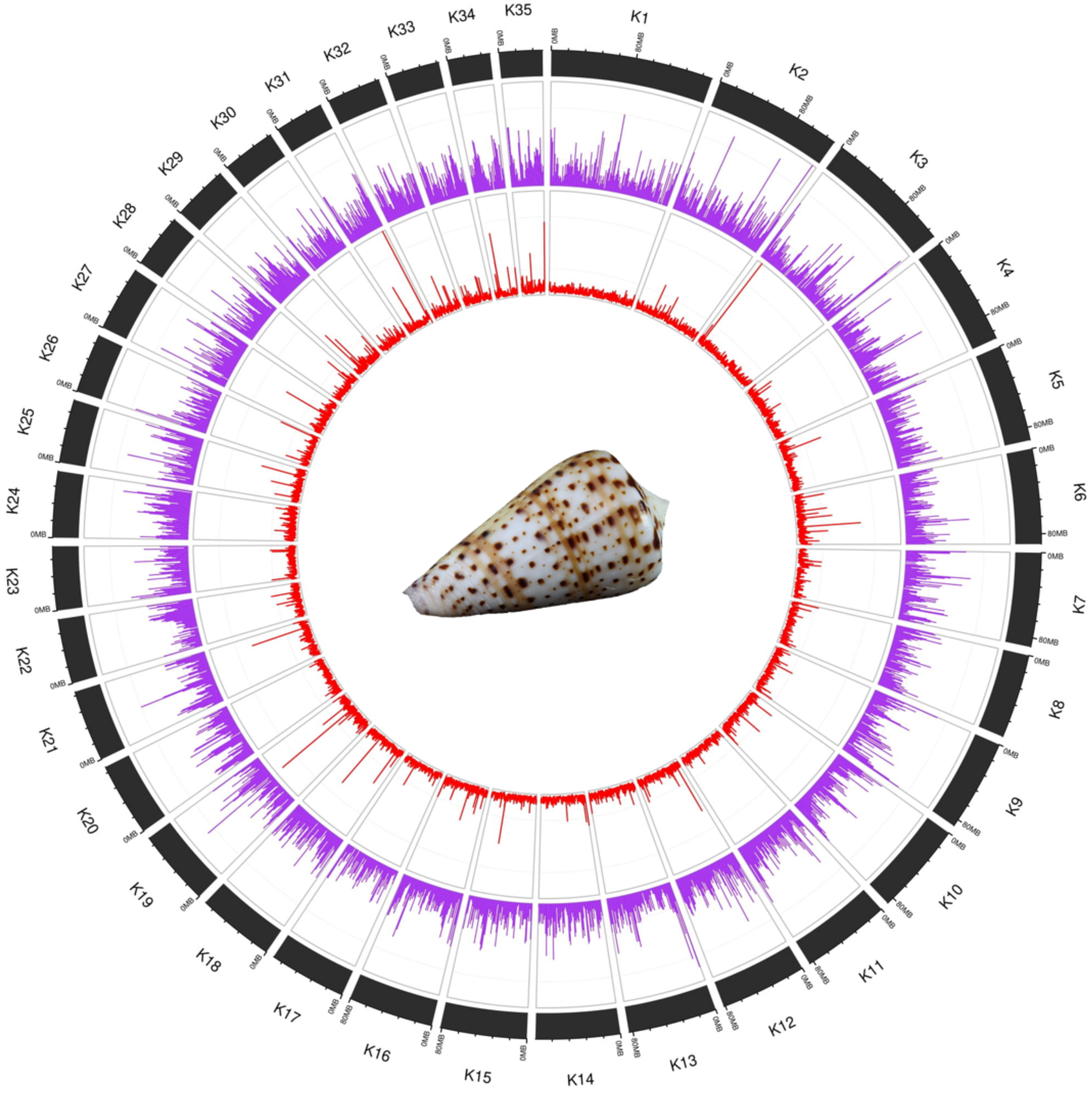

Figure 2

Click here to access/download;Figure;Fig.2.pdf

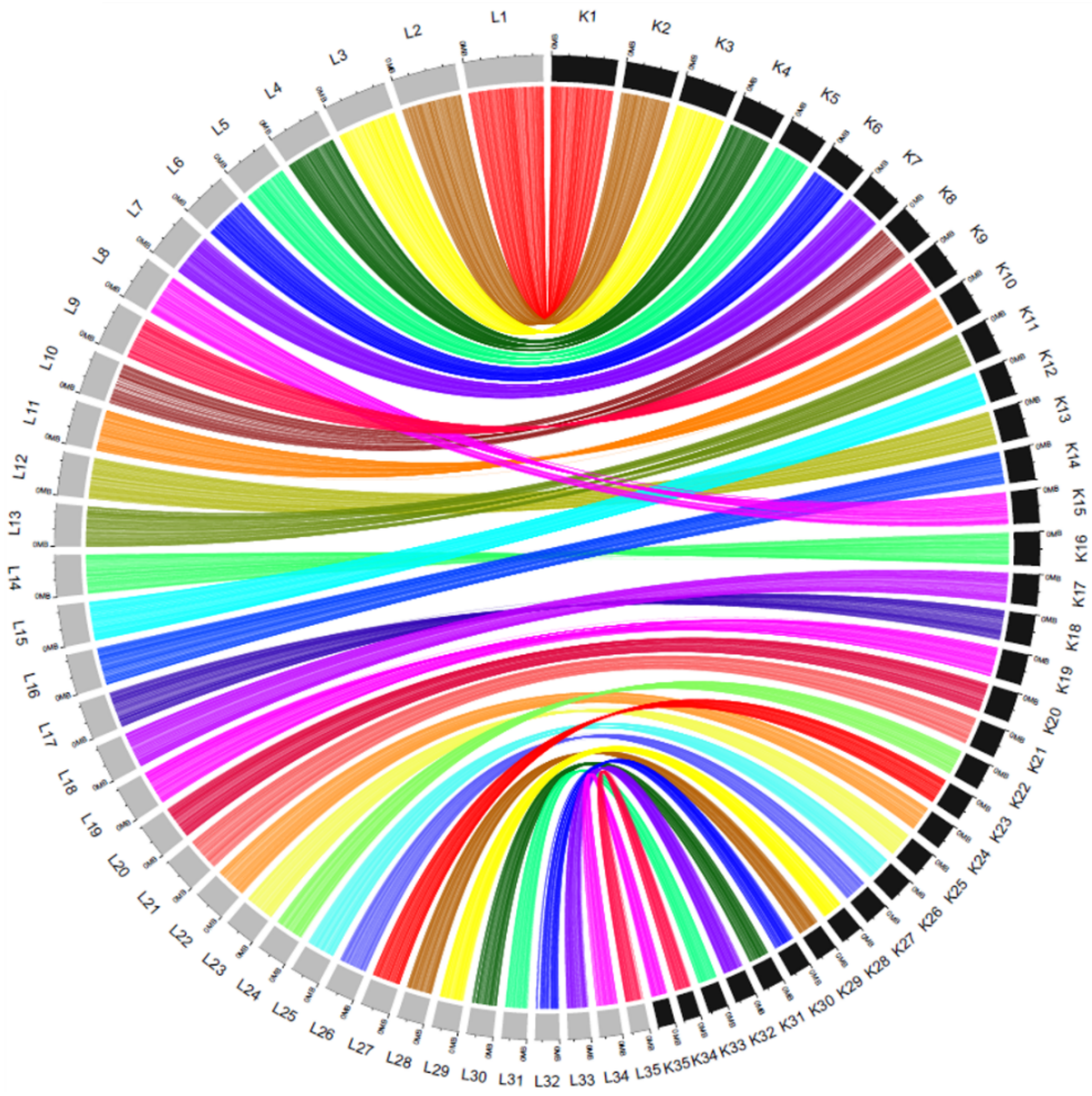

Click here to access/download
Supplementary Material
Supp_Mat.pdf

Personal Cover Click here to access/download;Personal Cover;Cover2.pdf

MINISTERIO DE
ECONOMÍA, INDUSTRIA Y
COMPETITIVIDAD

GigScience
Editor in Chief

Madrid, July 4th, 2023

Dear Editor,

Please find uploaded an electronic copy of the revised version of our paper entitled "Chromosome-Level Genome of the Venomous Snail Kalloconus canariensis: A Valuable Model for Venomics and Comparative Genomics" for publication in *GigaScience*.
We were pleased to find out that the two reviewers liked the paper. Upon your request, we have incorporated all suggestions by the reviewers into this revised version. Please find in the "Response to reviewers" document detailed explanation on the changes that were made in the manuscript. In brief, we have better explained how RNA seq data was used in genome annotation, the assembly of transcriptomes for phylogenomic reconstruction was repeated using Trinity, and genome annotation was redone using Augustus v3.5 and Braker2 v2.1.6. We believe that the paper has improved significantly with reviewers feedback and that the results of our study will be of interest to the broader readership of *GigaScience*.

Thank you very much for your prompt handling of the manuscript.

Yours sincerely,

Rafael Zardoya

Rafael Zardoya

rafaz@mncn.csic.es

C/ JOSÉ GUTIERREZ ABASCAL,2
28006 MADRID ESPAÑA
TEL:91.411.13.28
Ext. 1129
FAX: 915645078
