# Supplementary material for: Chromosome-level genome of the venomous snail Kalloconus canariensis: a valuable model for venomics and comparative genomics
Source: Gigascience. 2023 Sep 30;12:giad075. doi: 10.1093/gigascience/giad075 (PMC10541794; doi:10.1093/gigascience/giad075)
Supplement: giad075_Supplemental_File [file giad075_supplemental_file.pdf]

Fig. S1. Link density histogram (35 pseudo-chromosomes)

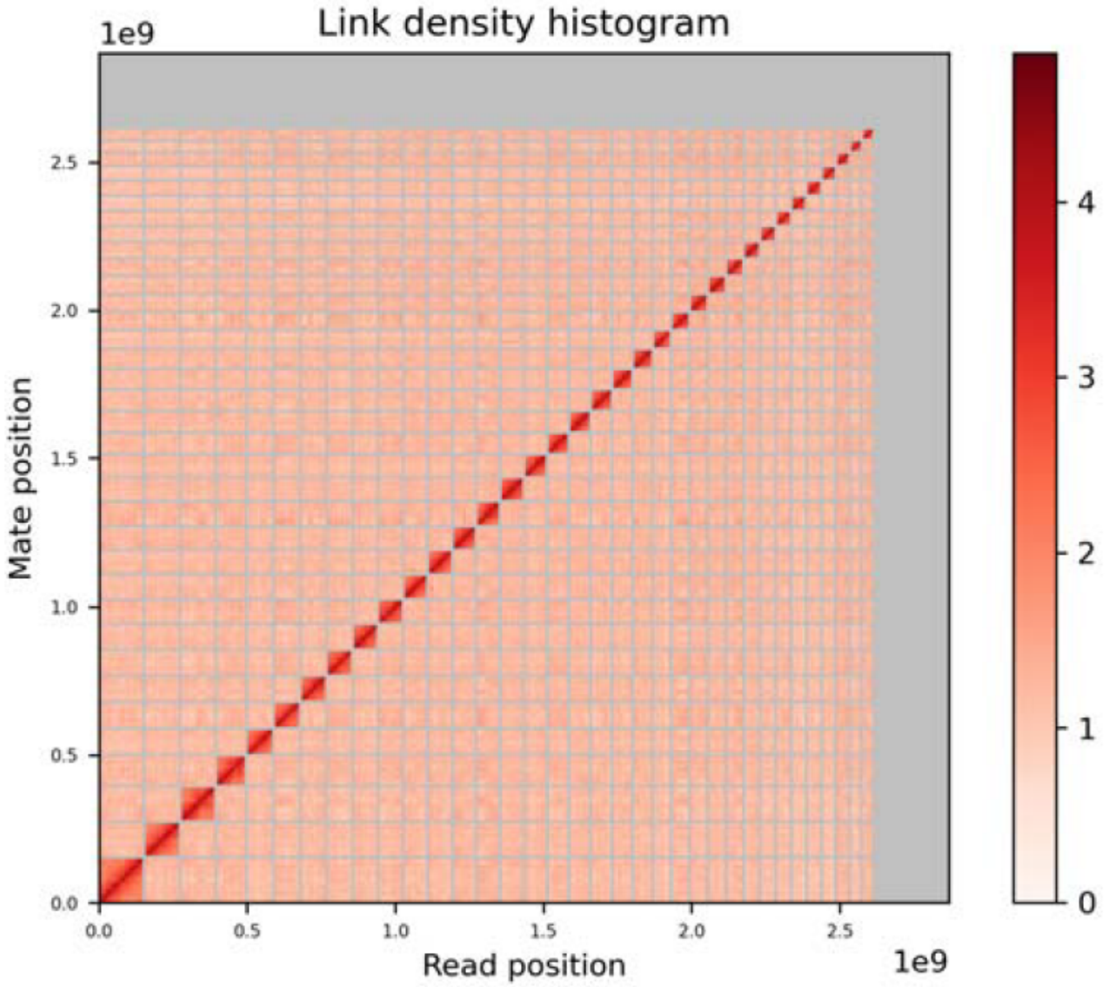

Fig. S2. Differences in gene and intergenic regions lengths between *L. ventricosus* (left blue, Lven) and *K. cana*riensis (right orange, Kcan).

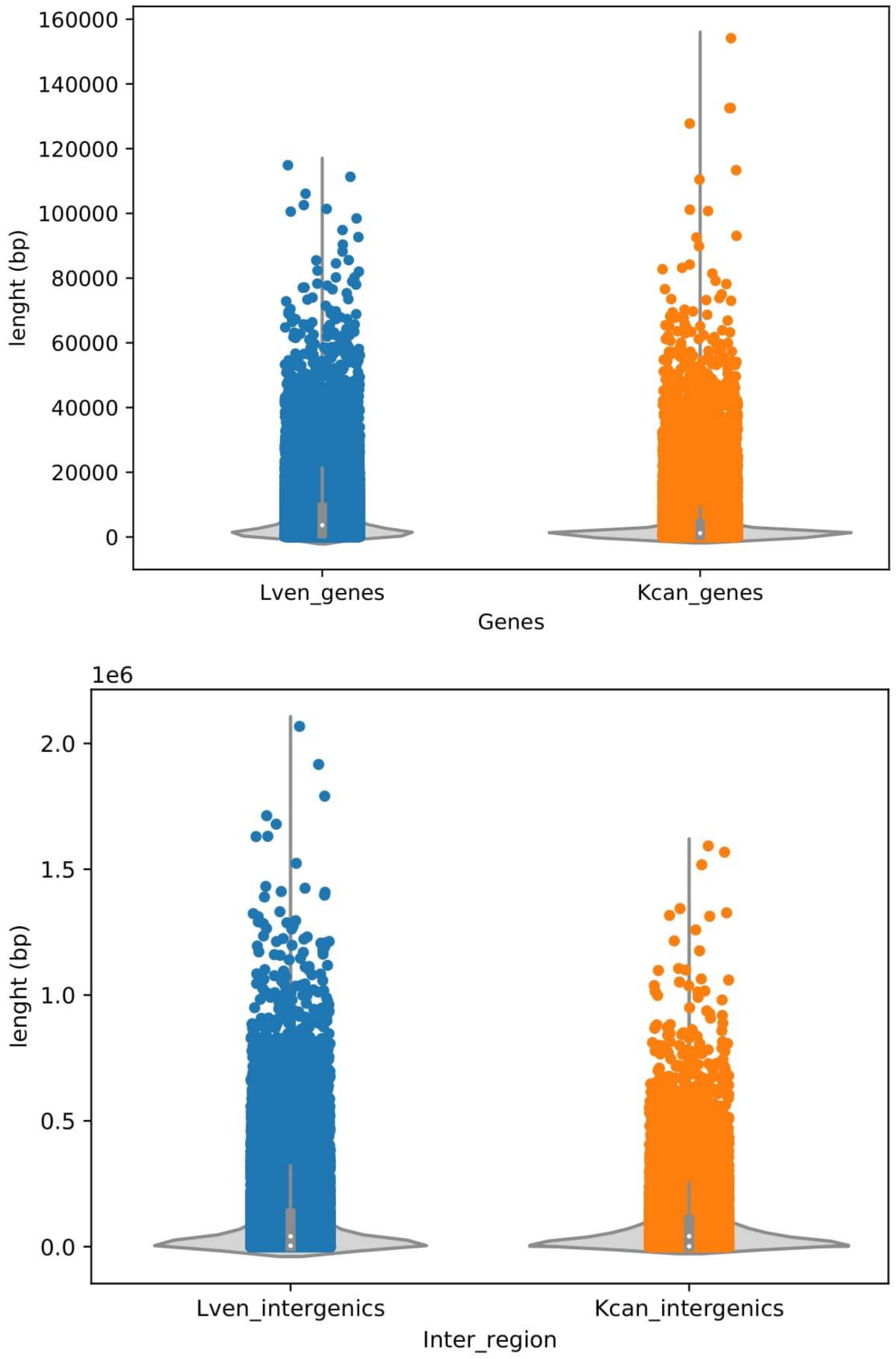

Fig. S3. Synteny maps between *L. ventricosus* (L1 to L35) and *K. canariensis* (K1 to K35) pseudo-chromosomes.

K1 – L1

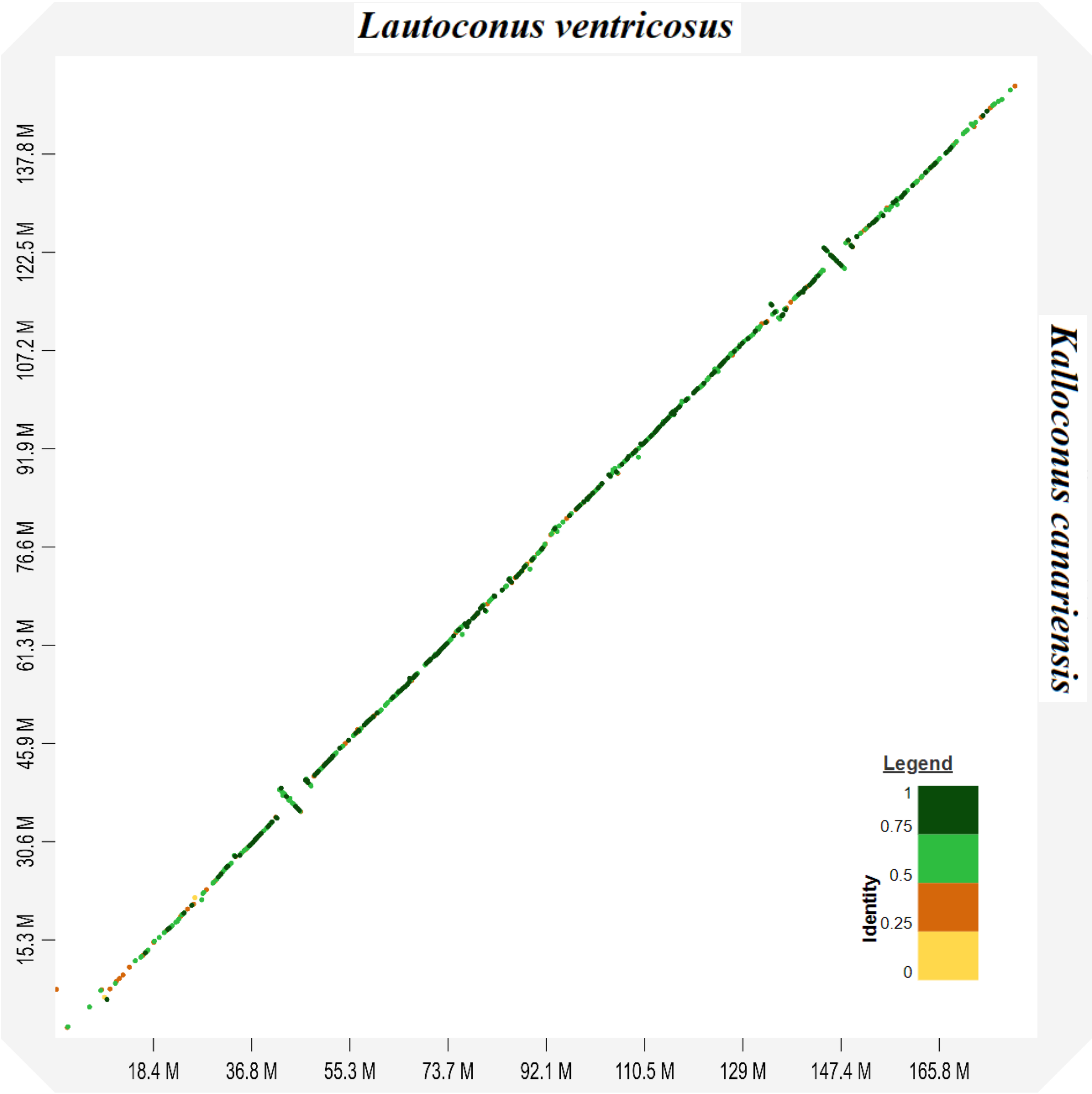

K2 – L2

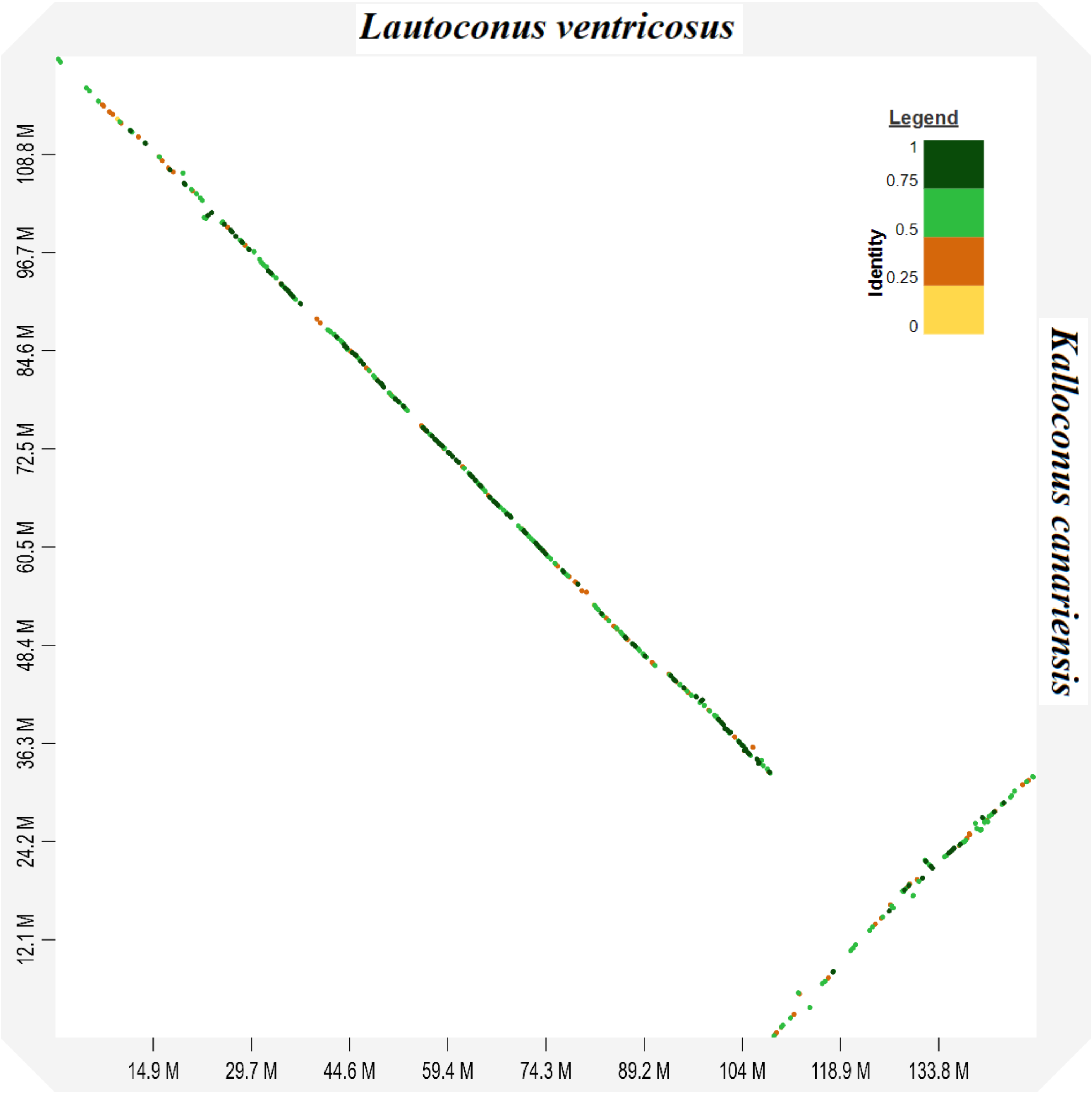

Lautoconus ventricosus
Kalloconus canariensis
Legend
Identity
1
0.75
0.5
0.25
0
108.8 M
96.7 M
84.6 M
72.5 M
60.5 M
48.4 M
36.3 M
24.2 M
12.1 M
14.9 M
29.7 M
44.6 M
59.4 M
74.3 M
89.2 M
104 M
118.9 M
133.8 M

K3 – L3

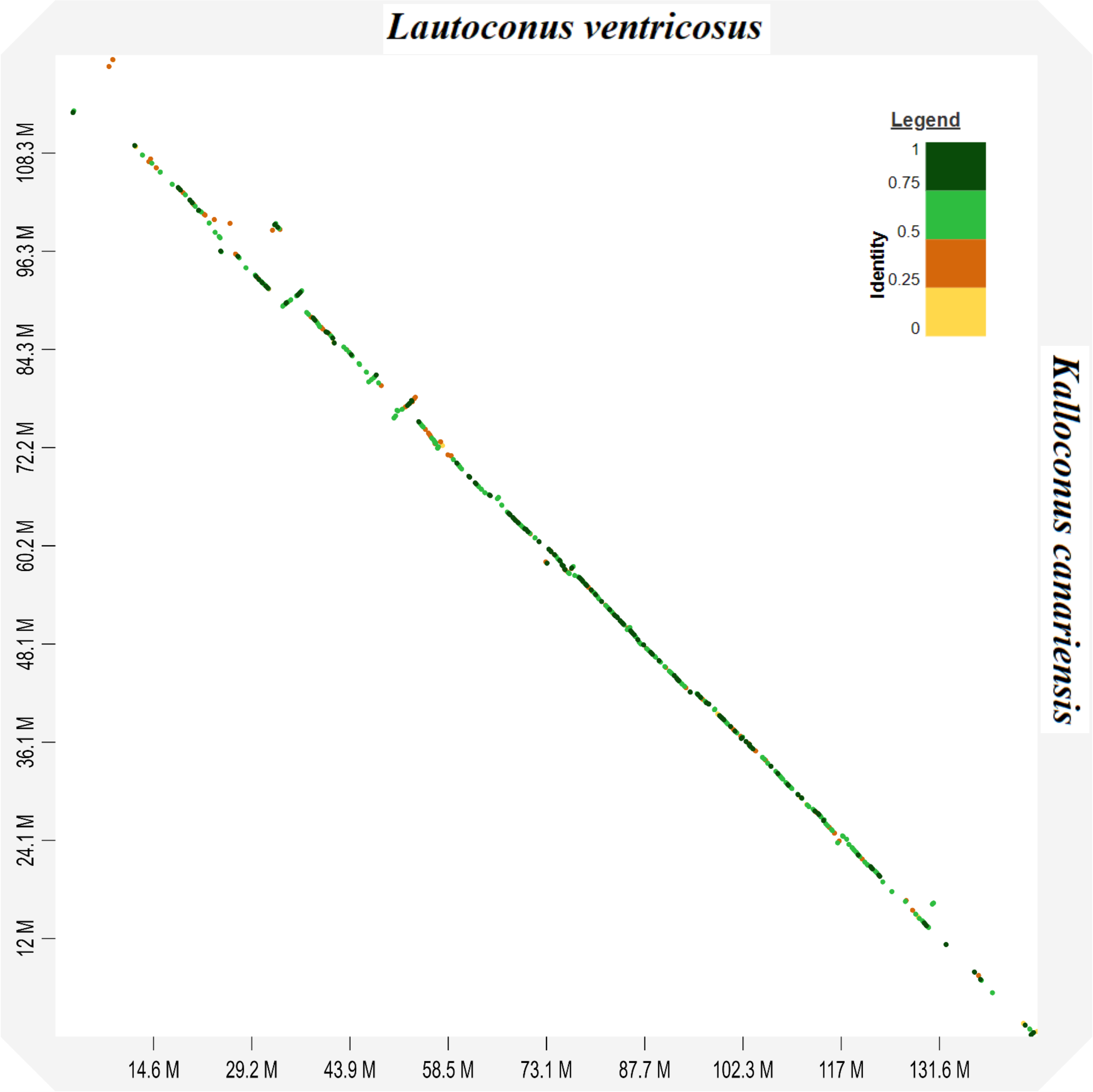

Lautoconus ventricosus
Kalloconus canariensis
Legend
Identity
1
0.75
0.5
0.25
0
108.3 M
96.3 M
84.3 M
72.2 M
60.2 M
48.1 M
36.1 M
24.1 M
12 M
14.6 M
29.2 M
43.9 M
58.5 M
73.1 M
87.7 M
102.3 M
117 M
131.6 M

K4 - L4

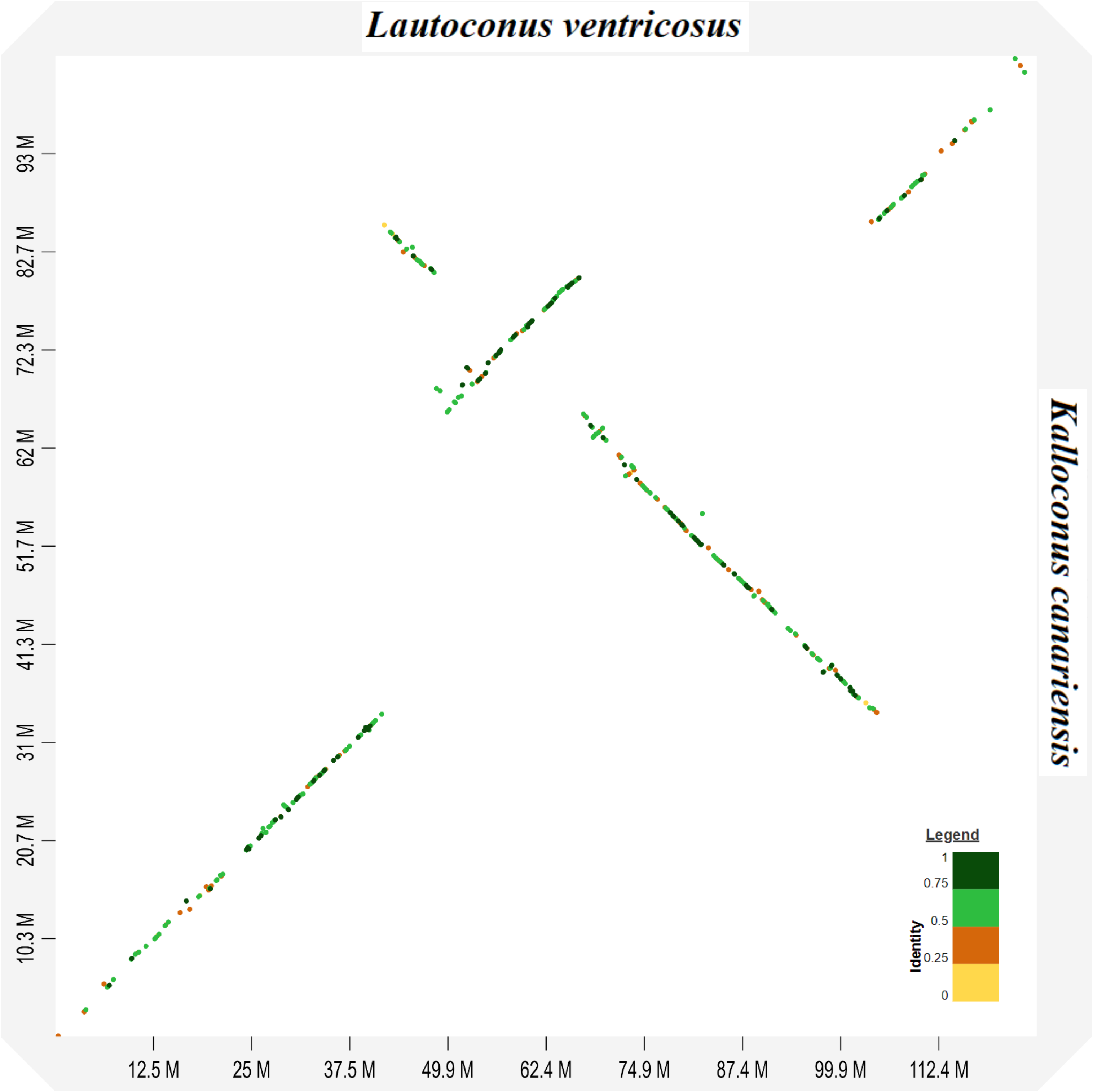
Lautoconus ventricosus
Kalloconus canariensis
93 M
82.7 M
72.3 M
62 M
51.7 M
41.3 M
31 M
20.7 M
10.3 M
12.5 M
25 M
37.5 M
49.9 M
62.4 M
74.9 M
87.4 M
99.9 M
112.4 M
Legend
Identity
1
0.75
0.5
0.25
0

K5 – L5

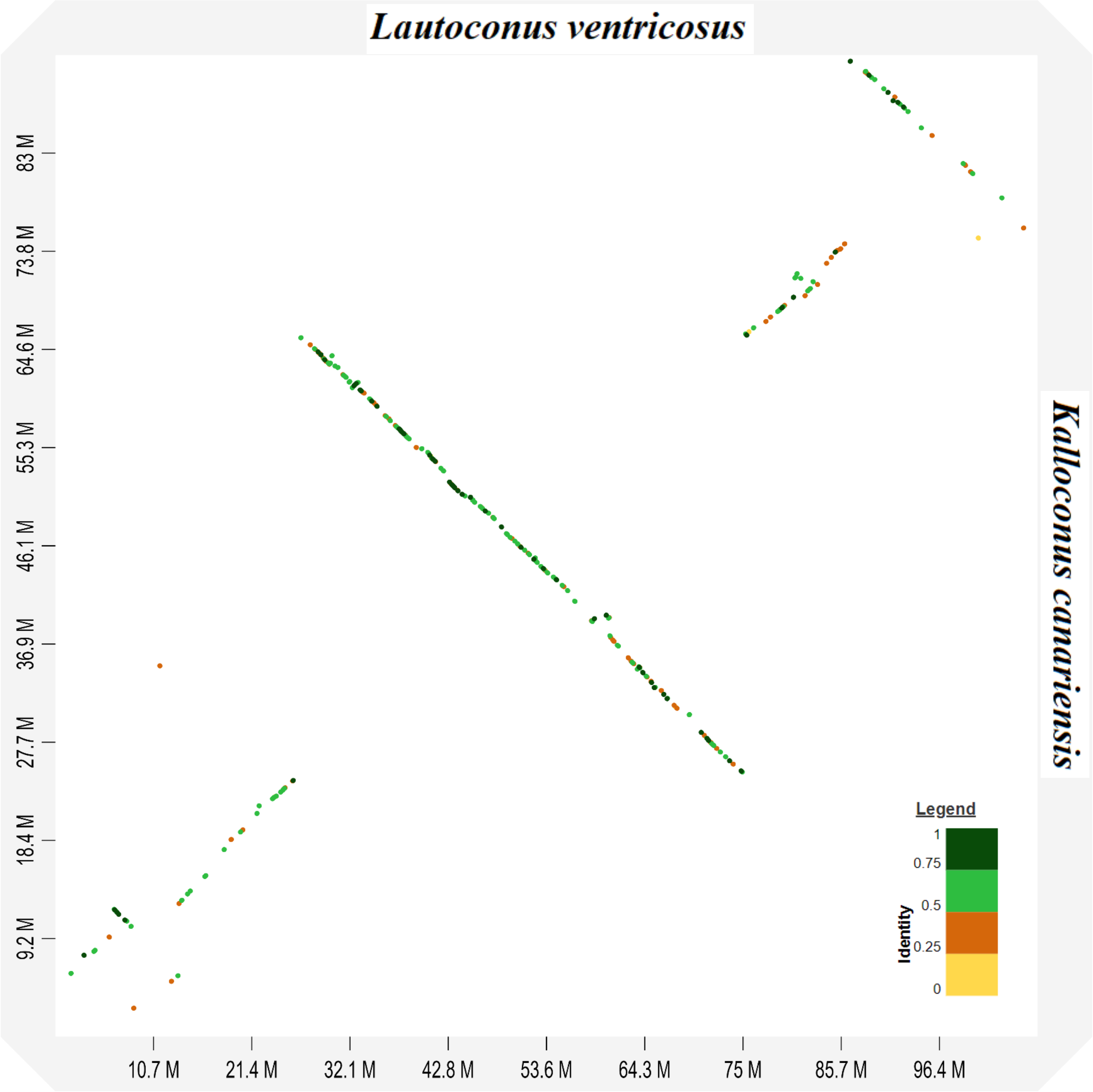
Lautoconus ventricosus
Kalloconus canariensis
83 M
73.8 M
64.6 M
55.3 M
46.1 M
36.9 M
27.7 M
18.4 M
9.2 M
10.7 M
21.4 M
32.1 M
42.8 M
53.6 M
64.3 M
75 M
85.7 M
96.4 M
Legend
Identity
1
0.75
0.5
0.25
0

K6 – L6

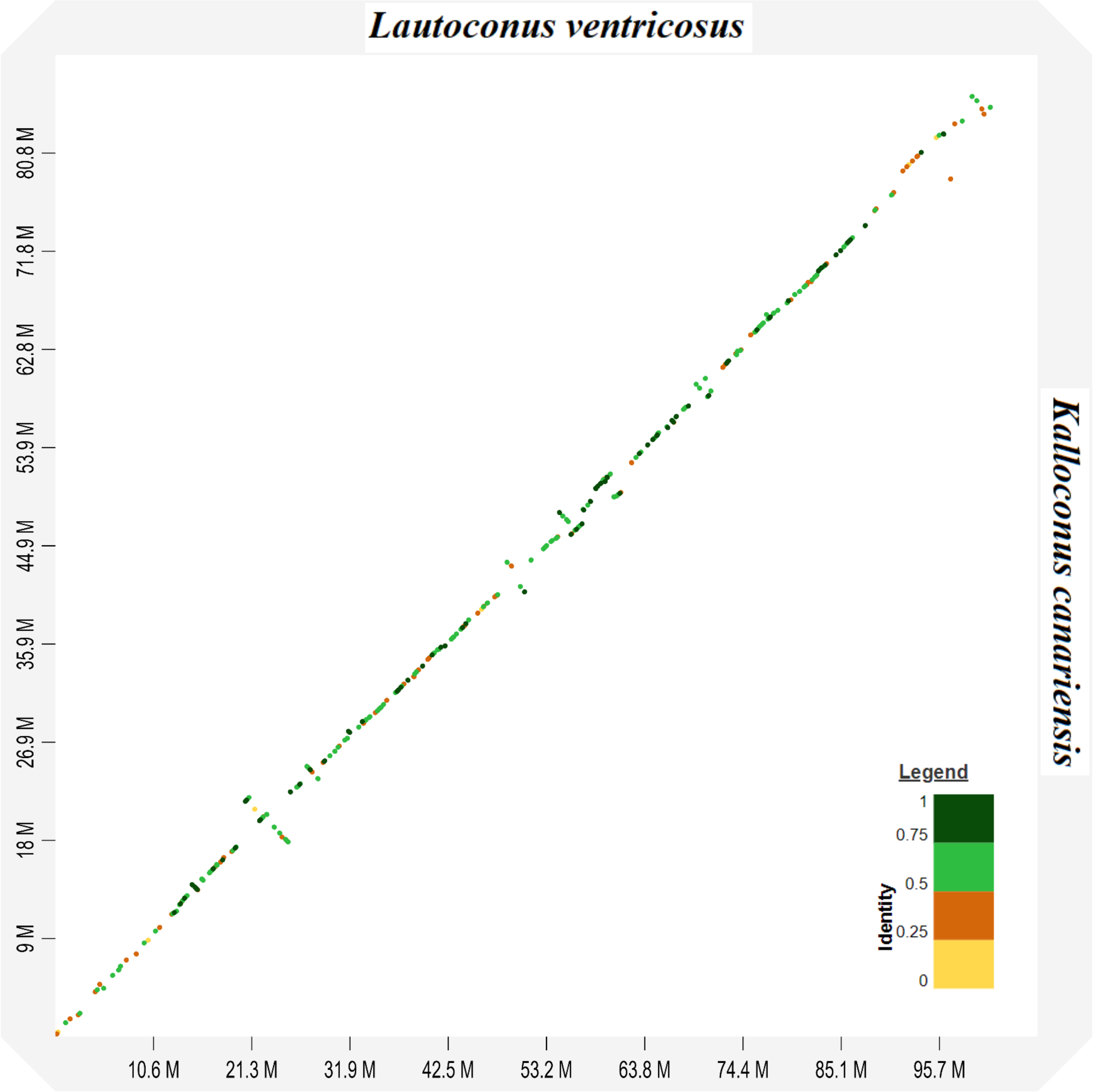

Lautoconus ventricosus
Kalloconus canariensis
80.8 M
71.8 M
62.8 M
53.9 M
44.9 M
35.9 M
26.9 M
18 M
9 M
10.6 M
21.3 M
31.9 M
42.5 M
53.2 M
63.8 M
74.4 M
85.1 M
95.7 M
Legend
Identity
1
0.75
0.5
0.25
0

K7 – L7

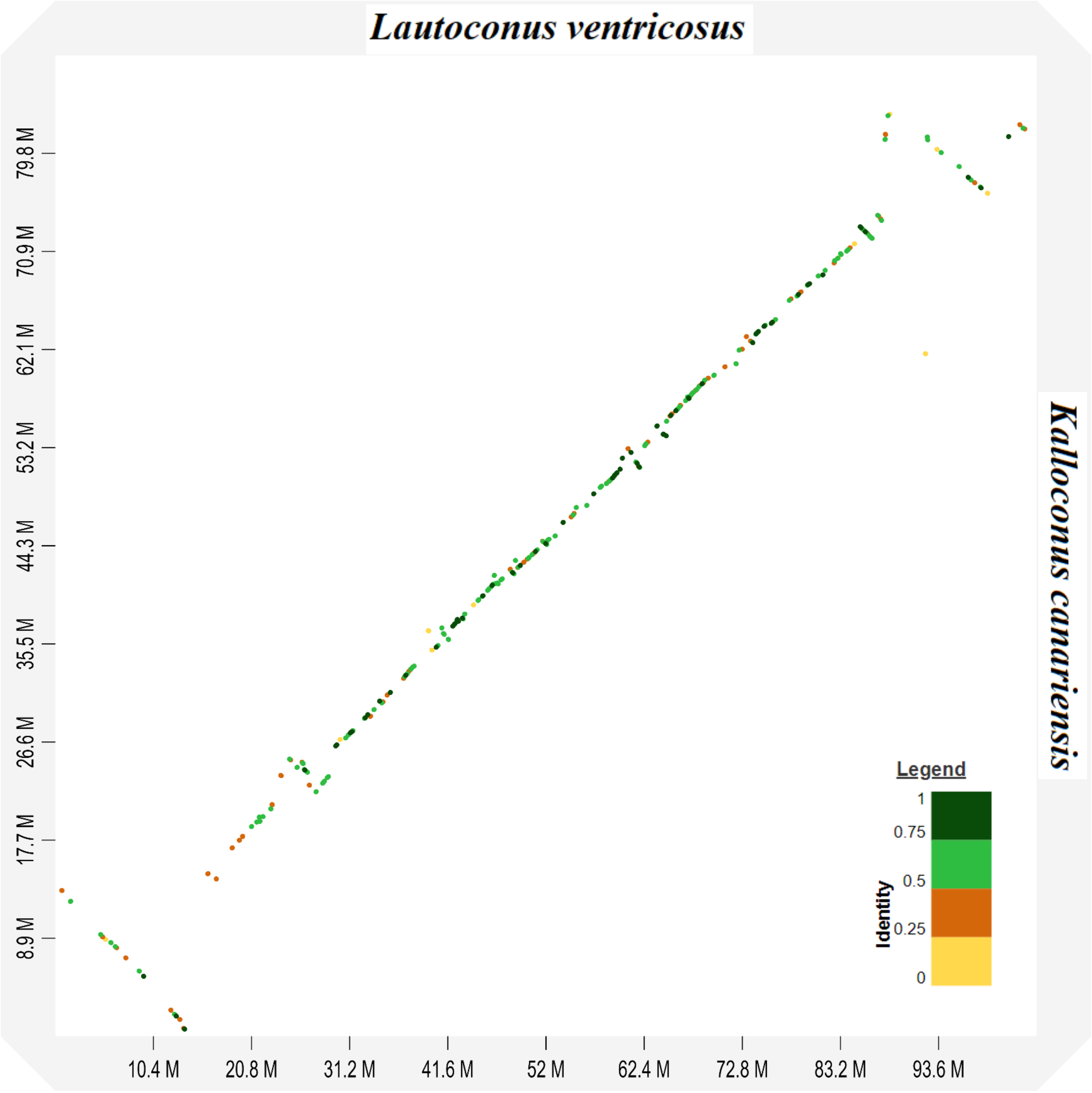

Lautoconus ventricosus
Kalloconus canariensis
79.8 M
70.9 M
62.1 M
53.2 M
44.3 M
35.5 M
26.6 M
17.7 M
8.9 M
10.4 M
20.8 M
31.2 M
41.6 M
52 M
62.4 M
72.8 M
83.2 M
93.6 M
Legend
Identity
1
0.75
0.5
0.25
0

K8 - L10

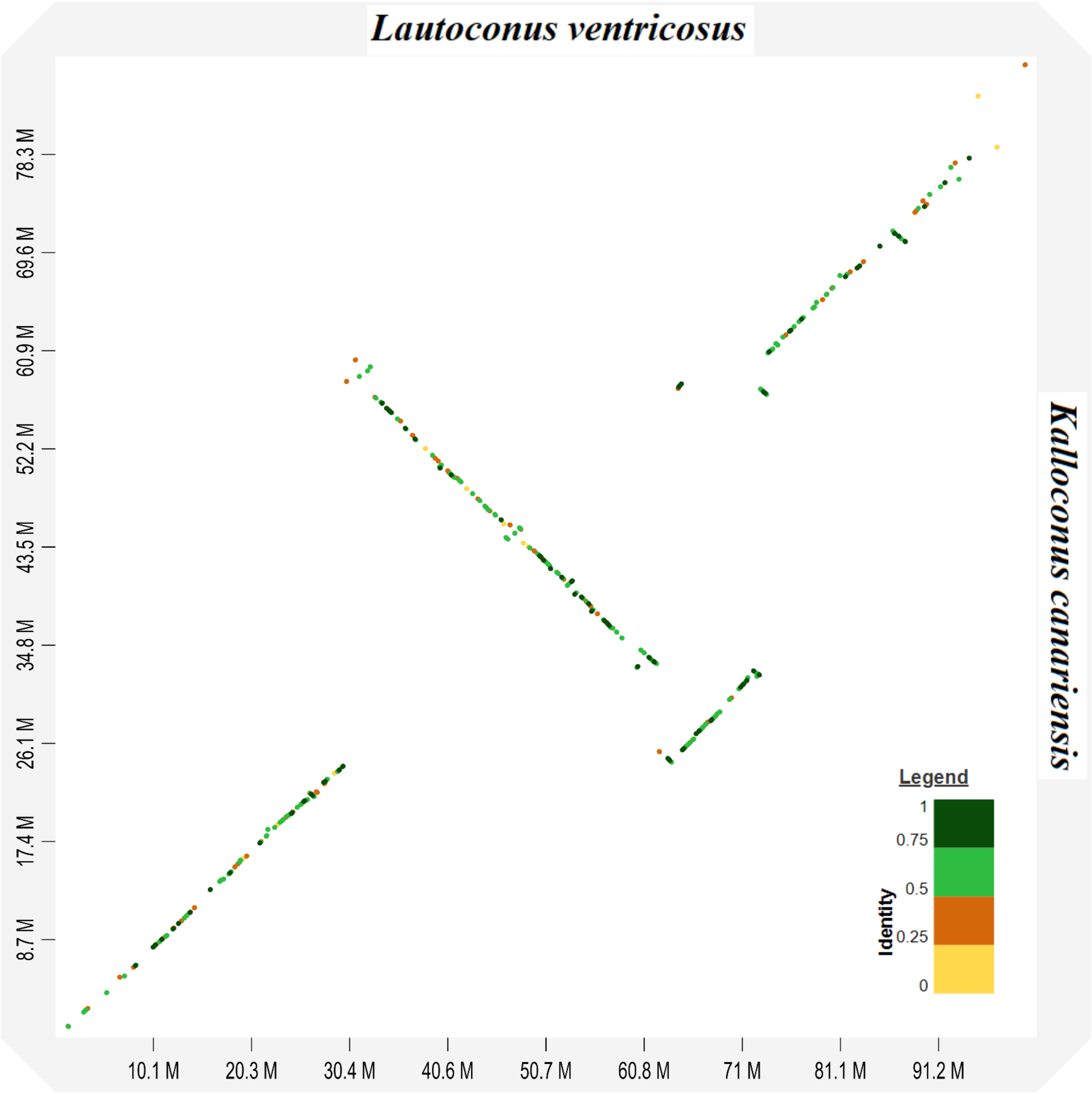

Lautoconus ventricosus
Kalloconus canariensis
78.3 M
69.6 M
60.9 M
52.2 M
43.5 M
34.8 M
26.1 M
17.4 M
8.7 M
10.1 M
20.3 M
30.4 M
40.6 M
50.7 M
60.8 M
71 M
81.1 M
91.2 M
Legend
Identity
1
0.75
0.5
0.25
0

K9 – L9

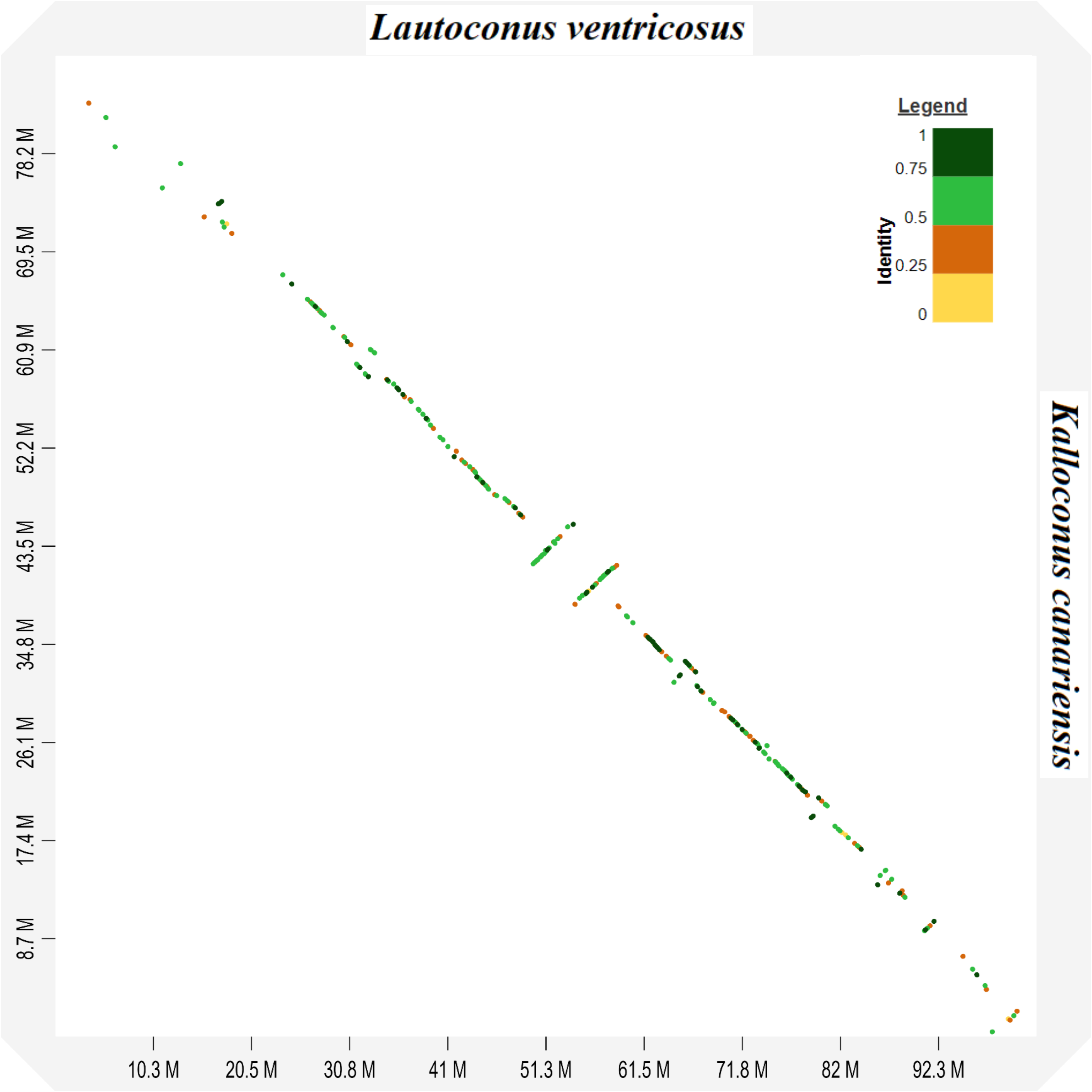
Lautoconus ventricosus
Kalloconus canariensis
Legend
Identity
1
0.75
0.5
0.25
0
78.2 M
69.5 M
60.9 M
52.2 M
43.5 M
34.8 M
26.1 M
17.4 M
8.7 M
10.3 M
20.5 M
30.8 M
41 M
51.3 M
61.5 M
71.8 M
82 M
92.3 M

K10 - L11

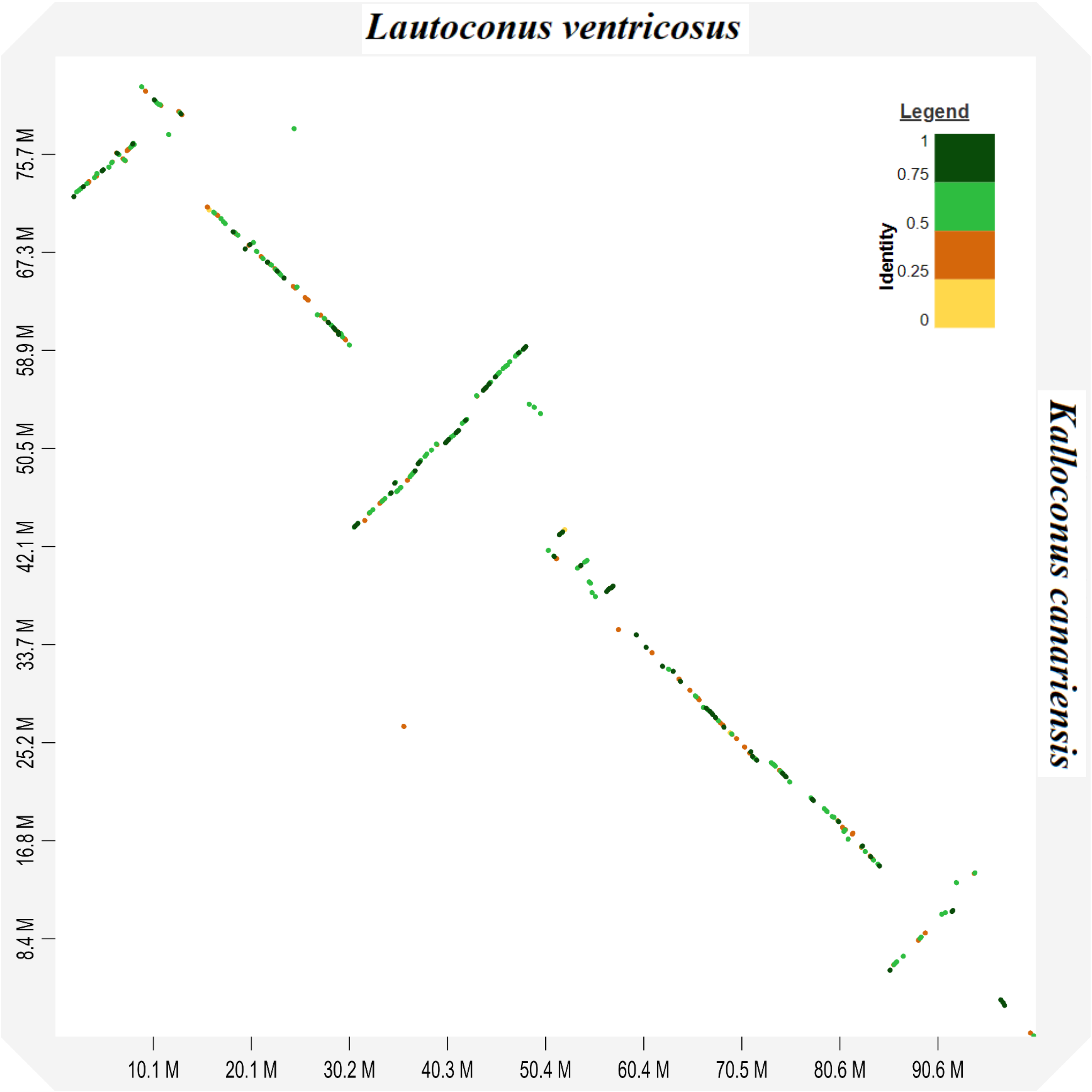

Lautoconus ventricosus
Kalloconus canariensis
Legend
Identity
1
0.75
0.5
0.25
0
75.7 M
67.3 M
58.9 M
50.5 M
42.1 M
33.7 M
25.2 M
16.8 M
8.4 M
10.1 M
20.1 M
30.2 M
40.3 M
50.4 M
60.4 M
70.5 M
80.6 M
90.6 M

K11 - L13

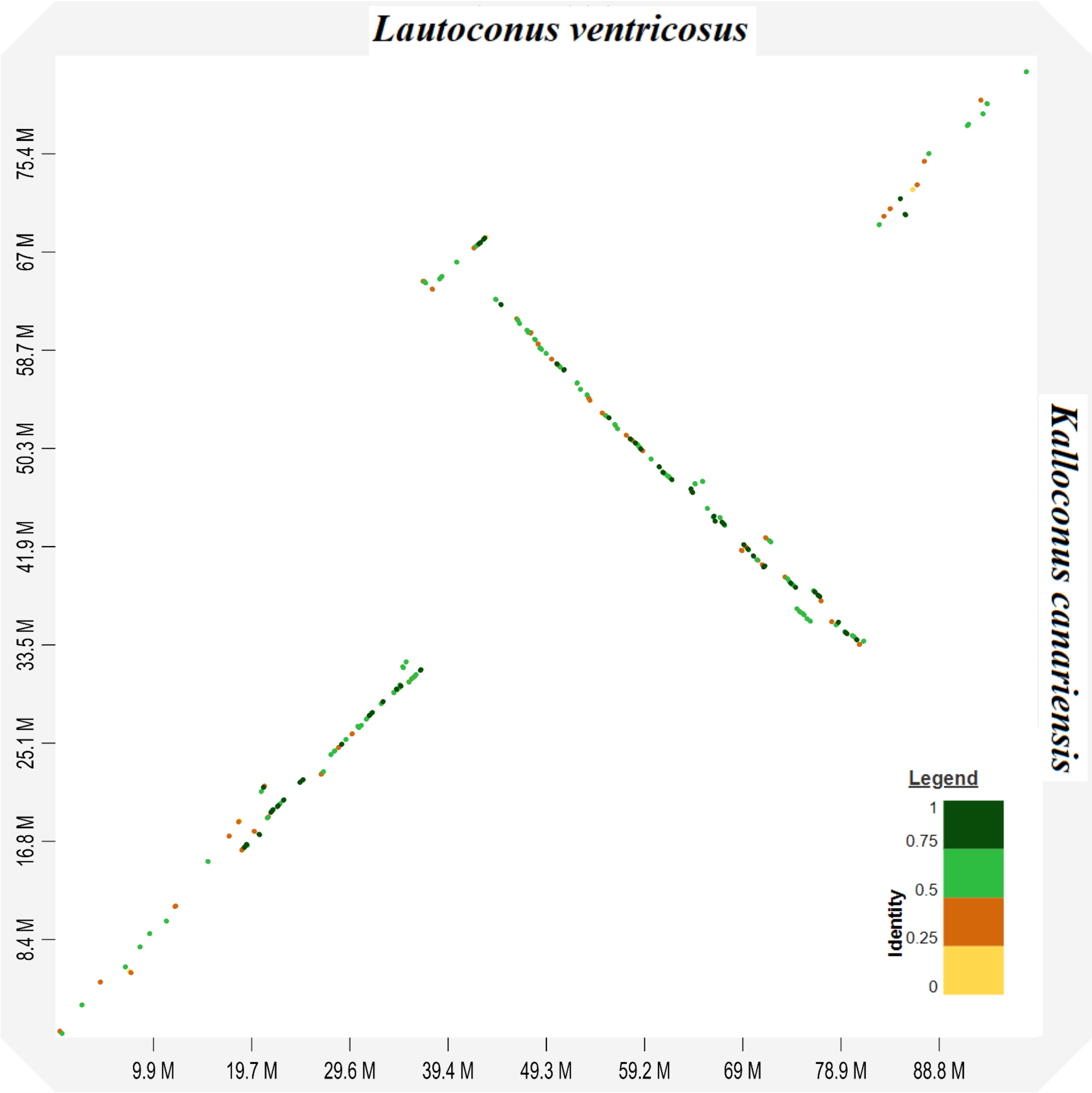

Lautoconus ventricosus
Kalloconus canariensis
75.4 M
67 M
58.7 M
50.3 M
41.9 M
33.5 M
25.1 M
16.8 M
8.4 M
9.9 M
19.7 M
29.6 M
39.4 M
49.3 M
59.2 M
69 M
78.9 M
88.8 M
Legend
Identity
1
0.75
0.5
0.25
0

K12 - L15

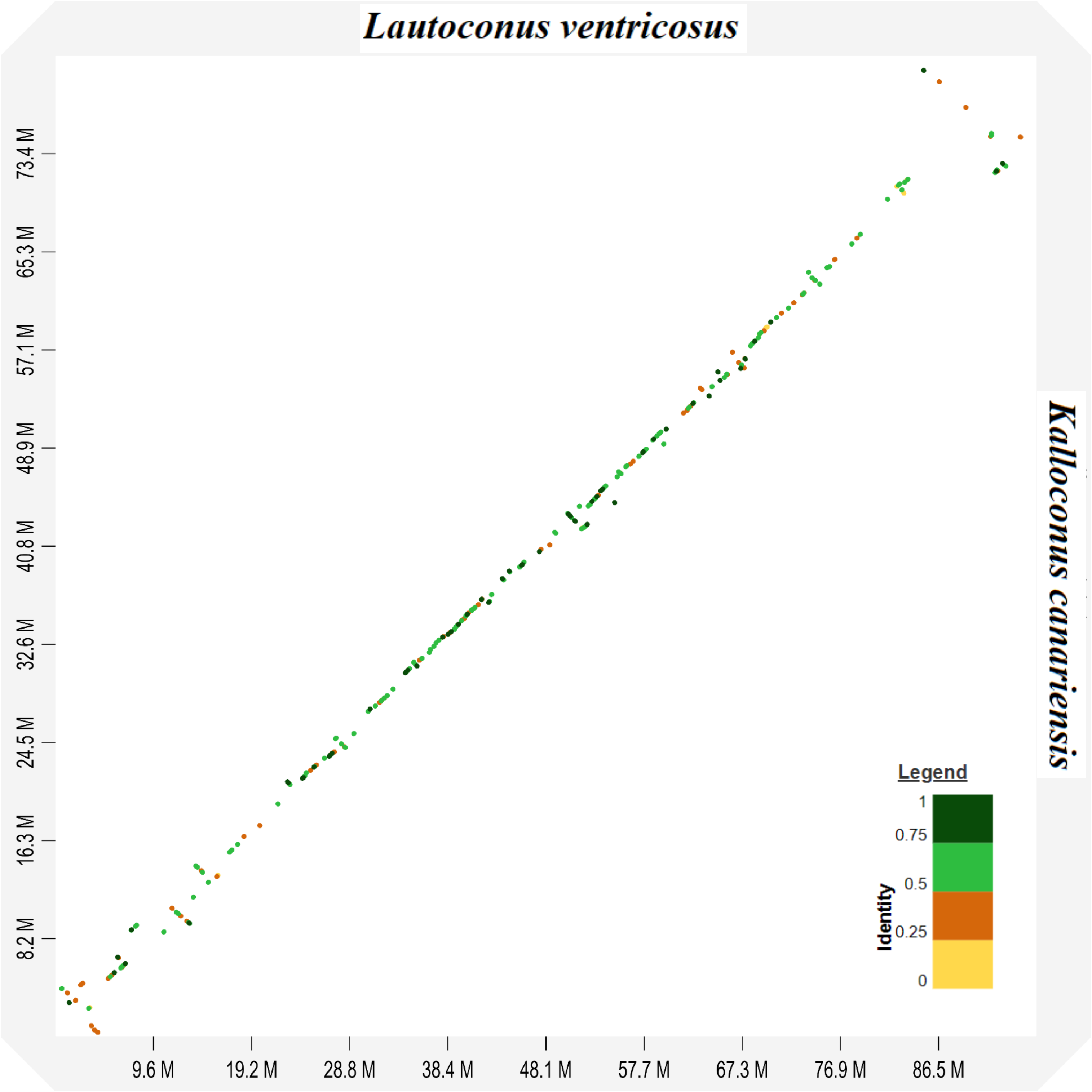

Lautoconus ventricosus
Kalloconus canariensis
73.4 M
65.3 M
57.1 M
48.9 M
40.8 M
32.6 M
24.5 M
16.3 M
8.2 M
9.6 M
19.2 M
28.8 M
38.4 M
48.1 M
57.7 M
67.3 M
76.9 M
86.5 M
Legend
Identity
1
0.75
0.5
0.25
0

K13 -L12

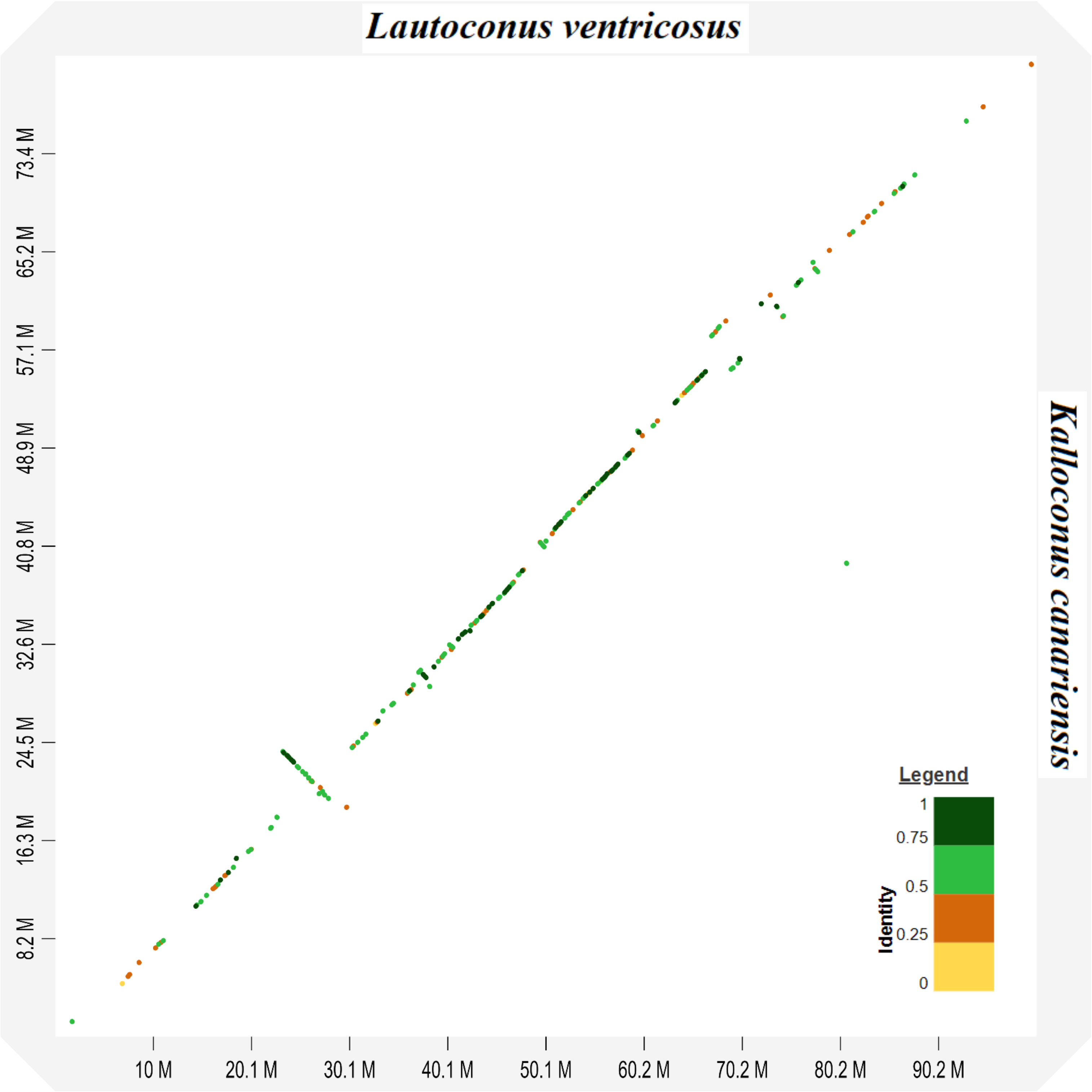

Lautoconus ventricosus
Kalloconus canariensis
73.4 M
65.2 M
57.1 M
48.9 M
40.8 M
32.6 M
24.5 M
16.3 M
8.2 M
10 M
20.1 M
30.1 M
40.1 M
50.1 M
60.2 M
70.2 M
80.2 M
90.2 M
Legend
Identity
1
0.75
0.5
0.25
0

K14 - L16

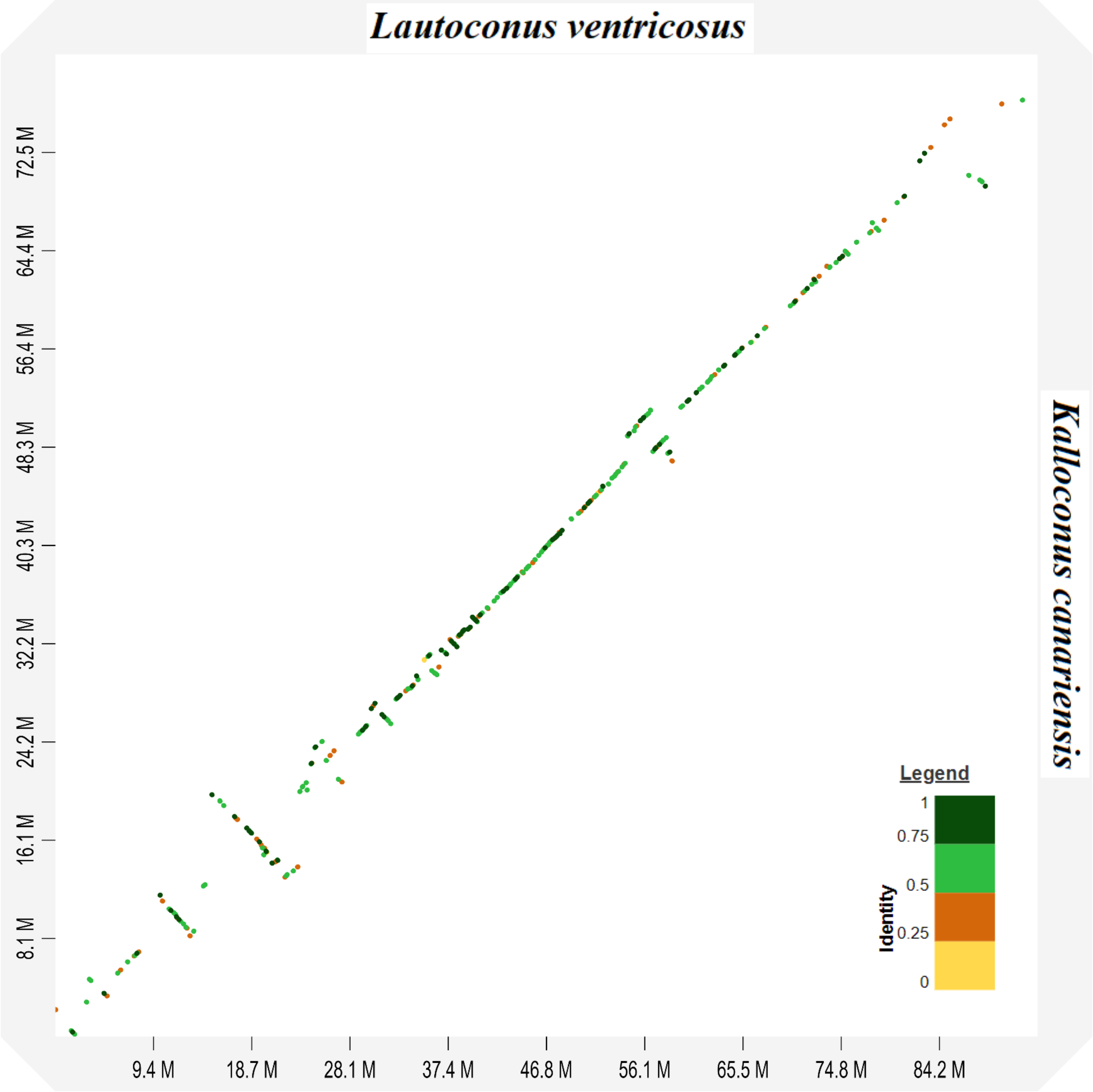

Lautoconus ventricosus
Kalloconus canariensis
72.5 M
64.4 M
56.4 M
48.3 M
40.3 M
32.2 M
24.2 M
16.1 M
8.1 M
9.4 M
18.7 M
28.1 M
37.4 M
46.8 M
56.1 M
65.5 M
74.8 M
84.2 M
Legend
Identity
1
0.75
0.5
0.25
0

K15 - L8

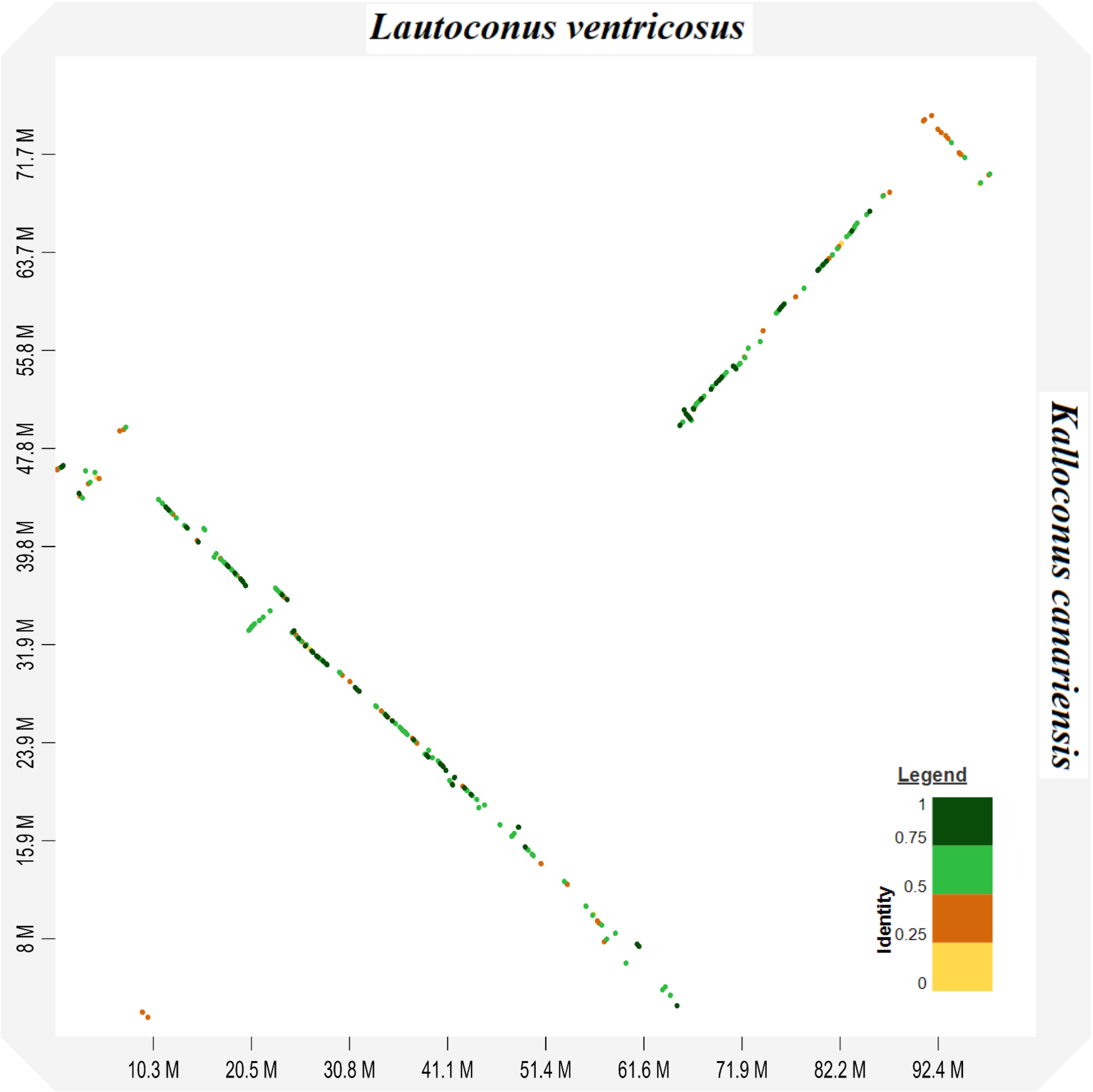

Lautoconus ventricosus
Kalloconus canariensis
71.7 M
63.7 M
55.8 M
47.8 M
39.8 M
31.9 M
23.9 M
15.9 M
8 M
10.3 M
20.5 M
30.8 M
41.1 M
51.4 M
61.6 M
71.9 M
82.2 M
92.4 M
Legend
Identity
1
0.75
0.5
0.25
0

K16 - L14

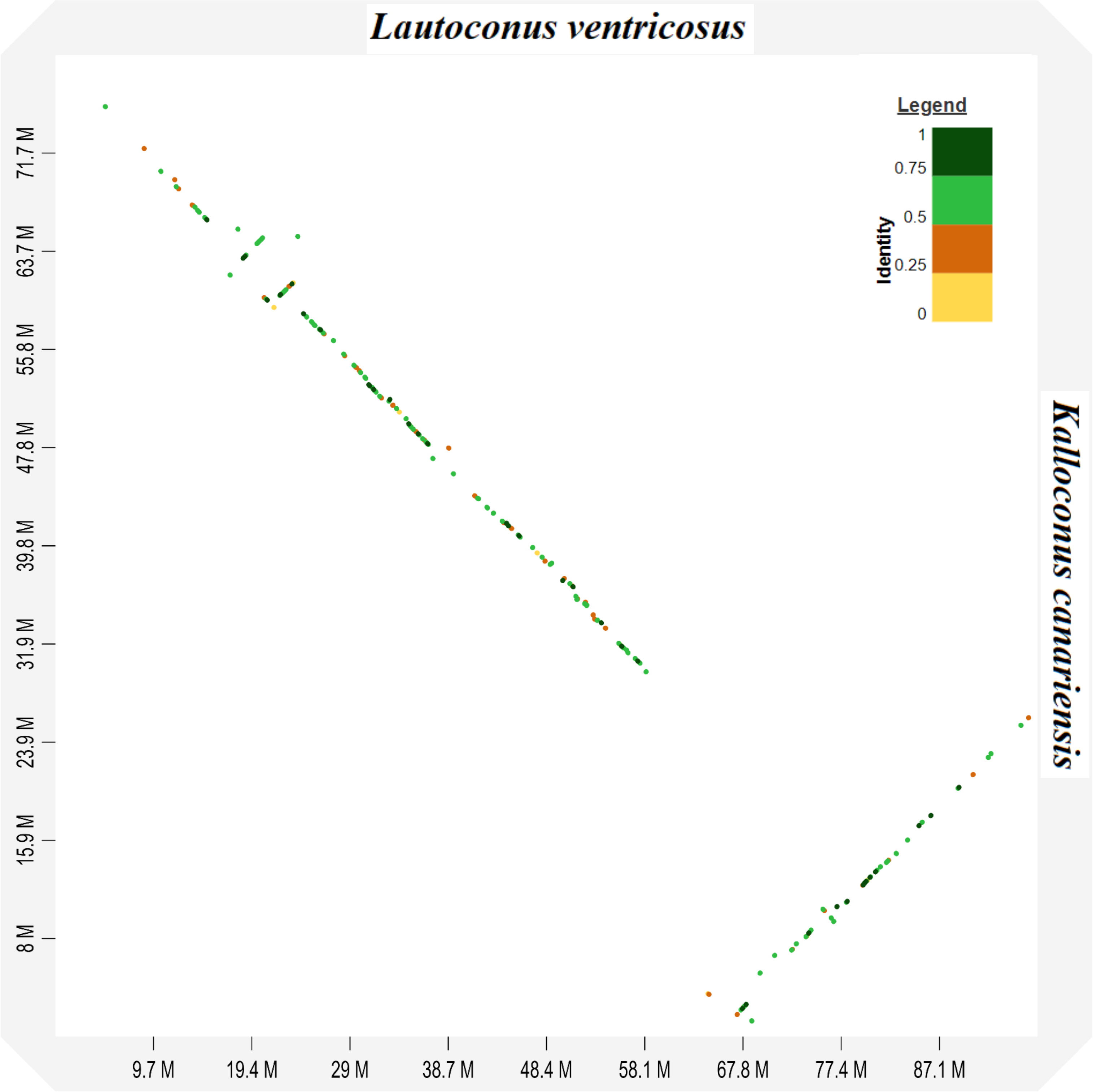

Lautoconus ventricosus
Kalloconus canariensis
Legend
Identity
1
0.75
0.5
0.25
0
71.7 M
63.7 M
55.8 M
47.8 M
39.8 M
31.9 M
23.9 M
15.9 M
8 M
9.7 M
19.4 M
29 M
38.7 M
48.4 M
58.1 M
67.8 M
77.4 M
87.1 M

K17 - L18

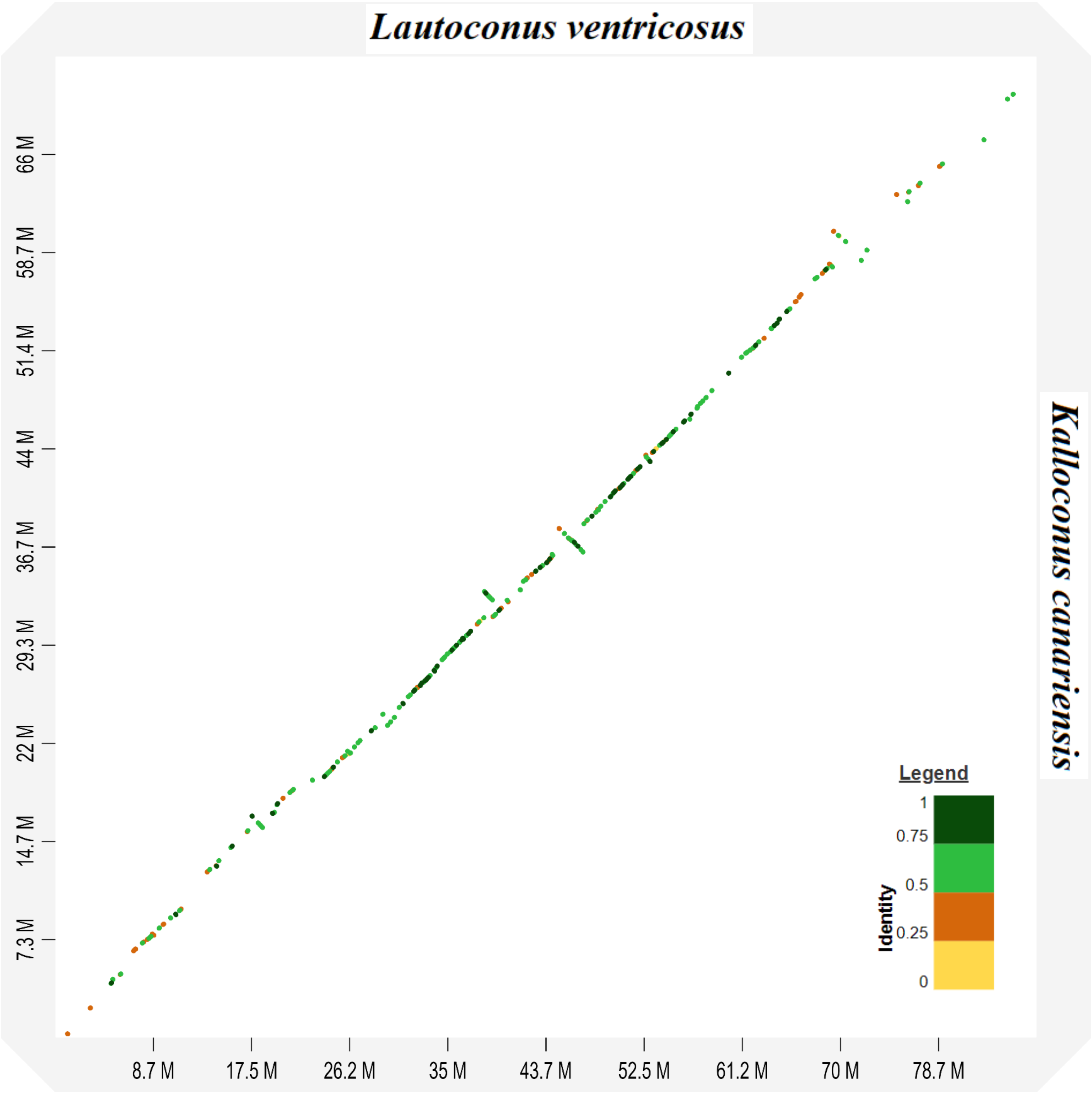

Lautoconus ventricosus
Kalloconus canariensis
66 M
58.7 M
51.4 M
44 M
36.7 M
29.3 M
22 M
14.7 M
7.3 M
8.7 M
17.5 M
26.2 M
35 M
43.7 M
52.5 M
61.2 M
70 M
78.7 M
Legend
Identity
1
0.75
0.5
0.25
0

K18 - L17

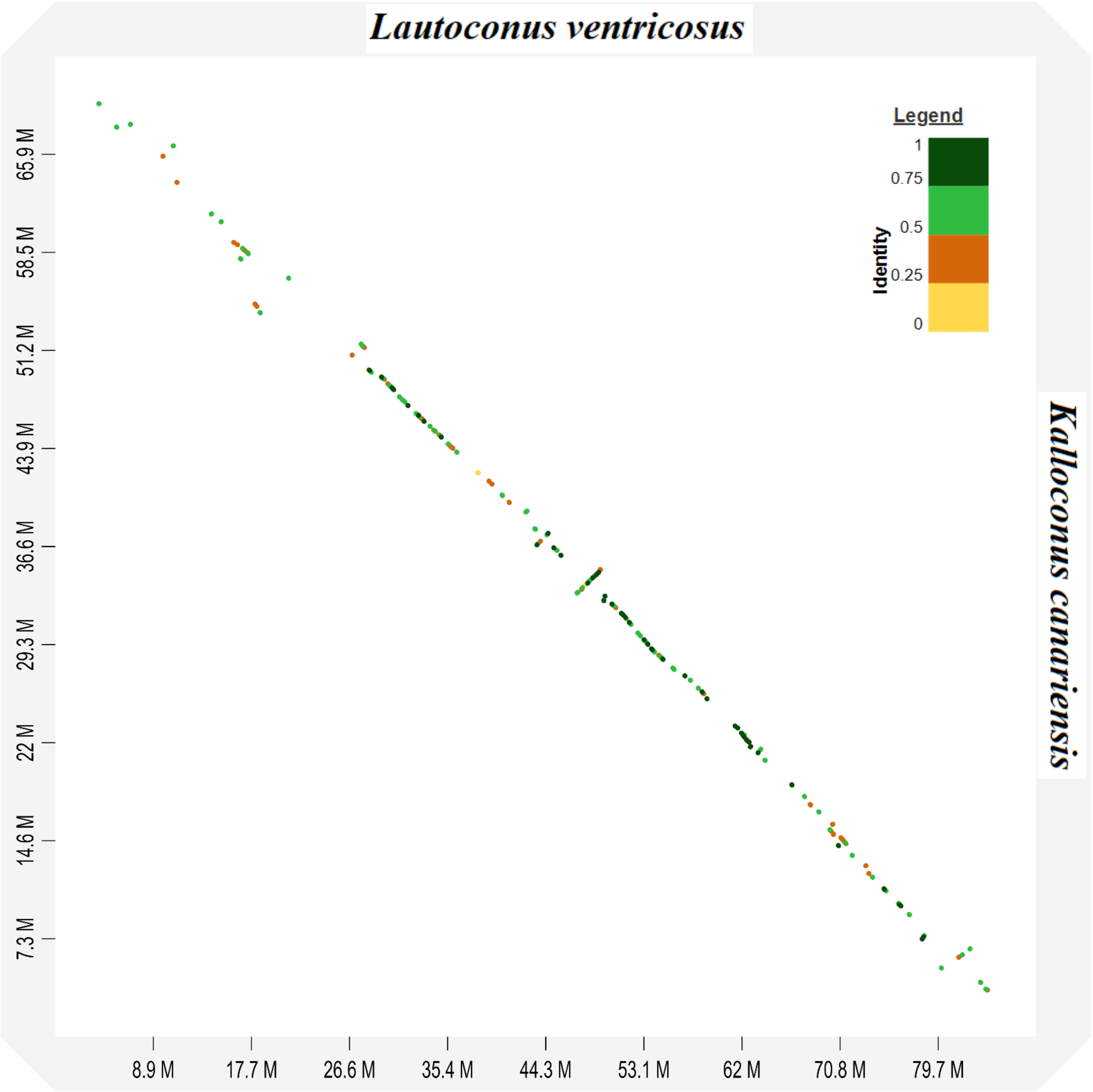

Lautoconus ventricosus
Kalloconus canariensis
Legend
Identity
1
0.75
0.5
0.25
0
65.9 M
58.5 M
51.2 M
43.9 M
36.6 M
29.3 M
22 M
14.6 M
7.3 M
8.9 M
17.7 M
26.6 M
35.4 M
44.3 M
53.1 M
62 M
70.8 M
79.7 M

K19 – L19

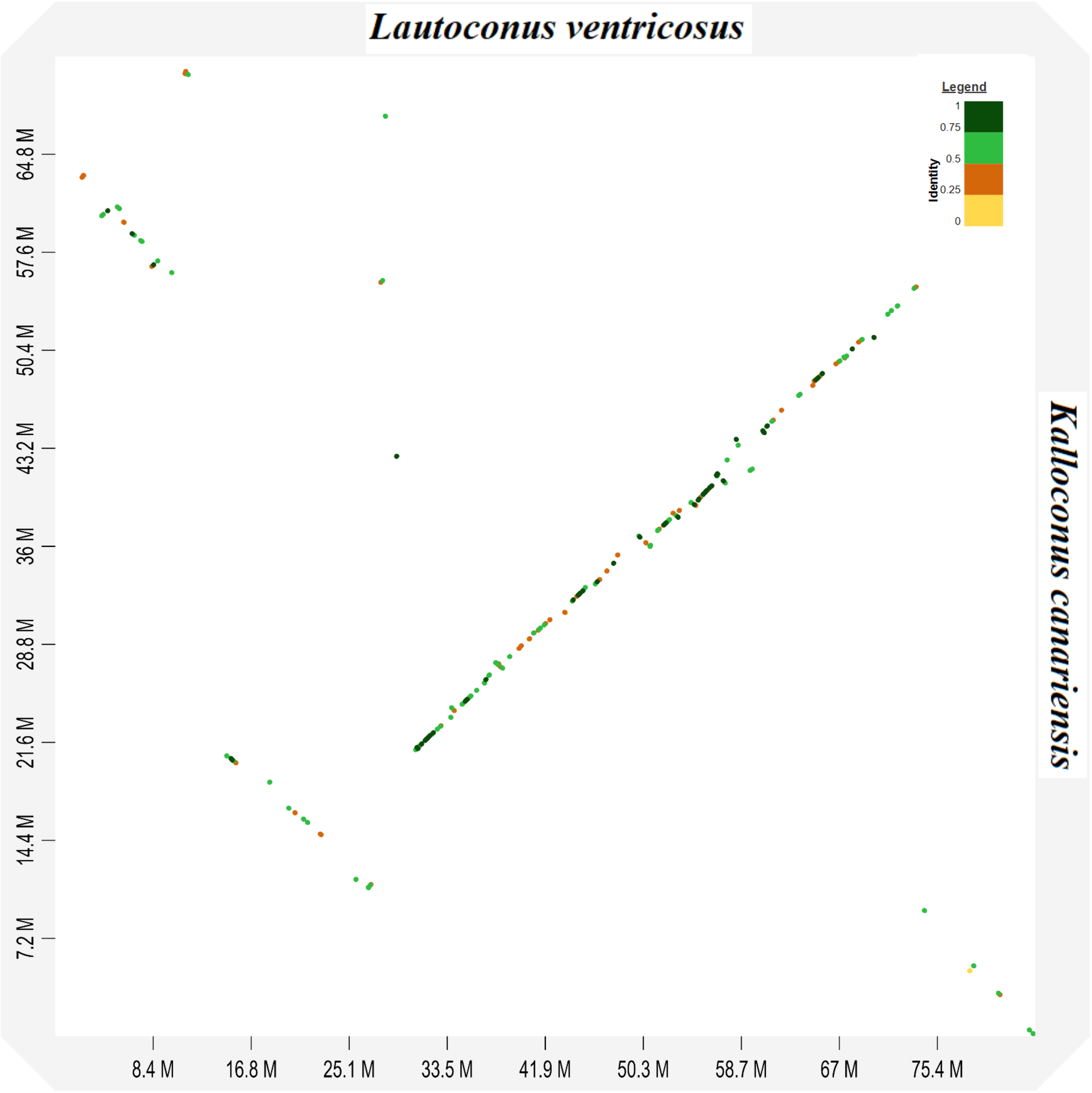

Lautoconus ventricosus
Kalloconus canariensis
Legend
Identity
1
0.75
0.5
0.25
0
64.8 M
57.6 M
50.4 M
43.2 M
36 M
28.8 M
21.6 M
14.4 M
7.2 M
8.4 M
16.8 M
25.1 M
33.5 M
41.9 M
50.3 M
58.7 M
67 M
75.4 M

K20 - L20

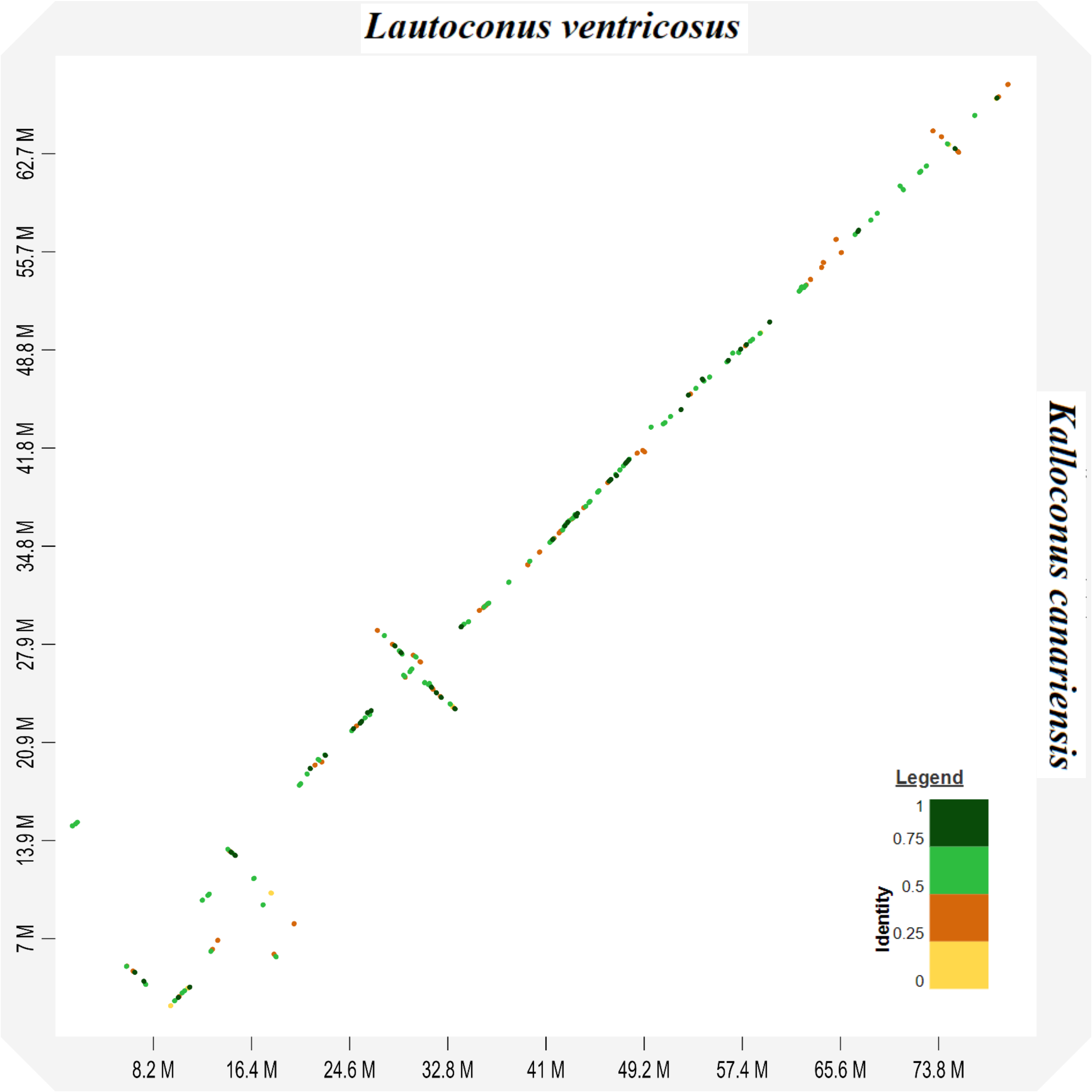

Lautoconus ventricosus
Kalloconus canariensis
62.7 M
55.7 M
48.8 M
41.8 M
34.8 M
27.9 M
20.9 M
13.9 M
7 M
8.2 M
16.4 M
24.6 M
32.8 M
41 M
49.2 M
57.4 M
65.6 M
73.8 M
Legend
Identity
1
0.75
0.5
0.25
0

K21 - L21

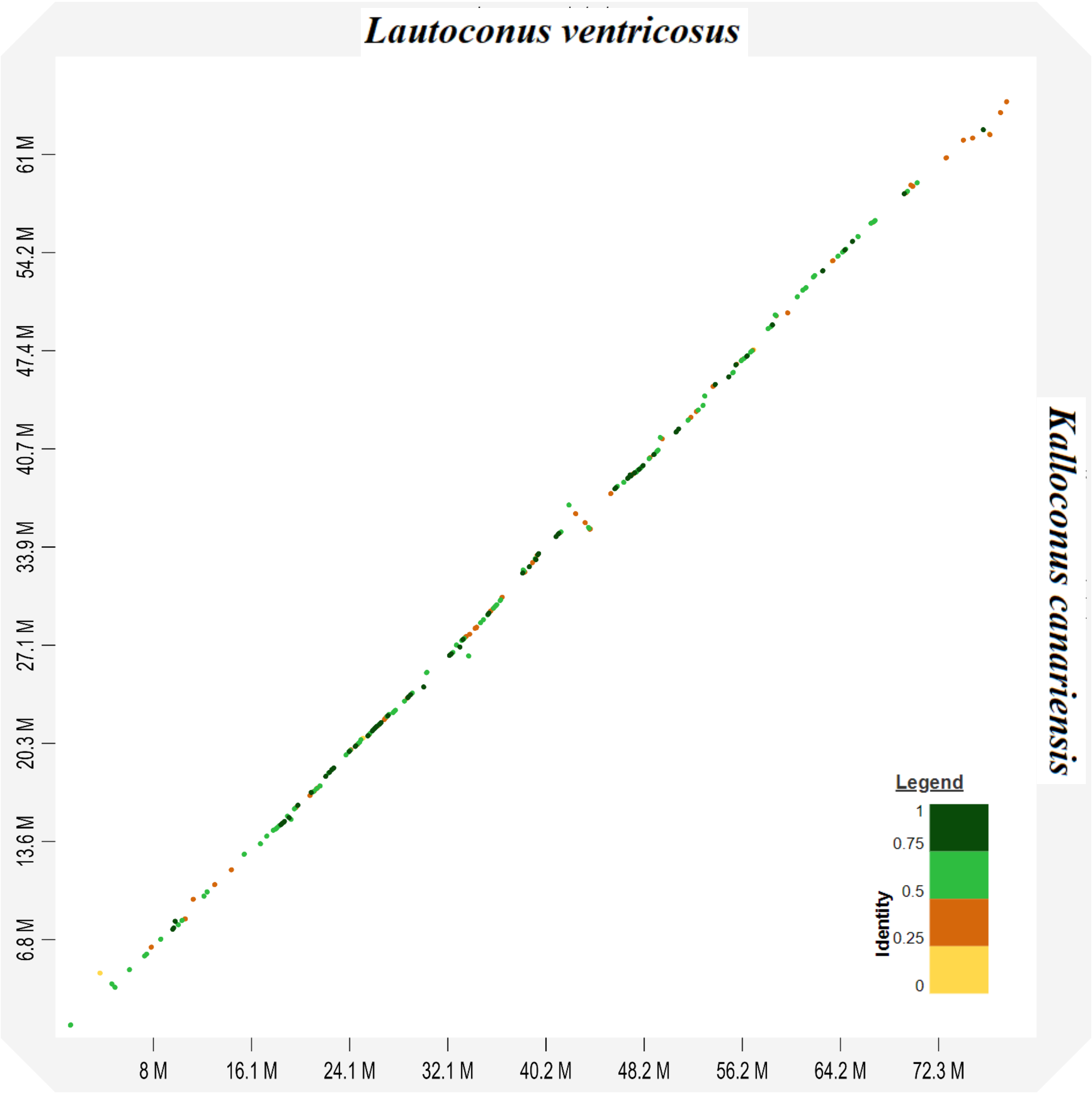
Lautoconus ventricosus
Kalloconus canariensis
61 M
54.2 M
47.4 M
40.7 M
33.9 M
27.1 M
20.3 M
13.6 M
6.8 M
8 M
16.1 M
24.1 M
32.1 M
40.2 M
48.2 M
56.2 M
64.2 M
72.3 M
Legend
Identity
1
0.75
0.5
0.25
0

K22 - L24

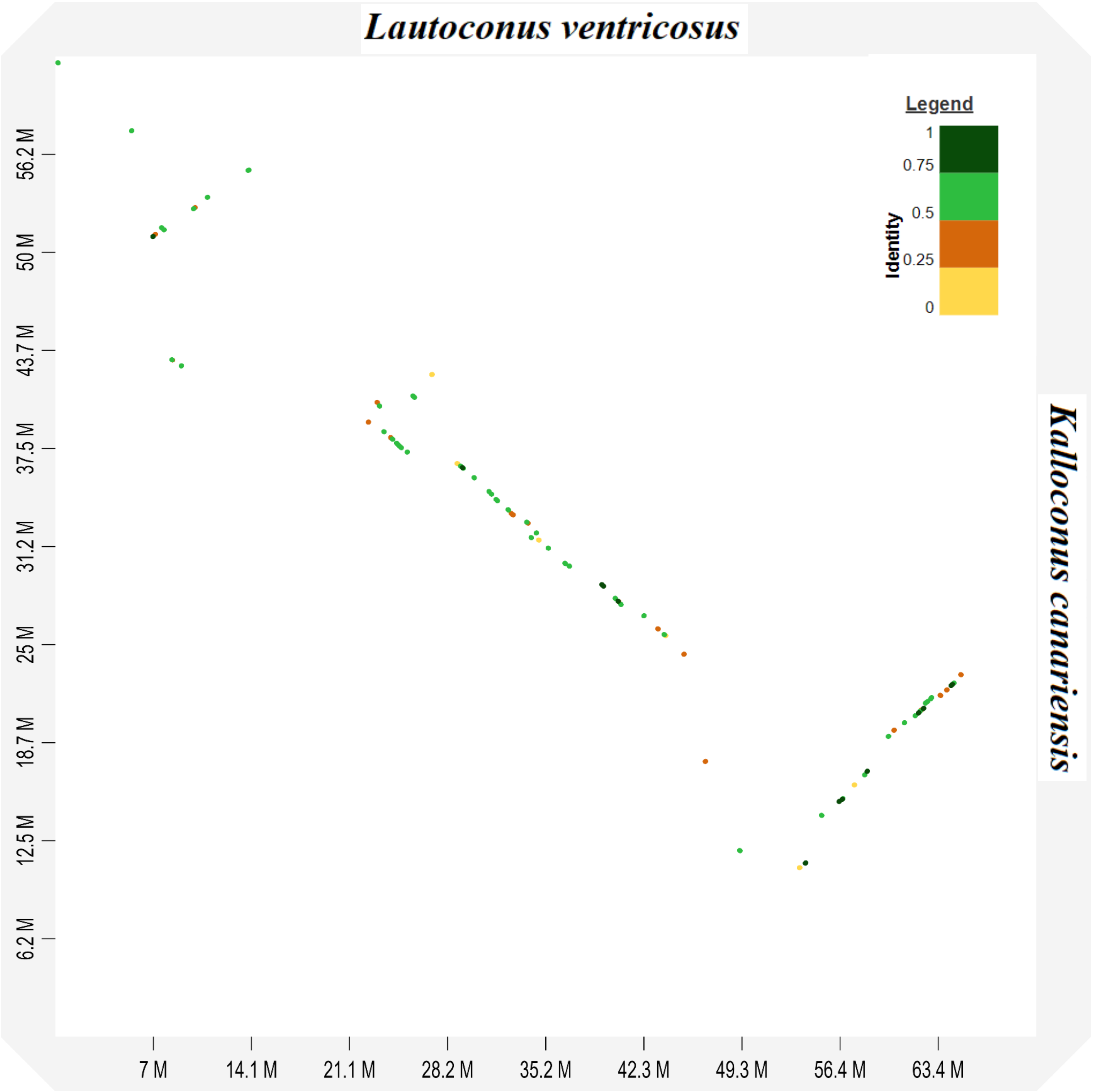

Lautoconus ventricosus
Kalloconus canariensis
Legend
Identity
1
0.75
0.5
0.25
0
56.2 M
50 M
43.7 M
37.5 M
31.2 M
25 M
18.7 M
12.5 M
6.2 M
7 M
14.1 M
21.1 M
28.2 M
35.2 M
42.3 M
49.3 M
56.4 M
63.4 M

K23 - K27

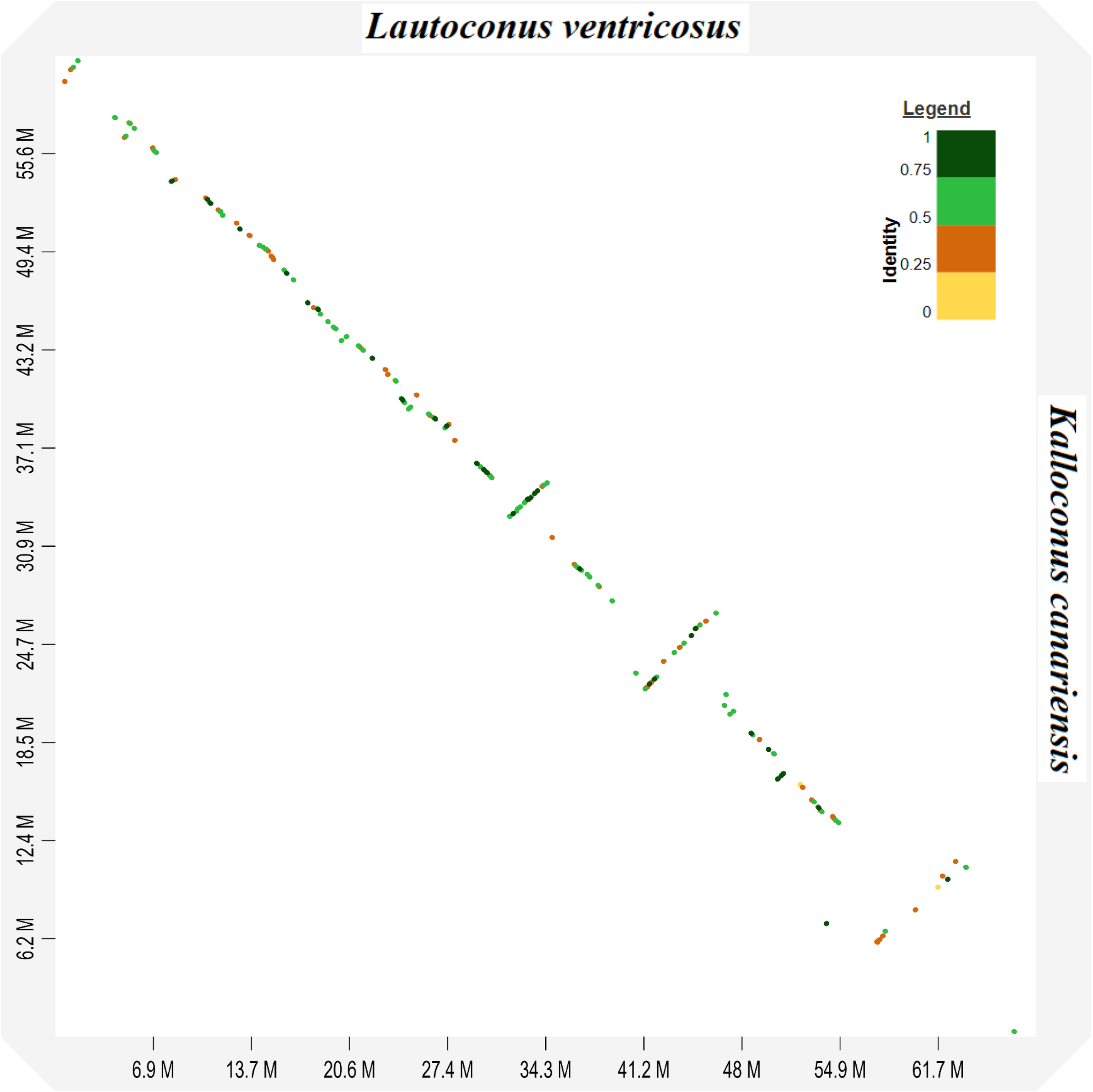

Lautoconus ventricosus
Kalloconus canariensis
Legend
Identity
1
0.75
0.5
0.25
0
55.6 M
49.4 M
43.2 M
37.1 M
30.9 M
24.7 M
18.5 M
12.4 M
6.2 M
6.9 M
13.7 M
20.6 M
27.4 M
34.3 M
41.2 M
48 M
54.9 M
61.7 M

K24 - L22

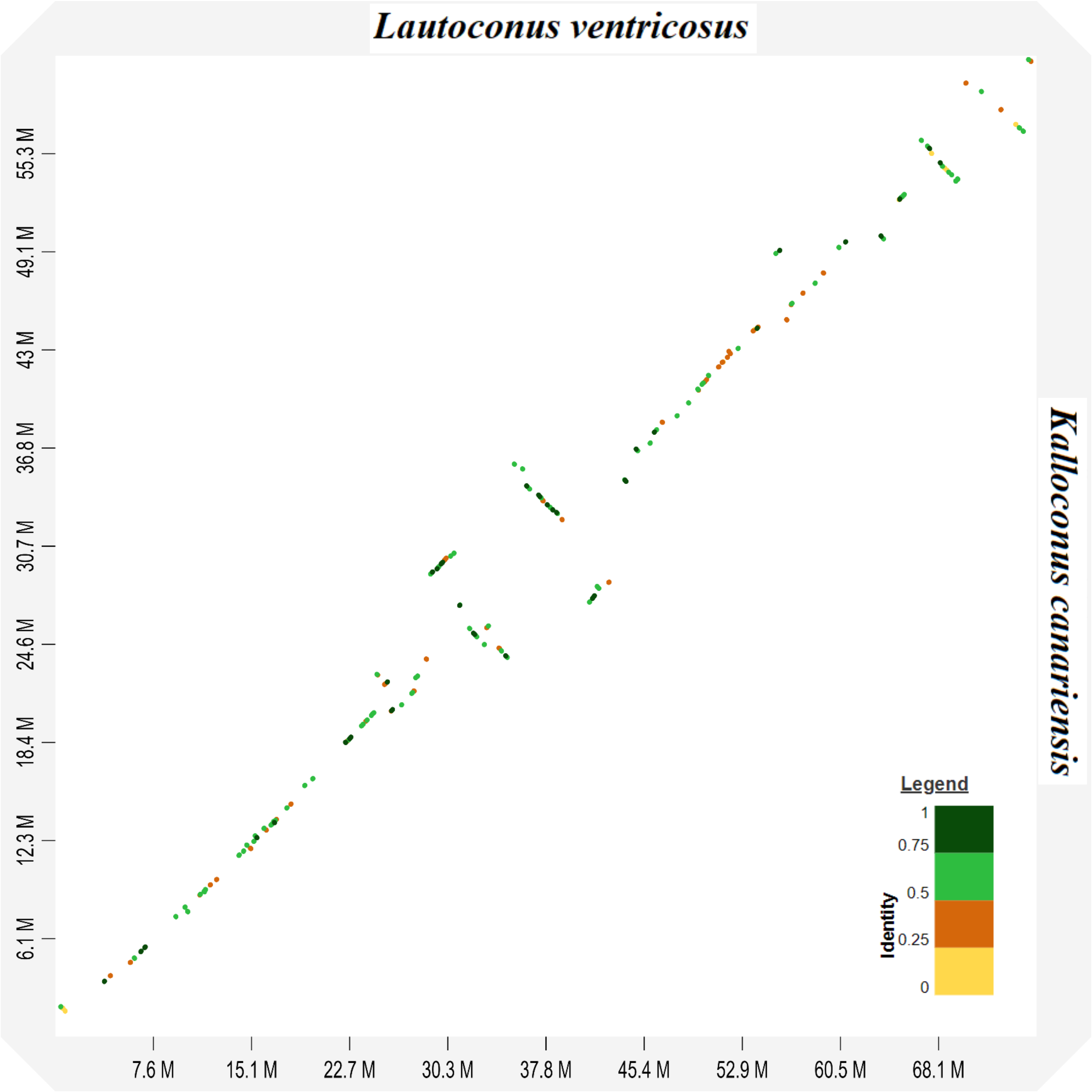

Lautoconus ventricosus
Kalloconus canariensis
55.3 M
49.1 M
43 M
36.8 M
30.7 M
24.6 M
18.4 M
12.3 M
6.1 M
7.6 M
15.1 M
22.7 M
30.3 M
37.8 M
45.4 M
52.9 M
60.5 M
68.1 M
Legend
Identity
1
0.75
0.5
0.25
0

K25 - L23

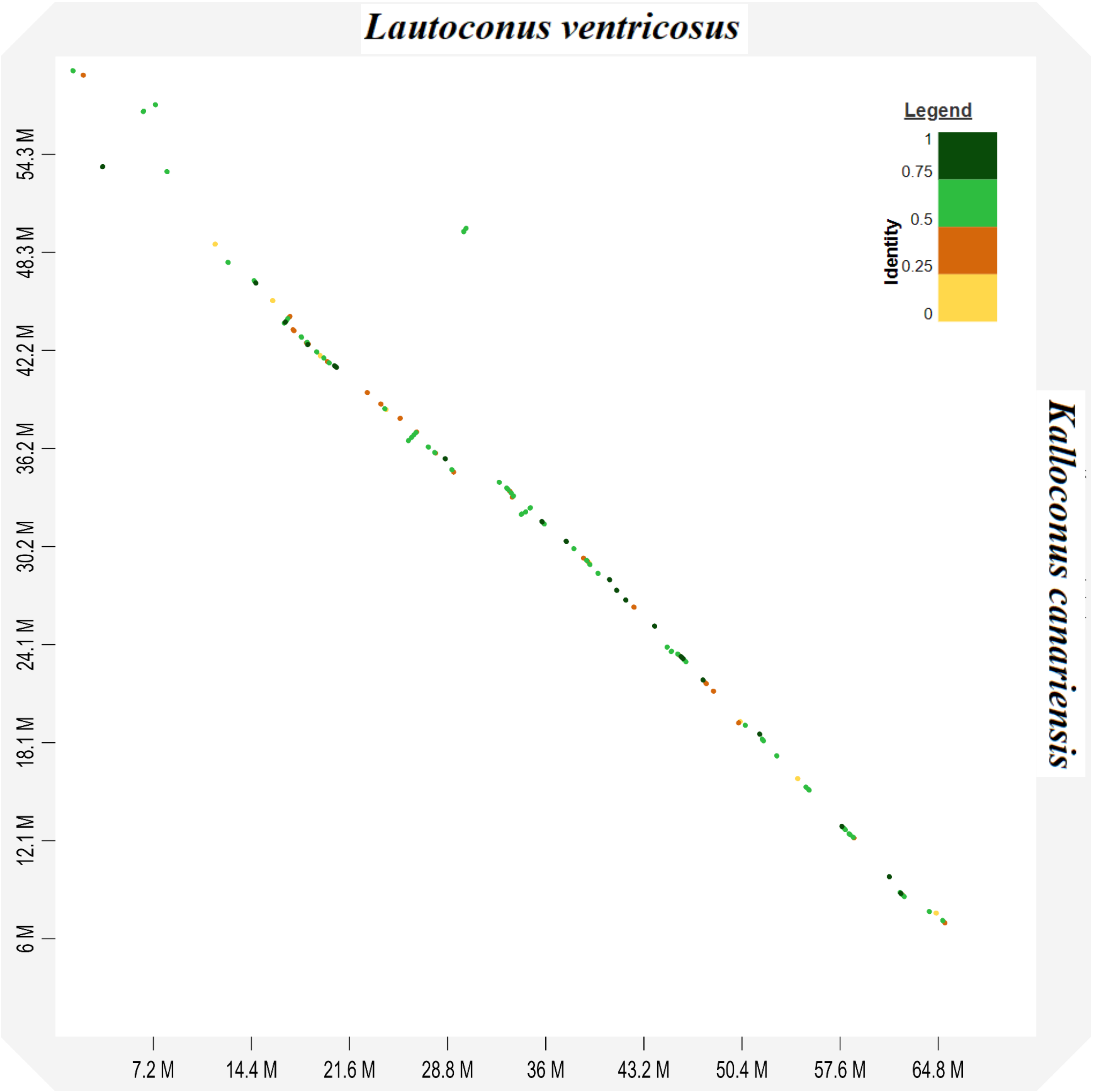

Lautoconus ventricosus
Kalloconus canariensis
Legend
Identity
1
0.75
0.5
0.25
0
54.3 M
48.3 M
42.2 M
36.2 M
30.2 M
24.1 M
18.1 M
12.1 M
6 M
7.2 M
14.4 M
21.6 M
28.8 M
36 M
43.2 M
50.4 M
57.6 M
64.8 M

K26 - L25

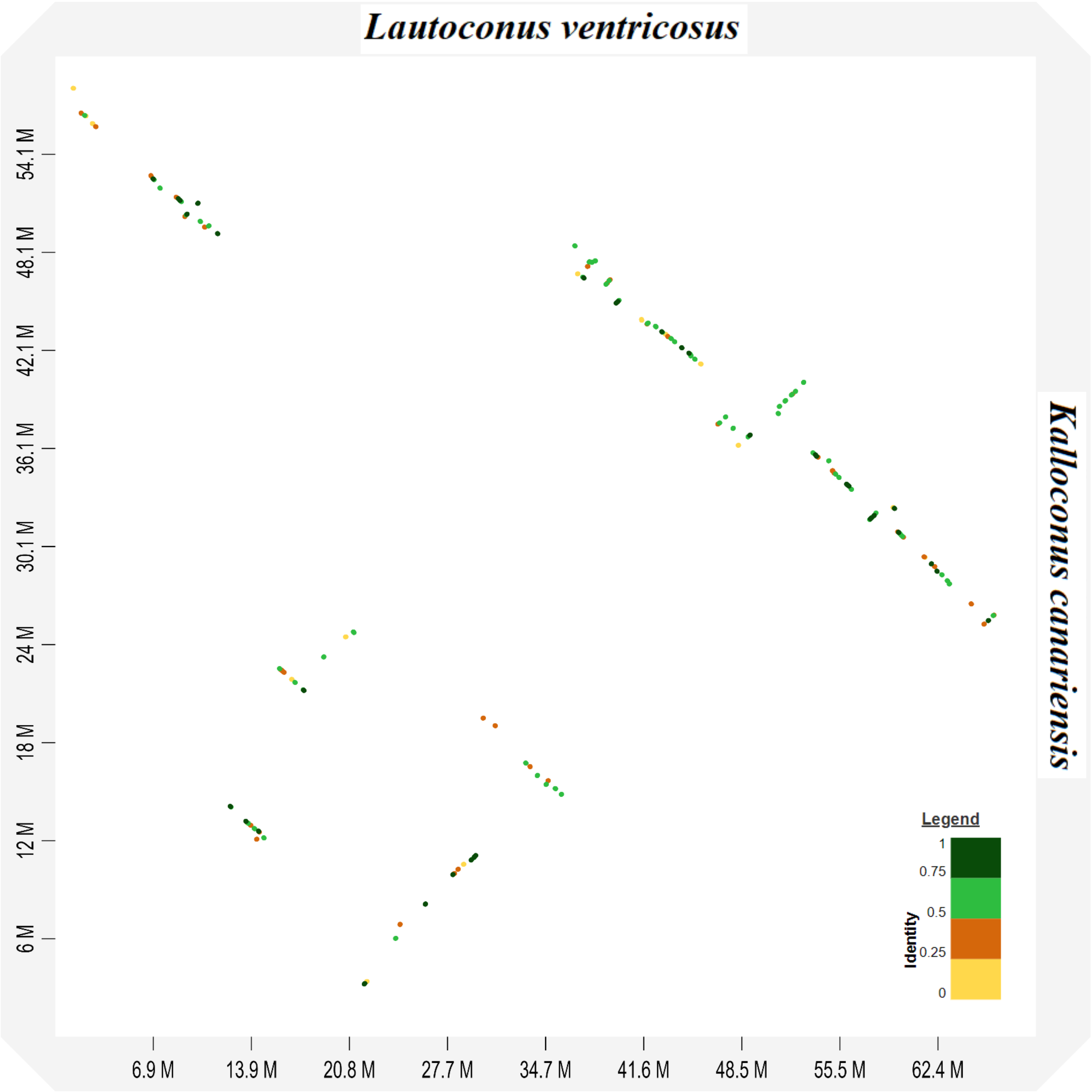

Lautoconus ventricosus
Kalloconus canariensis
54.1 M
48.1 M
42.1 M
36.1 M
30.1 M
24 M
18 M
12 M
6 M
6.9 M
13.9 M
20.8 M
27.7 M
34.7 M
41.6 M
48.5 M
55.5 M
62.4 M
Legend
Identity
1
0.75
0.5
0.25
0

K27 - L26

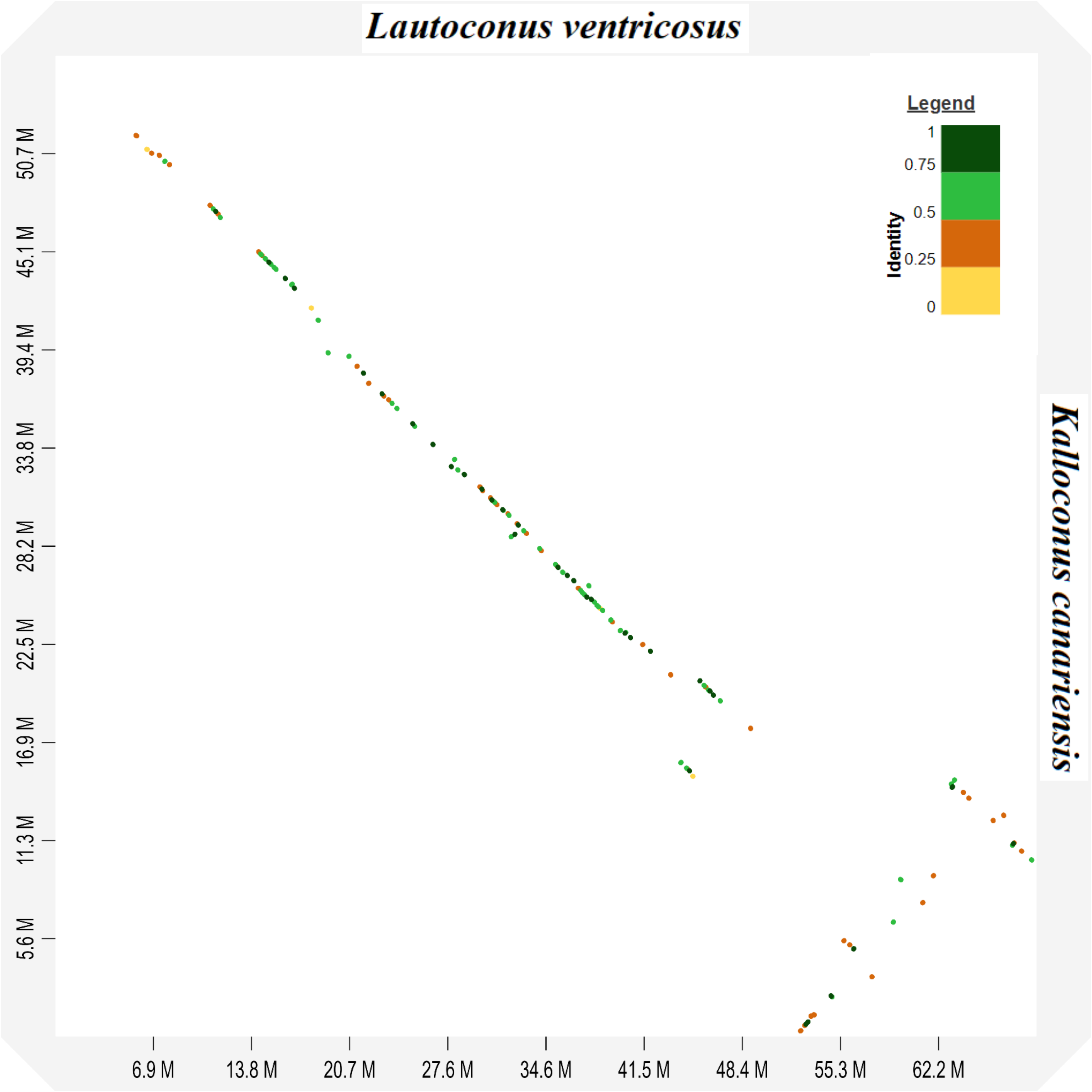

Lautoconus ventricosus
Kalloconus canariensis
Legend
Identity
1
0.75
0.5
0.25
0
50.7 M
45.1 M
39.4 M
33.8 M
28.2 M
22.5 M
16.9 M
11.3 M
5.6 M
6.9 M
13.8 M
20.7 M
27.6 M
34.6 M
41.5 M
48.4 M
55.3 M
62.2 M

K28 - L29

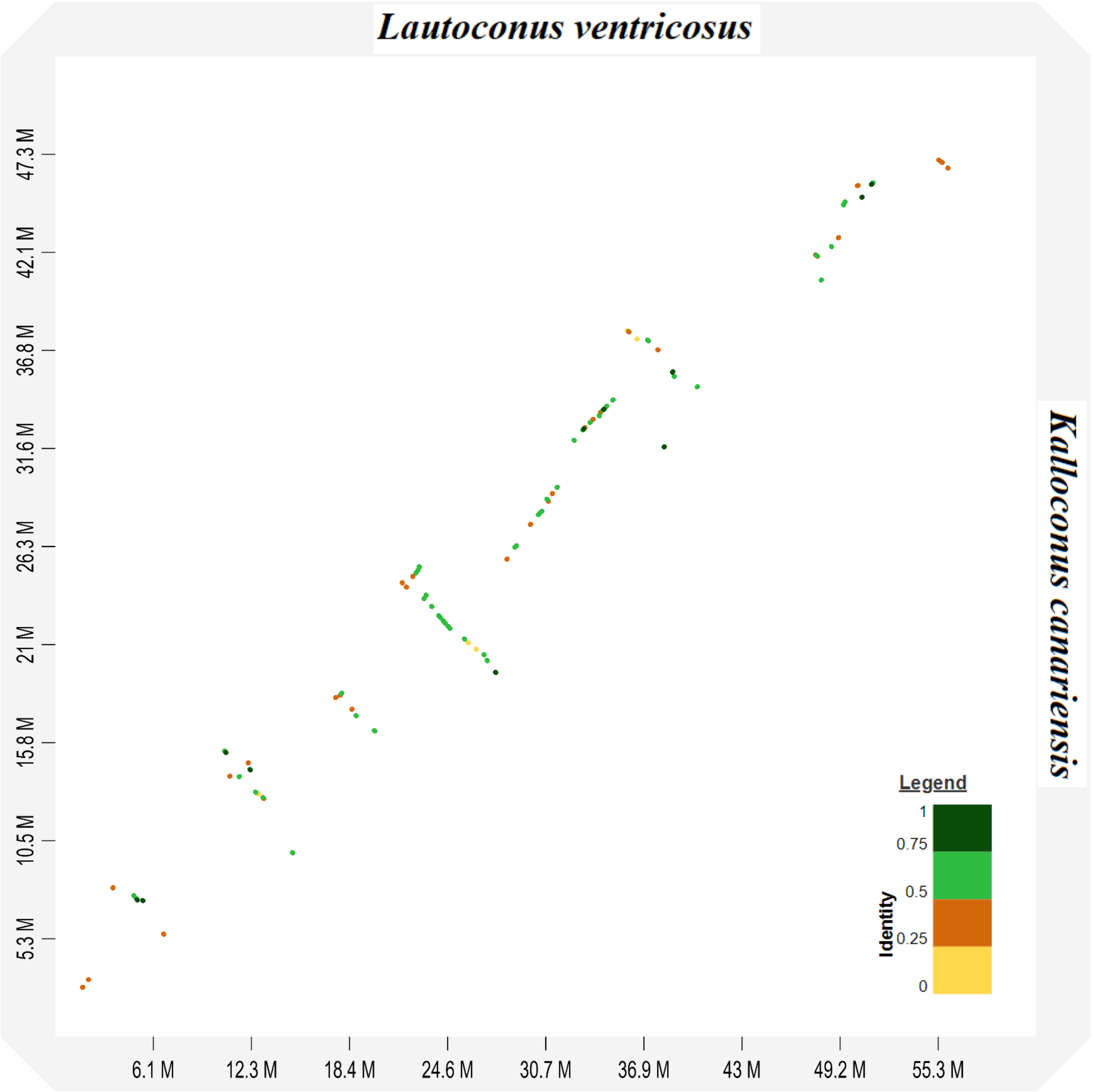

Lautoconus ventricosus
Kalloconus canariensis
47.3 M
42.1 M
36.8 M
31.6 M
26.3 M
21 M
15.8 M
10.5 M
5.3 M
6.1 M
12.3 M
18.4 M
24.6 M
30.7 M
36.9 M
43 M
49.2 M
55.3 M
Legend
Identity
1
0.75
0.5
0.25
0

K29 - L28

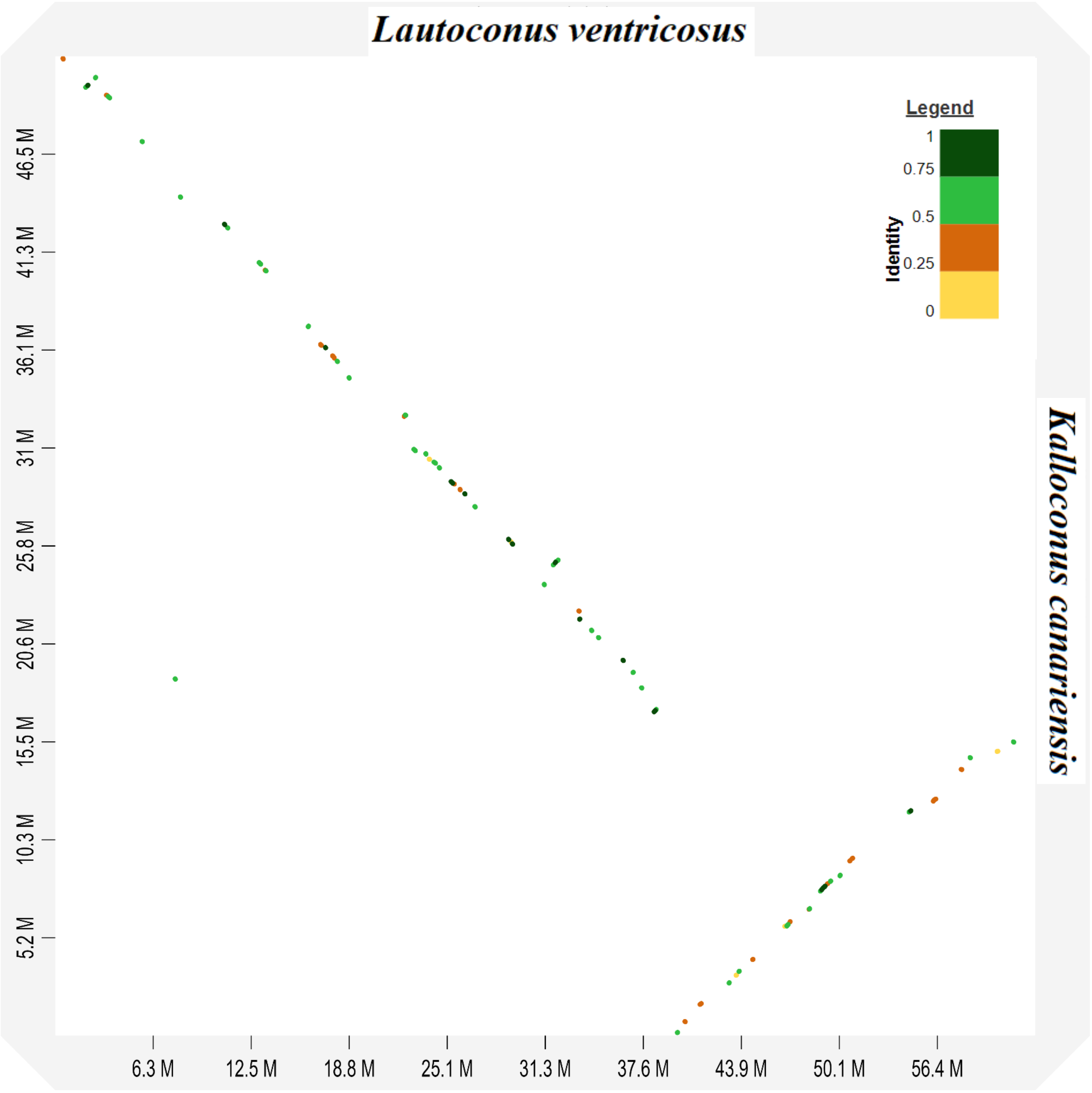

Lautoconus ventricosus
Kalloconus canariensis
Legend
Identity
1
0.75
0.5
0.25
0
46.5 M
41.3 M
36.1 M
31 M
25.8 M
20.6 M
15.5 M
10.3 M
5.2 M
6.3 M
12.5 M
18.8 M
25.1 M
31.3 M
37.6 M
43.9 M
50.1 M
56.4 M

K30 - L32

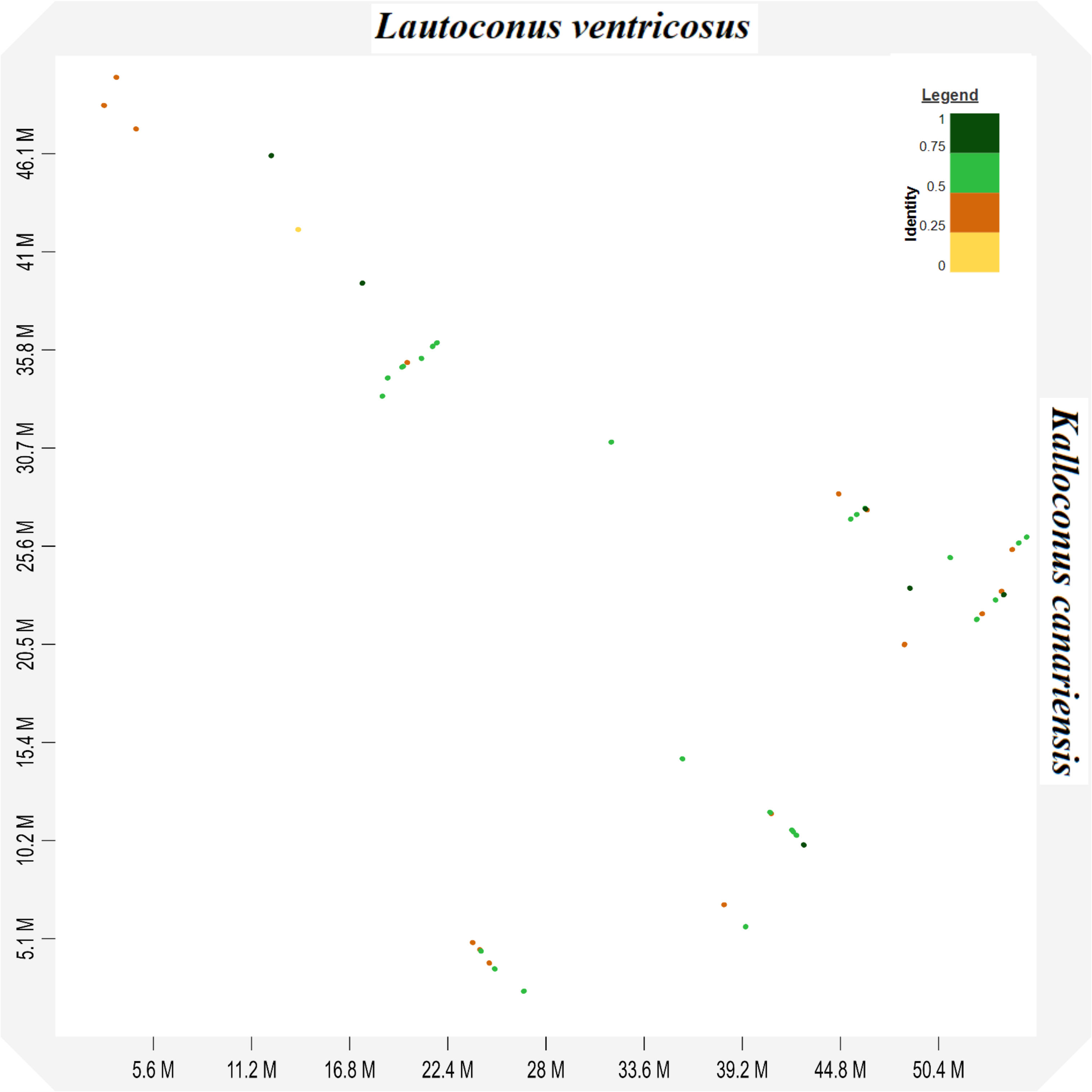
Lautoconus ventricosus
Kalloconus canariensis
Legend
Identity
1
0.75
0.5
0.25
0
46.1 M
41 M
35.8 M
30.7 M
25.6 M
20.5 M
15.4 M
10.2 M
5.1 M
5.6 M
11.2 M
16.8 M
22.4 M
28 M
33.6 M
39.2 M
44.8 M
50.4 M

K31 - L30

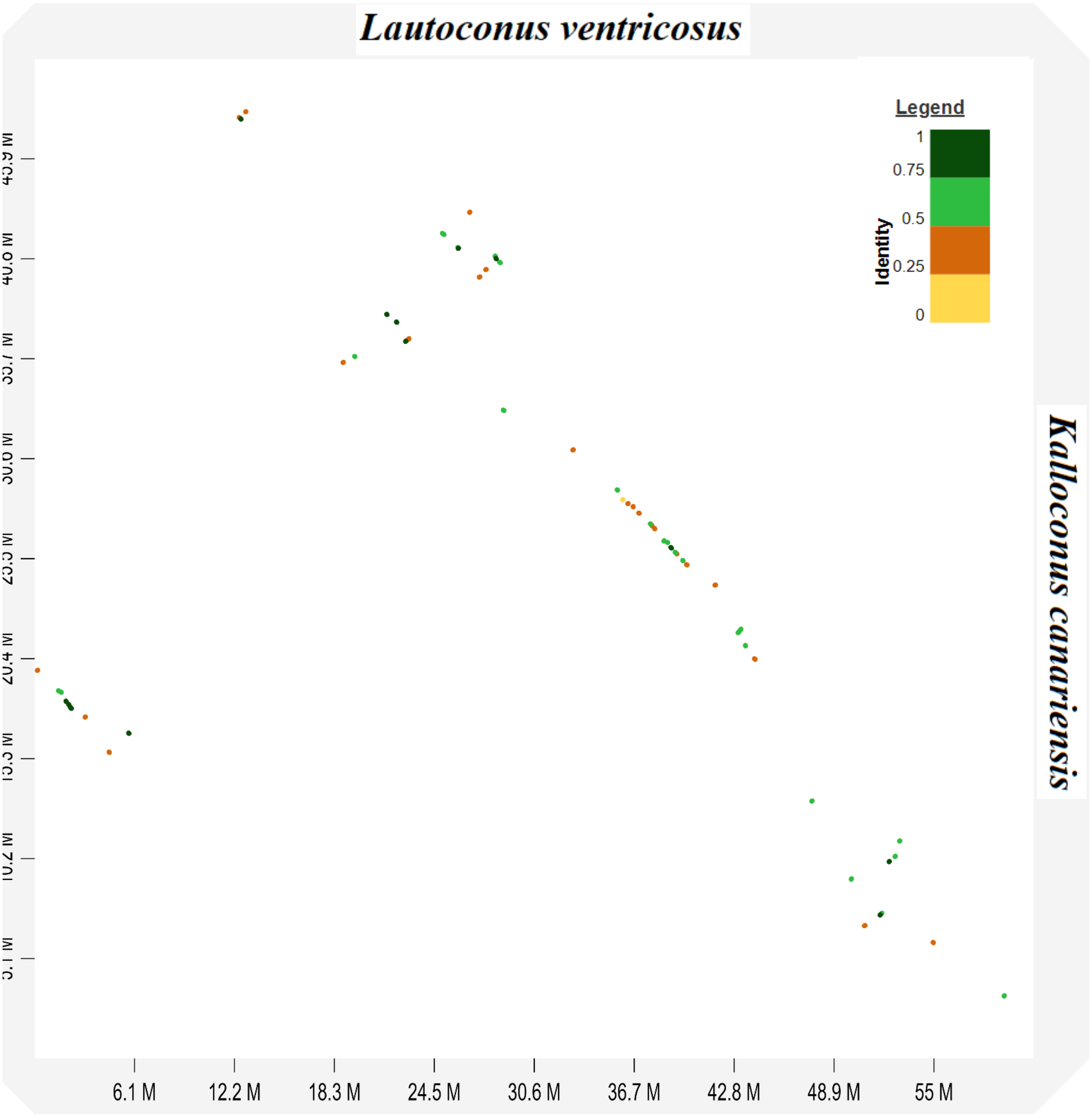
Lautoconus ventricosus
Kalloconus canariensis
Legend
Identity
1
0.75
0.5
0.25
0
6.1 M
12.2 M
18.3 M
24.5 M
30.6 M
36.7 M
42.8 M
48.9 M
55 M

K32 - L33

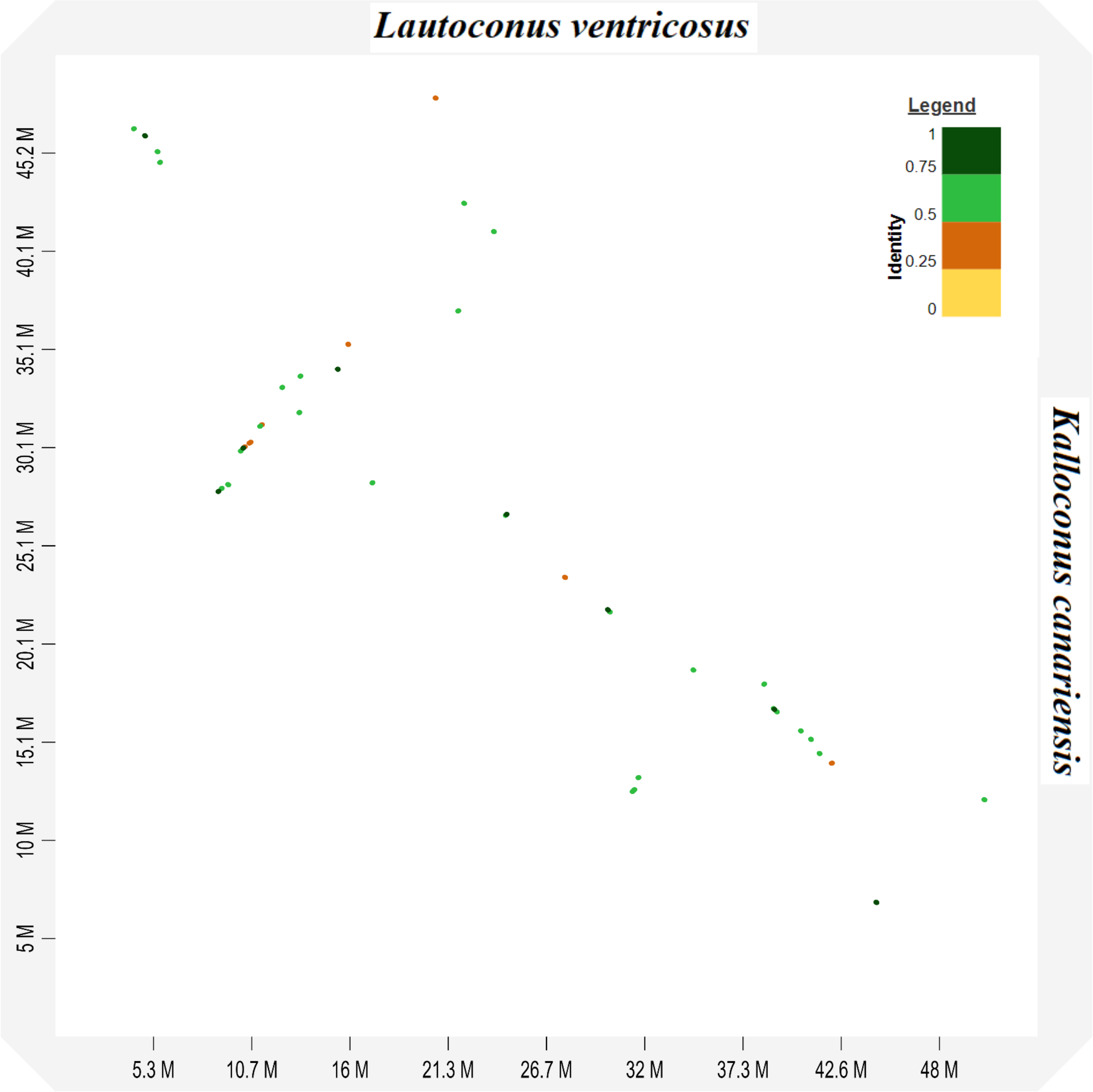

Lautoconus ventricosus
Kalloconus canariensis
Legend
Identity
1
0.75
0.5
0.25
0
45.2 M
40.1 M
35.1 M
30.1 M
25.1 M
20.1 M
15.1 M
10 M
5 M
5.3 M
10.7 M
16 M
21.3 M
26.7 M
32 M
37.3 M
42.6 M
48 M

K33 - L31

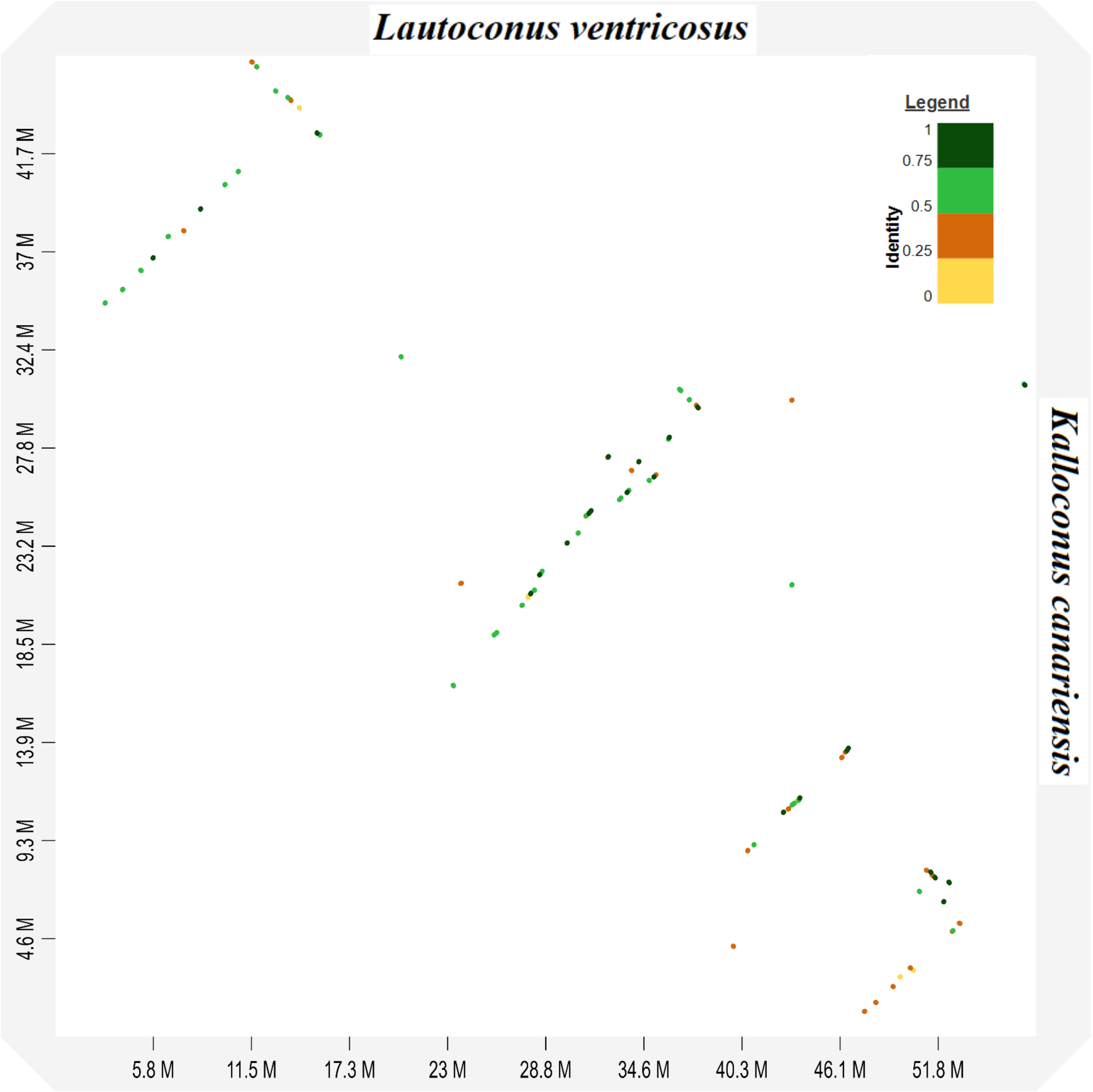

Lautoconus ventricosus
Kalloconus canariensis
Legend
Identity
1
0.75
0.5
0.25
0
41.7 M
37 M
32.4 M
27.8 M
23.2 M
18.5 M
13.9 M
9.3 M
4.6 M
5.8 M
11.5 M
17.3 M
23 M
28.8 M
34.6 M
40.3 M
46.1 M
51.8 M

K34 - L35

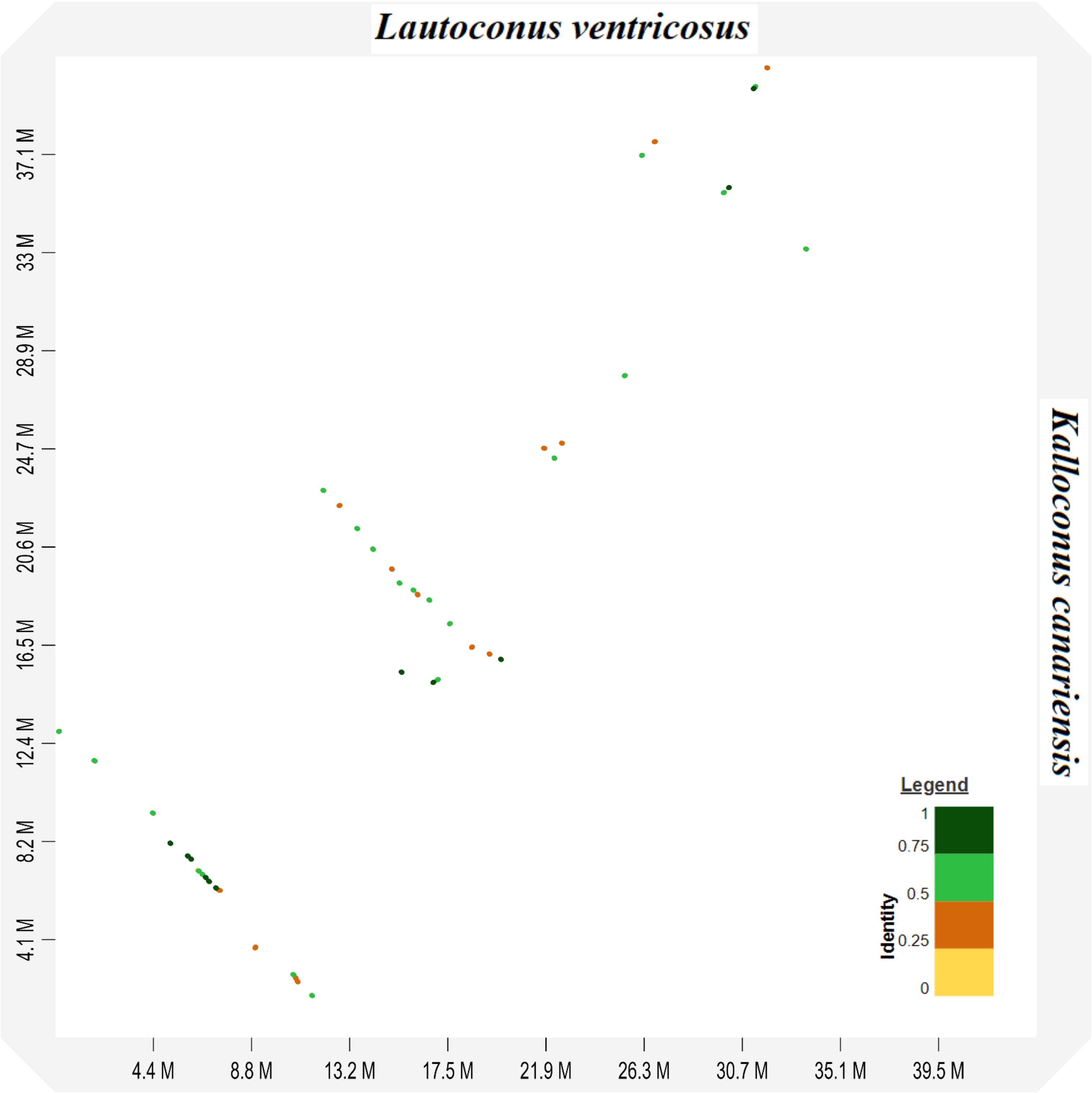
Lautoconus ventricosus
Kalloconus canariensis
37.1 M
33 M
28.9 M
24.7 M
20.6 M
16.5 M
12.4 M
8.2 M
4.1 M
4.4 M
8.8 M
13.2 M
17.5 M
21.9 M
26.3 M
30.7 M
35.1 M
39.5 M
Legend
Identity
1
0.75
0.5
0.25
0

K35 - L34

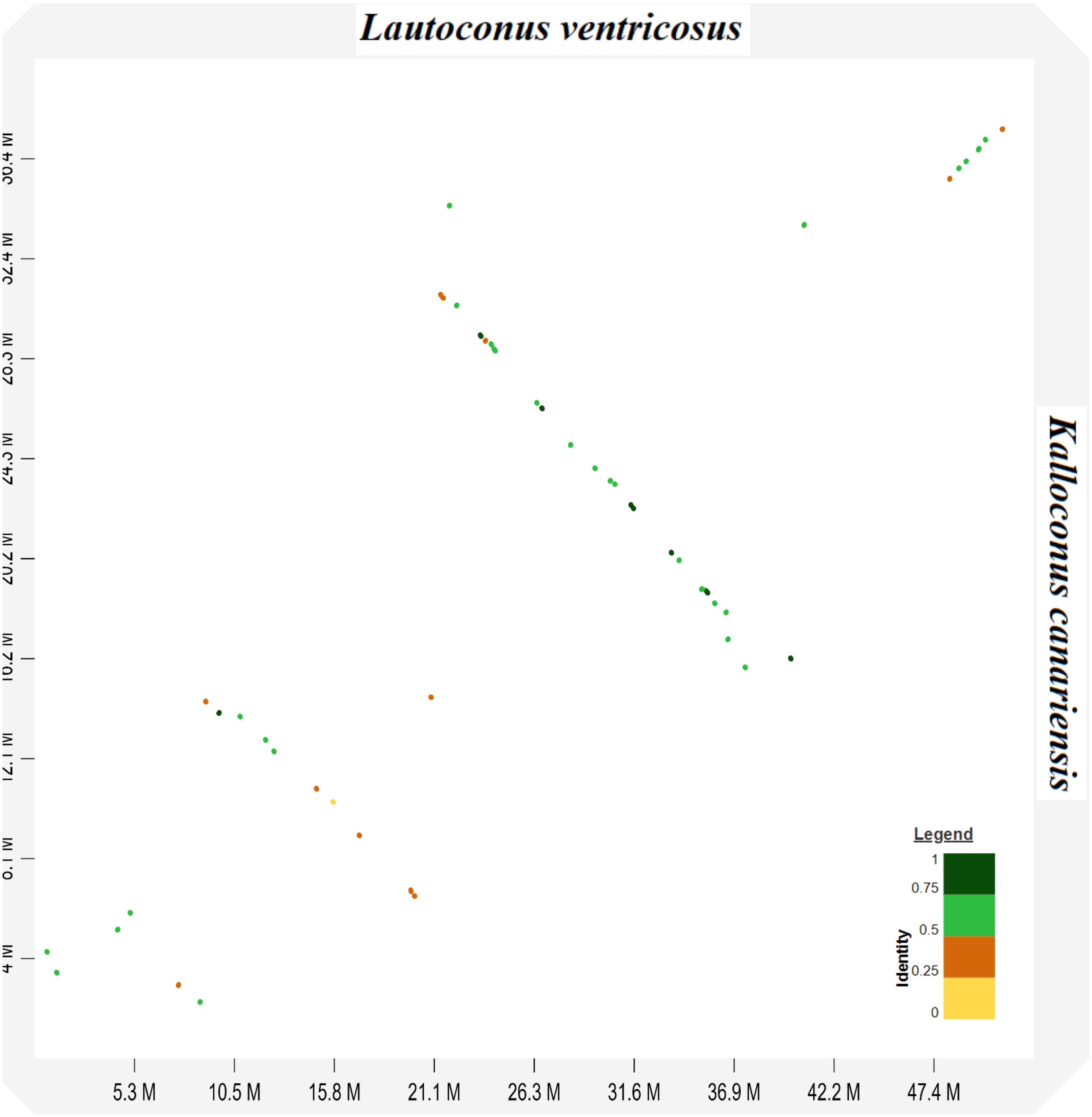
Lautoconus ventricosus
Kalloconus canariensis
5.3 M
10.5 M
15.8 M
21.1 M
26.3 M
31.6 M
36.9 M
42.2 M
47.4 M
Legend
1
0.75
0.5
0.25
0
Identity

Fig. S4. Maximum likelihood phylogenomic relationships of Caenogastropoda (green branches) based on 57 single copy proteins of 20 gastropod species. Neritimorpha and Heterobranchia were used as outgroups. Neogastropoda and Conidae are depicted with slashed purple and red boxes, respectively. Numbers at nodes are boostrap support values. The circle represents an ancestral Whole Genome Duplication (WGD) event, as reported in Farhat et al. (2023). The reconstructed tree was visualized with FigTree v1.4.4 (http://tree.bio.ed.ac.uk/software/figtree/).

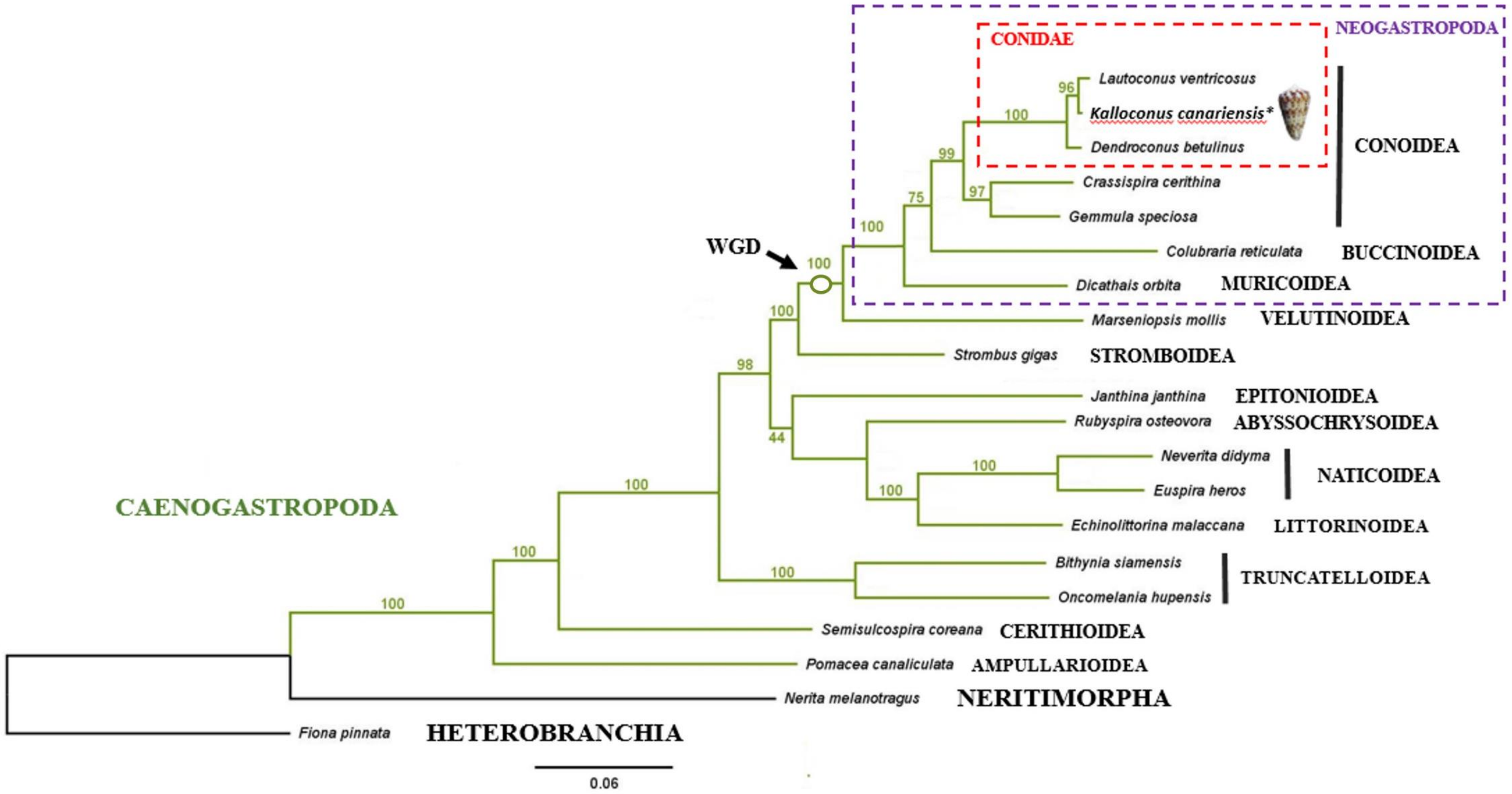

Farhat S, Modica MV, Puillandre N. Whole Genome Duplication and Gene Evolution in the Hyperdiverse Venomous Gastropods, *Mol. Biol. Evol.* 2023; 40(8):msad171. https://doi.org/10.1093/molbev/msad171

Fig. S5. Assesment of the *Kalloconus canariensis* genome assembly.

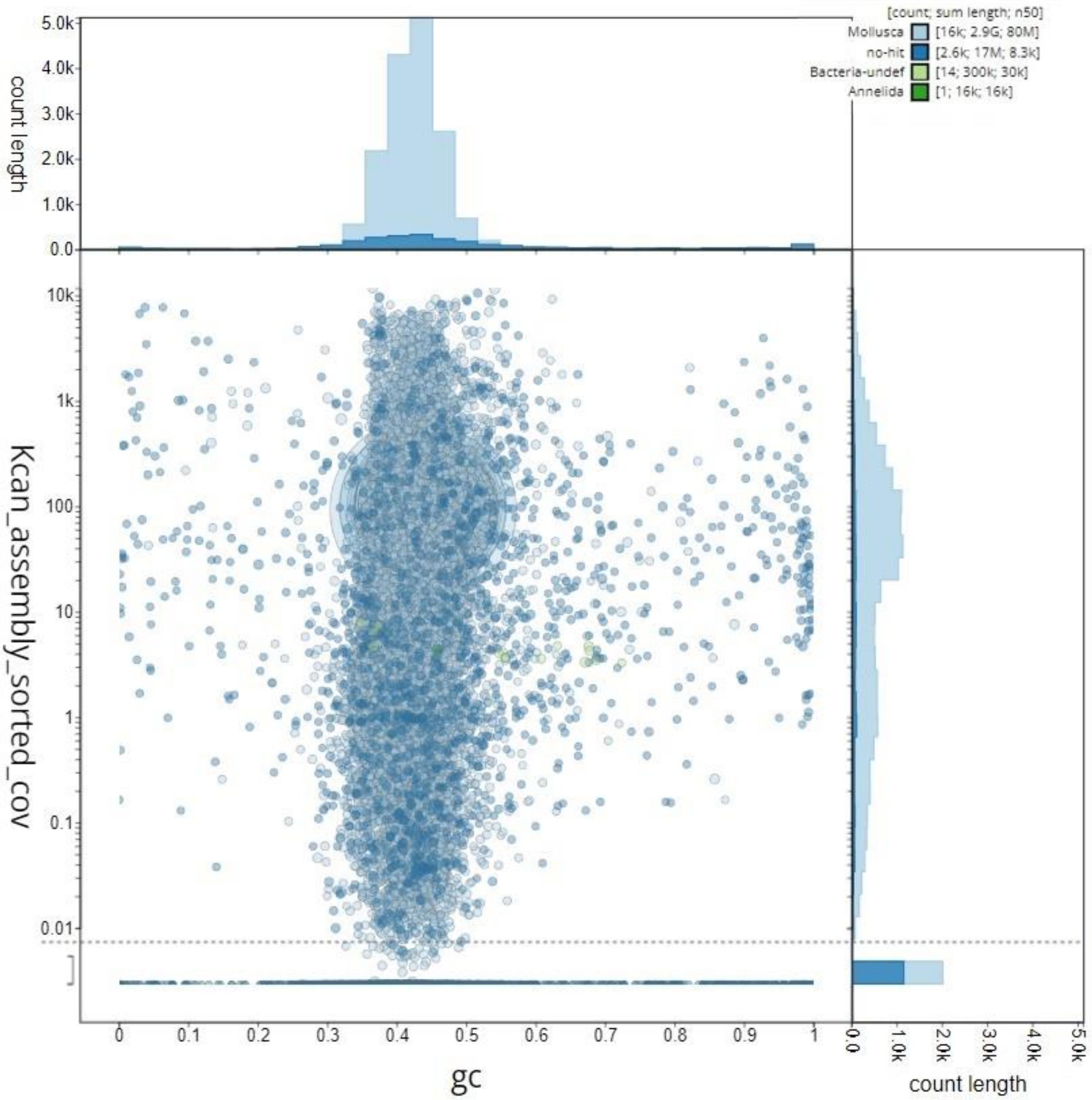

Blob plot of base coverage in Kcan against GC proportion for sequences in assembly B_results_blobtools. Sequences are coloured by phylum. Circles are sized in proportion to sequence length on a square-root scale, ranging from 210 to 153.129.599. Histograms show the distribution of sequence length sum along each axis .

length	
total	2,868,193,718
Mollusca	2,850,786,852
no-hit	17,092,293
Bacteria-undef	298,112
Annelida	16,461

counts	
total	18,573
Mollusca	15,932
no-hit	2,626
Bacteria-undef	14
Annelida	1

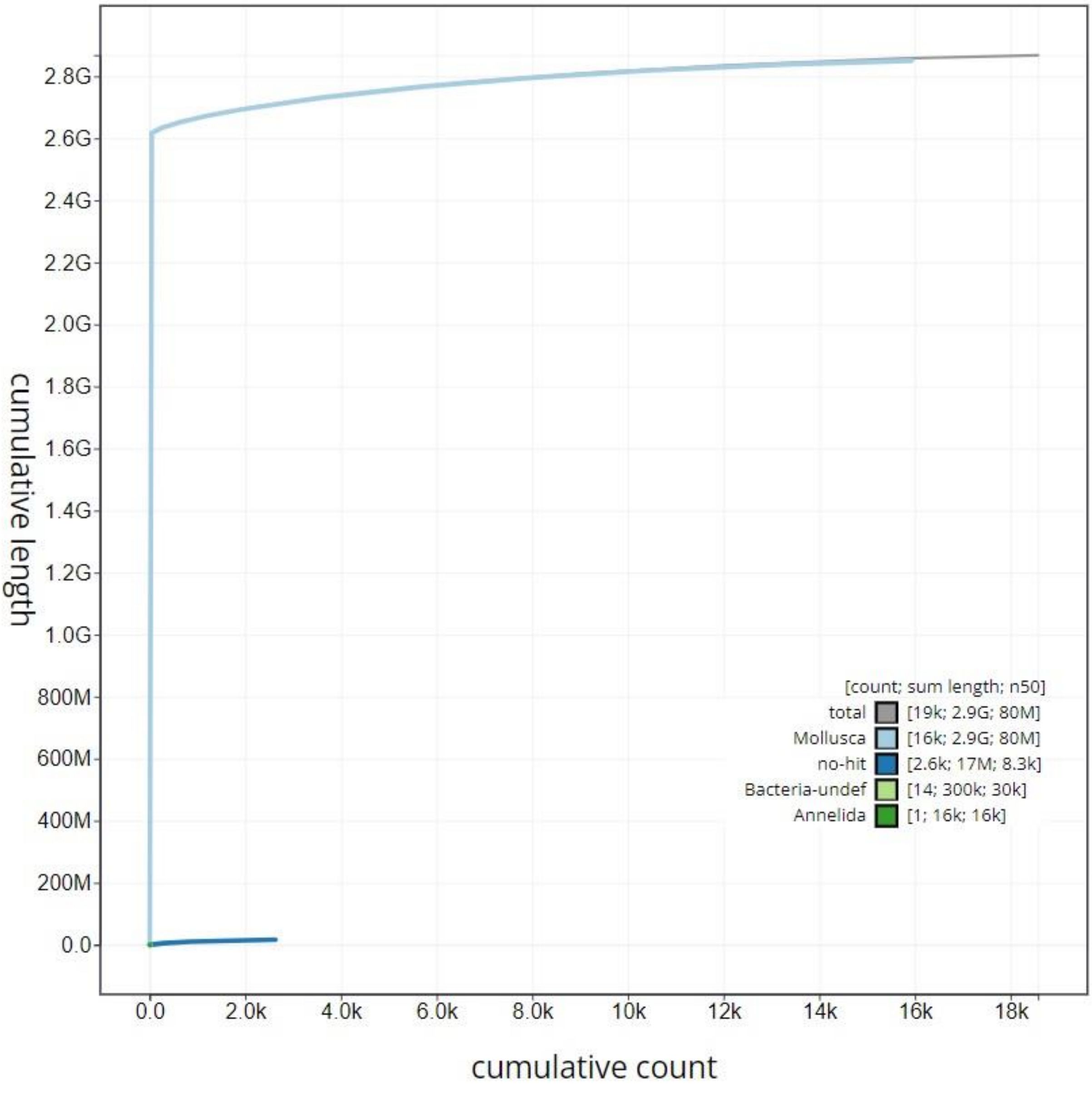

Cumulative sequence length for assembly B_results_blobtools. . The grey line shows cumulative length for all sequences. Coloured lines show cumulative lengths of sequences assigned to each phylum using the bestdistsum taxrule .

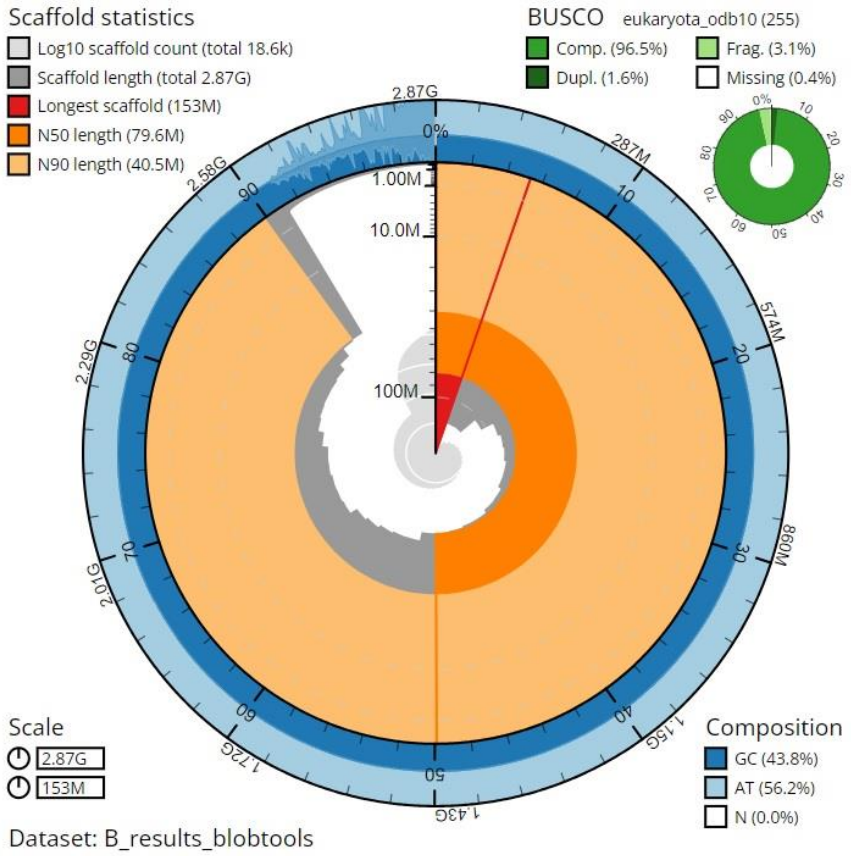

Snail plot summary of assembly statistics for assembly B_results_blobtools. The main plot is divided into 1,000 size-ordered bins around the circumference with each bin representing 0.1% of the 2.868.193.718 bp assembly. The distribution of sequence lengths is shown in dark grey with the plot radius scaled to the longest sequence present in the assembly (153.129.599 bp, shown in red). . Orange and pale-orange arcs show the N50 and N90 sequence lengths (79.645.777 and 40.485.963 bp), respectively. The pale grey spiral shows the cumulative sequence count on a log scale with white scale lines showing successive orders of magnitude. The blue and pale-blue area around the outside of the plot shows the distribution of GC, AT and N percentages in the same bins as the inner plot. A summary of complete, fragmented, duplicated and missing BUSCO genes in the eukaryota_odb10 set is shown in the top right .

eukaryota_odb10_busco		
BUSCOs	255	
Complete	246	96.5%
Duplicated	4	1.6%
Fragmented	8	3.1%
Missing	1	0.4%

More...

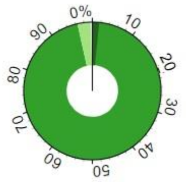

metazoa_odb10_busco		
BUSCOs	954	
Complete	892	93.5%
Duplicated	57	6.0%
Fragmented	36	3.8%
Missing	26	2.7%

More...

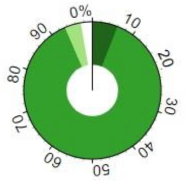

mollusca_odb10_busco		
BUSCOs	5295	
Complete	4076	77.0%
Duplicated	620	11.7%
Fragmented	244	4.6%
Missing	975	18.4%

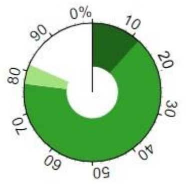

Fig. S6. BUSCO assessment of the available assembled genomes of Cone Snails. BUSCO v.5.1.3. and Metazoan Ortholog Database (odb10, n=954) were used.

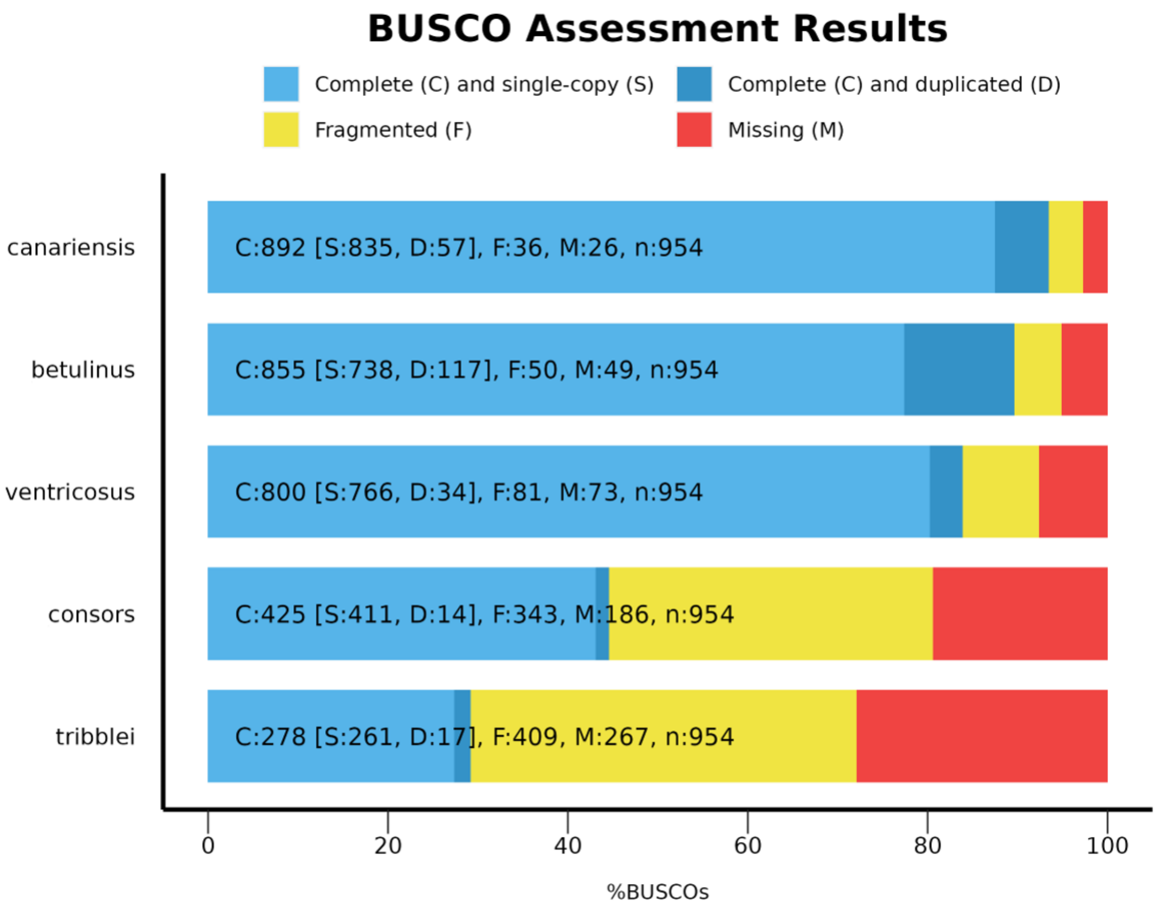

Supplementary Table S1. Sizes of homologous pseudo-chromosomes of *K.canariensis* and *L.ventricosus.*

Kc-Lv	K.canariensis		L.ventricosus		Size difference (%)
	Pschr	lenght (bp)	Pschr	lenght (bp)	
1-1	**1**	153129599	**1**	184223000	31093401 (16.88%)
2-2	**2**	120901141	**2**	148616974	27715833 (18.65%)
3-3	**3**	120357795	**3**	146199316	25841521 (17.68%)
4-4	**4**	103325145	**4**	124851017	21525872 (17.24%)
5-5	**5**	92224215	**5**	107110177	14885962 (13.90%)
6-6	**6**	89761154	**6**	106327600	16566446 (15.58%)
7-7	**7**	88671142	**7**	103955930	15284788 (14.70%)
8-10	**8**	87045814	**10**	101376596	14330782 (14.14%)
9-9	**9**	86932523	**9**	102508859	15576336 (15.20%)
10-11	**10**	84147650	**11**	100701486	16553836 (16.44%)
11-13	**11**	83805621	**13**	98613616	14807995 (15.02%)
12-15	**12**	81579722	**15**	96111069	14531347 (15.12%)
13-12	**13**	81536860	**12**	100273232	18736372 (18.69%)
14-16	**14**	80521922	**16**	93519712	12997790 (13.90%)
15-8	**15**	79653892	**8**	102716283	23062391 (22.45%)
16-14	**16**	79645777	**14**	96807691	17161914 (17.73%)
17-18	**17**	73358547	**18**	87461240	14102693 (16.12%)
18-17	**18**	73168961	**17**	88530480	15361519 (17.35%)
19-19	**19**	71984591	**19**	83799525	11814934 (14.10%)
20-20	**20**	69661957	**20**	82043315	12381358 (15.09%)
21-21	**21**	67758742	**21**	80309596	12550854 (15.63%)
22-24	**22**	62458667	**24**	70439404	7980737 (11.33%)
23-27	**23**	61759036	**27**	68587842	6828806 (9.96%)
24-22	**24**	61407306	**22**	75639241	14231935 (18.82%)
25-23	**25**	60340941	**23**	72024038	11683097 (16.22%)
26-25	**26**	60109553	**25**	69343152	9233599 (13.32%)
27-26	**27**	56353055	**26**	69102776	12749721 (18.45%)
28-29	**28**	52589416	**29**	61486793	8897377 (14.47%)
29-28	**29**	51623878	**28**	62680534	11056656 (17.64%)
30-32	**30**	51210485	**32**	56022305	4811820 (8.59%)
31-30	**31**	51007777	**30**	61155922	10148145 (16.59%)
32-33	**32**	50171869	**33**	53300255	3128386 (5.87%)
33-31	**33**	46304261	**31**	57586018	11281757 (19.59%)
34-35	**34**	41242764	**35**	43863197	2620433 (5.97%)
35-34	**35**	40485963	**34**	52697362	12211399 (23.17%)
**TOTAL SIZE**		**2616237741**		**3109985553**	

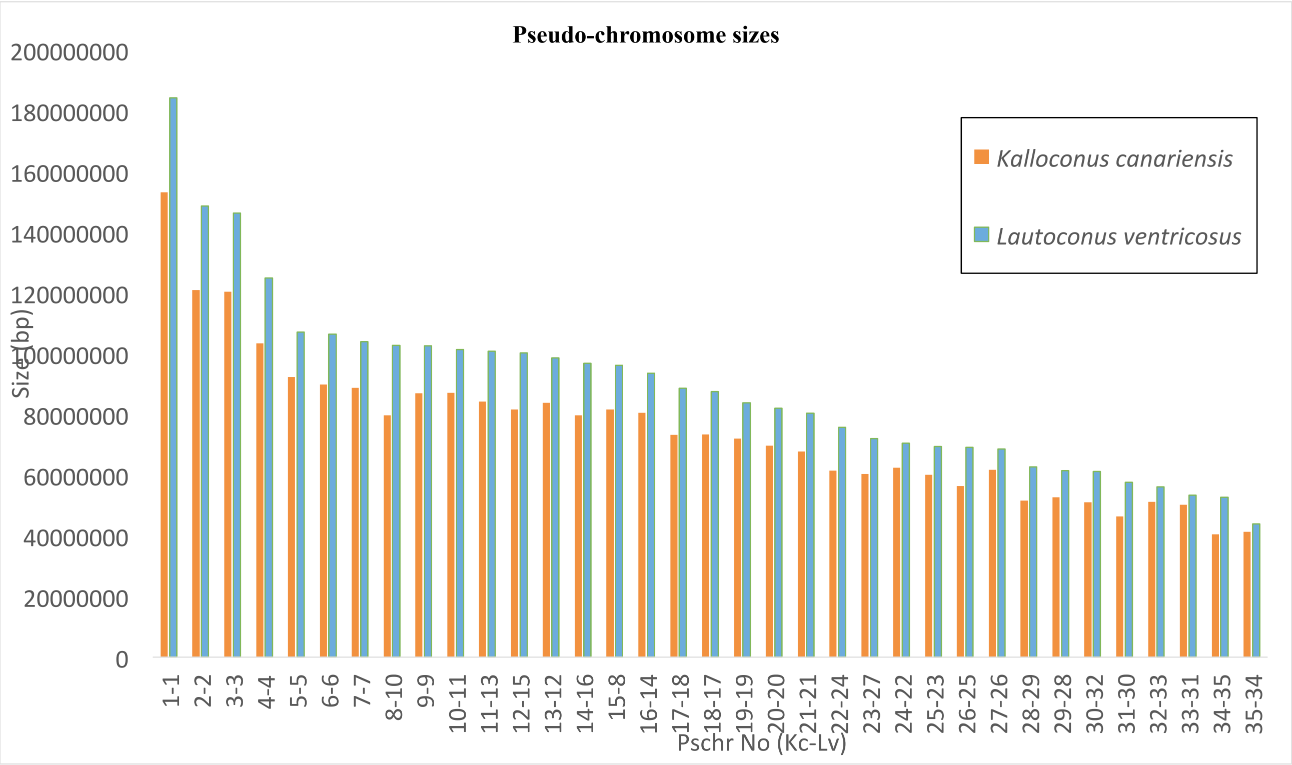

Supplementary Table S2. Comparison of number and length of genes and intergenics regions of homologous pseudo-chromosomes of *K.canariensis* (orange) and *L.ventricosus* (blue).

Kalloconus canariensis																		
**Pschr**	**TOTAL**	**1**	**2**	**3**	**4**	**5**	**6**	**7**	**8**	**9**	**10**	**11**	**12**	**13**	**14**	**15**	**16**	**17**
**No. genes**	**32508**	1716	1616	1341	1179	1056	1033	985	1057	1010	1124	1029	931	1070	1007	868	920	959
**No. Exons**	**123134**	7554	5959	5176	4673	3987	4232	3693	4290	3649	4409	3706	3712	3813	4102	3758	3485	3788
**exon/gene**	**3.79**	4.40	3.69	3.86	3.96	3.78	4.10	3.75	4.06	3.61	3.92	3.60	3.99	3.56	4.07	4.33	3.79	3.95
**exon length**	**36140900**	1981644	1646000	1420703	1372430	1221468	1191168	1091391	1238347	1068177	1241934	1171368	1111781	1105613	1124375	1008922	1034702	1134880
**intron length**	**101366538**	7357435	4755139	4492661	3873469	3504476	3847923	3095267	3513102	3130663	4041983	2898013	3086947	2930623	3657467	3613846	3025631	3307193
**gene length**	**137507438**	9339079	6401139	5913364	5245899	4725944	5039091	4186658	4751449	4198840	5283917	4069381	4198728	4036236	4781842	4622768	4060333	4442073
**% gene length**	**5.26**	6.10	5.29	4.91	5.08	5.12	5.61	4.72	5.46	4.83	6.28	4.86	5.15	4.95	5.94	5.80	5.10	6.06
**gene length average**	**4229.96**	5442.35	3961.10	4409.67	4449.45	4475.33	4878.11	4250.41	4495.22	4157.27	4700.99	3954.69	4509.91	3772.18	4748.60	5325.77	4413.41	4631.98
**intergenics length**	**2481569569**	143984792	114604177	114526032	98164232	87597939	84837649	84593776	82408000	83117942	78915921	79784942	77457044	77564183	75792602	75152666	75685845	69002269
**% intergenics length**	**94.85**	94.03	94.79	95.15	95.01	94.98	94.51	95.40	94.67	95.61	93.78	95.20	94.95	95.13	94.13	94.35	95.03	94.06
**Total Length**	**2616237741**	153129599	120901141	120357795	103325145	92224215	89761154	88671142	87045814	86932523	84147650	83805621	81579722	81536860	80521922	79653892	79645777	73358547
		**1**	**2**	**3**	**4**	**5**	**6**	**7**	**8-10**	**9-9**	**10-11**	**11-13**	**12-15**	**13-12**	**14-16**	**15-8**	**16-14**	**17-18**

Kalloconus canariensis																		
**Pschr**	**18**	**19**	**20**	**21**	**22**	**23**	**24**	**25**	**26**	**27**	**28**	**29**	**30**	**31**	**32**	**33**	**34**	**35**
**No. genes**	719	913	873	838	938	880	727	773	816	687	768	709	774	650	689	689	601	563
**No. Exons**	2832	3396	3355	3104	3115	3497	2924	3497	3009	2479	2565	2489	2298	2115	2144	2452	1948	1929
**exon/gene**	3.94	3.72	3.84	3.70	3.32	3.97	4.02	4.52	3.69	3.61	3.34	3.51	2.97	3.25	3.11	3.56	3.24	3.43
**exon length**	784175	1027717	995528	877129	1062331	1017689	822351	849034	908937	688143	823781	766216	823405	707859	766741	765695	685804	603462
**intron length**	2587341	2685354	2767368	2457077	2284006	3026366	2835445	2075875	2279431	2098079	1636536	1932151	1388023	1397776	1340379	1854951	1391476	1197066
**gene length**	3371516	3713071	3762896	3334206	3346337	4044055	3657796	2924909	3188368	2786222	2460317	2698367	2211428	2105635	2107120	2620646	2077280	1800528
**% gene length**	4.61	5.16	5.40	4.92	5.36	6.55	5.96	4.85	5.30	4.94	4.68	5.23	4.32	4.13	4.20	5.66	5.04	4.45
**gene length average**	4689.17	4066.89	4310.30	3978.77	3567.52	4595.52	5031.36	3783.84	3907.31	4055.64	3203.54	3805.88	2857.14	3239.44	3058.23	3803.55	3456.37	3198.10
**intergenics length**	69912444	68356011	65966472	64463957	59177295	57786068	57813106	57461440	56953610	53616572	50184746	48971185	49028445	48944662	48094704	43718398	39223134	38707309
**% intergenics length**	95.55	94.96	94.70	95.14	94.75	93.57	94.15	95.23	94.75	95.14	95.43	94.86	95.74	95.96	95.86	94.42	95.10	95.61
**Total Length**	73168961	71984591	69661957	67758742	62458667	61759036	61407306	60340941	60109553	56353055	52589416	51623878	51210485	51007777	50171869	46304261	41242764	40485963
	**18-17**	**19**	**20**	**21**	**22-24**	**23-27**	**24-22**	**25-23**	**26-25**	**27-26**	**28-29**	**29-28**	**30-32**	**31-30**	**32-33**	**33-31**	**34-35**	**35-34**

Lautoconus ventricosus																		
**Pschr**	**TOTAL**	**1**	**2**	**3**	**4**	**5**	**6**	**7**	**10**	**9**	**11**	**13**	**15**	**12**	**16**	**8**	**14**	**18**
**No. genes**	**29470**	1841	1527	1338	1150	935	1106	912	973	811	1065	867	921	968	1022	870	827	877
**No. exons**	**136886**	9863	7452	6176	5548	4298	5330	4307	4609	3753	5101	4299	4202	4460	4861	4261	3719	4014
**exon/gene**	**4.64**	5.36	4.88	4.62	4.82	4.60	4.82	4.72	4.74	4.63	4.79	4.96	4.56	4.61	4.76	4.90	4.50	4.58
**exon length**	**32851314**	2069736	1682244	1436248	1286828	1054001	1229560	1064710	1075204	924847	1195172	1060524	1016799	1067205	1135925	1025559	939180	991252
**intron length**	**175941477**	13002513	9667611	8016322	7136064	5757433	6481671	5806557	5800182	4848603	6719728	5727000	5637501	5738277	6042948	5978617	5070158	5059787
**gene length**	**208792791**	15072249	11349855	9452570	8422892	6811434	7711231	6871267	6875386	5773450	7914900	6787524	6654300	6805482	7178873	7004176	6009338	6051039
**% gene length**	**6.71**	8.18	7.64	6.47	6.75	6.36	7.25	6.61	6.78	5.63	7.86	6.88	6.92	6.79	7.68	6.82	6.21	6.92
**gene length average**	**7084.93**	8186.99	7432.78	7064.70	7324.25	7284.96	6972.18	7534.28	7066.17	7118.93	7431.83	7828.75	7225.08	7030.46	7024.34	8050.78	7266.43	6899.70
**intergenics length**	**2903065878**	169263183	137344058	136798648	116467331	100350913	98669604	97139991	94563729	96789715	92836940	91884263	89502758	93541433	86420489	95785883	90910533	81474923
**% intergenics length**	**93.35**	91.88	92.41	93.57	93.29	93.69	92.80	93.44	93.28	94.42	92.19	93.18	93.12	93.29	92.41	93.25	93.91	93.16
**Total Length**	**3109985553**	184223000	148616974	146199316	124851017	107110177	106327600	103955930	101376596	102508859	100701486	98613616	96111069	100273232	93519712	102716283	96807691	87461240

Lautoconus ventricosus																		
**Pschr**	**17**	**19**	**20**	**21**	**24**	**27**	**22**	**23**	**25**	**26**	**29**	**28**	**32**	**30**	**33**	**31**	**35**	**34**
**No. genes**	737	776	802	864	692	787	742	659	743	582	605	569	430	465	481	597	461	468
**No. exons**	3481	3490	3461	4074	3163	3710	3367	2835	3447	2552	2506	2343	1891	1964	1837	2607	1932	1973
**exon/gene**	4.72	4.50	4.32	4.72	4.57	4.71	4.54	4.30	4.64	4.38	4.14	4.12	4.40	4.22	3.82	4.37	4.19	4.22
**exon length**	820863	873942	864300	999009	755568	899788	771748	746803	826418	596585	639363	589039	504564	544862	526388	641243	502738	493099
**intron length**	4658865	4118786	4415040	5353195	3863781	4515899	4202958	3761573	4344599	3482602	3048128	3068941	2022766	2432510	2223182	3162478	2392065	2383137
**gene length**	5479728	4992728	5279340	6352204	4619349	5415687	4974706	4508376	5171017	4079187	3687491	3657980	2527330	2977372	2749570	3803721	2894803	2876236
**% gene length**	6.19	5.96	6.43	7.91	6.56	7.90	6.58	6.26	7.46	5.90	6.00	5.84	4.51	4.87	5.16	6.61	6.60	5.46
**gene length average**	7435.18	6433.93	6582.72	7352.09	6675.36	6881.43	6704.46	6841.24	6959.65	7008.91	6095.03	6428.79	5877.51	6402.95	5716.36	6371.39	6279.40	6145.80
**intergenics length**	83097812	78860916	76848814	74013439	65888709	63197960	70689439	67579692	64205383	65059857	57829889	59041441	53532347	58198189	50586273	53832217	41016503	49842604
**% intergenics length**	93.86	94.11	93.67	92.16	93.54	92.14	93.46	93.83	92.59	94.15	94.05	94.19	95.56	95.16	94.91	93.48	93.51	94.58
**Total Length**	88530480	83799525	82043315	80309596	70439404	68587842	75639241	72024038	69343152	69102776	61486793	62680534	56022305	61155922	53300255	57586018	43863197	52697362

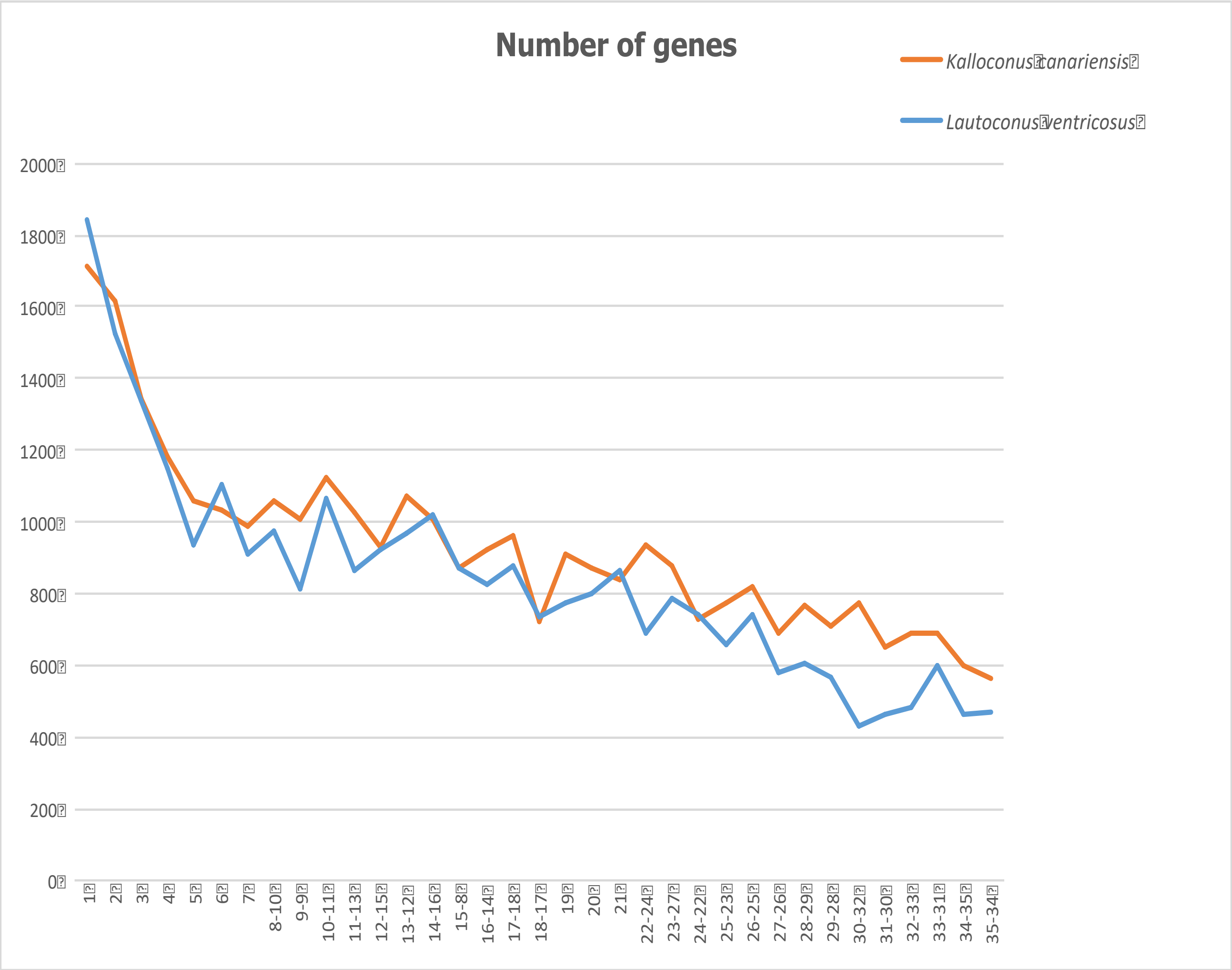
Number of genes
Kalloconus canariensis
Lautoconus ventricosus
2000
1800
1600
1400
1200
1000
800
600
400
200
0
1
2
3
4
5
6
7
8-10
9-9
10-11
11-13
12-15
13-12
14-16
15-8
16-14
17-18
18-17
19
20
21
22-24
23-27
24-22
25-23
26-25
27-26
28-29
29-28
30-32
31-30
32-33
33-31
34-35
35-34

Supplementary Table S3. Taxonomy of gastropods and source of sequences included in phylogenomic analyses

Gastropoda			
**Group (Subclass/Order/Superfamily)***	**Species**	**Author and year**	**Acc. No. (NCBI-SRA)**
Heterobranchia/Nudibranchia/Fionoidea	*Fiona pinnata*	Eschscholtz 1831	SRR1505109
Neritimorpha/Cycloneritida/Nerioidea	*Nerita melanotragus*	Castro and Colgan 2010	SRR1920139
Caenogastropoda/Architaenioglossa/Ampallarioidea	*Pomacea canaliculata*	Lamarck, 1828	SRR7454777
Caenogastropoda/unassigned/Cerithioidea	*Semisulcospira coreana*	Martens, 1886	SRR7160963
Caenogastropoda/Littorinimorpha/Truncatelloidea	*Bithynia siamensis goniomphalos*	Morelet, 1866	SRR768418
Caenogastropoda/Littorinimorpha/Truncatelloidea	*Oncomelania hupensis*	Gredler, 1881	SRR5450825
Caenogastropoda/unassigned/Abyssochrysoidea	*Rubyspira osteovora*	Johnson et al. 2010	SRR1505136
Caenogastropoda/Littorinimorpha/Littorinoidea	*Echinolittorina malaccana*	Philippi, 1847	SRR1269556
Caenogastropoda/Littorinimorpha/Naticoidea	*Neverita didyma*	Roding, 1798	SRR8472156
Caenogastropoda/Littorinimorpha/Naticoidea	*Euspira heros*	Say, 1822	SRR1505131
Caenogastropoda/unassigned/Epitonioidea	*Janthina janthina*	Linnaeus, 1758	SRR1505114
Caenogastropoda/Littorinimorpha/Stromboidea	*Strombus gigas*	Linnaeus, 1758	SRR827578
Caenogastropoda/Littorinimorpha/Velutinoidea	*Marseniopsis mollis*	Smith, 1902	SRR3205287
Caenogastropoda/Neogastropoda/Muricoidea	*Dicathais orbita*	Gmelin, 1791	ERR1198126
Caenogastropoda/Neogastropoda/Buccinoidea	*Colubraria reticulata*	Blainville, 1826	ERR852087
Caenogastropoda/Neogastropoda/Conoidea	*Crassispira cerithina*	Anton, 1838	SRR1574922
Caenogastropoda/Neogastropoda/Conoidea	*Gemmula speciosa*	Reeve, 1842	SRR1574907
Caenogastropoda/Neogastropoda/Conoidea	*Dendroconus betulinus*	Linnaeus, 1758	SRR2124878
Caenogastropoda/Neogastropoda/Conoidea	*Kalloconus canariensis*	Tenorio et al. 2020	This work
Caenogastropoda/Neogastropoda/Conoidea	*Lautoconus ventricosus*	Gmelin, 1791	Pardos-Blas et al. 2021

*based on WoRMS (https://www.marinespecies.org/)

Supplementary Table S4. Genome assembly and BUSCO metrics of genome annotations of molluscs available at NCBI (https://www.ncbi.nlm.nih.gov/).

Class*	Subclass*	Organism Name	Assembly Level	Size	Contig N50	Gene Count	BUSCO metrics metazoa_odb10 (n:954)	BUSCO metrics eukaryota_odb10 (n=255)	BioProject
Gastropoda	Caenogastropoda	*Kalloconus canariensis*	Scaffold	2868115334	79645777	34241	C:90.9%[S:82.3%,D:8.6%],F:6.1%,M:3.0%	C:87.4%[S:83.5%,D:3.9%],F:10.2%,M:2.4%	PRJNA843968
Gastropoda	Caenogastropoda	*Lautoconus ventricosus*	Scaffold	3591508080	93519712	32675	C:32.2%[S:31.6%,D:0.6%],F:22.7%,M:45.1%	C:36.9%[S:36.9%,D:0.0%],F:28.6%,M:34.5%	PRJNA678883
Gastropoda	Caenogastropoda	*Batillaria attramentaria*	Contig	717569005	1290776	29795	C:42.5%[S:41.1%,D:1.4%],F:22.1%,M:35.4%	C:40.8%[S:40.4%,D:0.4%],F:31.0%,M:28.2%	PRJNA640962
Gastropoda	Caenogastropoda	*Pomacea canaliculata*	Chromosome	440143917	1072857	24345	C:98.2%[S:74.9%,D:23.3%],F:0.4%,M:1.4%	C:100.0%[S:79.6%,D:20.4%],F:0.0%,M:0.0%	PRJNA427478
Gastropoda	Heteropbranchia	*Achatina fulica*	Chromosome	1855883074	59589303	22858	C:59.7%[S:55.9%,D:3.8%],F:3.4%,M:36.9%	C:57.3%[S:54.9%,D:2.4%],F:6.7%,M:36.0%	PRJNA511624
Gastropoda	Heteropbranchia	*Aplysia californica*	Scaffold	927296314	9584	21514	C:97.8%[S:75.8%,D:22.0%],F:0.8%,M:1.4%	C:98.4%[S:75.3%,D:23.1%],F:0.8%,M:0.8%	PRJNA13635
Gastropoda	Heteropbranchia	*Biomphalaria glabrata*	Scaffold	916374414	7298	30340	C:88.9%[S:66.7%,D:22.2%],F:6.4%,M:4.7%	C:86.3%[S:67.5%,D:18.8%],F:10.2%,M:3.5%	PRJNA12879
Gastropoda	Heteropbranchia	*Bulinus truncatus*	Scaffold	1221776979	234305	26279	C:94.9%[S:77.3%,D:17.6%],F:2.7%,M:2,4%	C:95.3%[S:75.3%,D:20.0%],F:3.5%,M:1.2%	PRJNA680620
Gastropoda	Heteropbranchia	*Candidula unifasciata*	Scaffold	1356663438	246413	22464	C:90.8%[S:84.1%,D:6.7%],F:4.7%,M:4.5%	C:89.5%[S:87.1%,D:2.4%],F:5.1%,M:5.4%	PRJEB41346
Gastropoda	Heteropbranchia	*Elysia chlorotica*	Scaffold	557480303	30474	23871	C:91.5%[S:91.2%,D:0.3%],F:3.8%,M:4.7%	C:93.0%[S:92.2%,D:0.8%],F:3.1%,M:3.9%	PRJNA484060
Gastropoda	Heteropbranchia	*Elysia marginata*	Scaffold	790322091	6205	70752	C:86.1%[S:85.3%,D:0.8%],F:9.1%,M:4.8%	C:86.7%[S:85.5%,D:1.2%],F:9.8%,M:3.5%	PRJDB3267
Gastropoda	Heteropbranchia	*Plakobranchus ocellatus*	Scaffold	927888823	5479	77230	C:91.1%[S:89.9%,D:1.2%],F:6.2%,M:2.7%	C:88.6%[S:87.8%,D:0.8%],F:9.4%,M:2.0%	PRJDB3267
Gastropoda	Heteropbranchia	*Siphonaria sp. JEL0065*	Contig	37595823	21877	12956	C:49.6%[S:41.8%,D:7.8%],F:6.1%,M:44.3%	C:74.5%[S:64.3%,D:10.2%],F:7.5%,M:18.0%	PRJNA619581
Gastropoda	Neomphaliones	*Chrysomallon squamiferum*	Chromosome	404615235	1883489	16917	C:83.8%[S:82.9%,D:0.9%],F:6.3%,M:9.9%	C:86.3%[S:85.5%,D:0.8%],F:7.5%,M:6.2%	PRJNA523462
Gastropoda	Neomphaliones	*Gigantopelta aegis*	Chromosome	1149605642	461769	26793	C:98.5%[S:70.5%,D:28.0%],F:0.8%,M:0.7%	C:100.0%[S:66.3%,D:33.7%],F:0.0%,M:0.0%	PRJNA612619
Gastropoda	Patellogastropoda	*Lottia gigantea*	Scaffold	359505668	96027	23827	C:96.5%[S:95.8%,D:0.7%],F:1.5%,M:2.0%	C:96.5%[S:95.7%,D:0.8%],F:2.4%,M:1.1%	PRJNA175706
Gastropoda	Patellogastropoda	*Patella vulgata*	Chromosome	694468775	18376887	25085	C:90.5%[S:69.3%,D:21.2%],F:1.3%,M:8.2%	C:92.5%[S:72.5%,D:20.0%],F:2.0%,M:5.5%	PRJEB51188
Gastropoda	Vetigastropoda	*Haliotis rubra*	Scaffold	1378265264	1177711	39947	C:97.5%[S:77.7%,D:19.8%],F:1.5%,M:1.0%	C:97.2%[S:78.4%,D:18.8%],F:2.0%,M:0.8%	PRJNA489521
Gastropoda	Vetigastropoda	*Haliotis rufescens*	Scaffold	1334454214	8868657	42838	C:99.4%[S:74.7%,D:24.7%],F:0.0%,M:0.6%	C:99.6%[S:74.9%,D:24.7%],F:0.0%,M:0.4%	PRJNA26275698
Bivalvia	Autobranchia	*Crassostrea angulata*	Chromosome	624273838	10870631	38153	C:98.9%[S:76.4%,D:22.5%],F:0.4%,M:0.7%	C:99.2%[S:81.2%,D:18.0%],F:0.0%,M:0.8%	PRJNA857752
Bivalvia	Autobranchia	*Crassostrea gigas*	Chromosome	647887097	1564469	38296	C:98.5%[S:67.6%,D:30.9%],F:0.3%,M:1.2%	C:100.0%[S:64.7%,D:35.3%],F:0.0%,M:0.0%	PRJEB35351
Bivalvia	Autobranchia	*Crassostrea virginica*	Chromosome	684723884	1971208	39505	C:98.1%[S:58.6%,D:39.5%],F:0.3%,M:1.6%	C:98.0%[S:54.5%,D:43.5%],F:0.8%,M:1.2%	PRJNA376014
Bivalvia	Autobranchia	*Dreissena polymorpha*	Chromosome	1797942340	1111654	51549	C:86.9%[S:75.1%,D:11.8%],F:6.4%,M:6.7%	C:85.1%[S:76.1%,D:9.0%],F:7.8%,M:7.1%	PRJNA533175
Bivalvia	Autobranchia	*Mercenaria mercenaria*	Chromosome	1858199728	82432825	45375	C:96.0%[S:66.4%,D:29.6%],F:1.2%,M:2.8%	C:94.1%[S:70.2%,D:23.9%],F:1.6%,M:4.3%	PRJNA638823
Bivalvia	Autobranchia	*Mizuhopecten yessoensis*	Scaffold	987568220	65014	27323	C:98.6%[S:75.2%,D:23.4%],F:0.4%,M:1.0%	C:99.2%[S:80.4%,D:18.8%],F:0.4%,M:0.4%	PRJNA259405
Bivalvia	Autobranchia	*Mya arenaria*	Chromosome	1216068291	3381420	44373	C:98.5%[S:63.1%,D:35.4%],F:0.4%,M:1.1%	C:99.6%[S:65.5%,D:34.1%],F:0.0%,M:0.4%	PRJNA874712
Bivalvia	Autobranchia	*Mytilus californianus*	Scaffold	1651950171	16323199	41296	C:96.2%[S:72.5%,D:23.7%],F:0.4%,M:3.4%	C:98.0%[S:74.5%,D:23.5%],F:0.0%,M:2.0%	PRJNA796333
Bivalvia	Autobranchia	*Mytilus coruscus*	Scaffold	1903825920	817337	58540	C:80.7%[S:68.3%,D:12.4%],F:4.4%,M:14.9%	C:81.2%[S:70.6%,D:10.6%],F:6.7%,M:12.1%	PRJEB33342
Bivalvia	Autobranchia	*Mytilus edulis*	Scaffold	1827085763	511485	69378	C:83.7%[S:64.5%,D:19.2%],F:5.2%,M:11.1%	C:86.3%[S:65.5%,D:20.8%],F:6.3%,M:7.4%	PRJEB38403
Bivalvia	Autobranchia	*Mytilus galloprovincialis*	Scaffold	1500149602	2627	3184	C:2.8%[S:2.8%,D:0.0%],F:3.2%,M:94.0%	C:2.4%[S:2.4%,D:0.0%],F:6.7%,M:90.9%	PRJNA262617
Bivalvia	Autobranchia	*Ostrea edulis*	Scaffold	935138052	1809825	38564	C:96.8%[S:65.0%,D:31.8%],F:0.5%,M:2.7%	C:98.1%[S:62.4%,D:35.7%],F:0.4%,M:1.5%	PRJNA772111
Bivalvia	Autobranchia	*Pecten maximus*	Chromosome	918306378	1258799	30903	C:98.5%[S:74.7%,D:23.8%],F:0.4%,M:1.1%	C:98.9%[S:77.3%,D:21.6%],F:0.4%,M:0.7%	PRJEB35330
Cephalopoda	Coleoidea	*Octopus bimaculoides*	Chromosome	2342518435	5523	20144	C:95.0%[S:69.6%,D:25.4%],F:2.5%,M:2.5%	C:95.7%[S:70.6%,D:25.1%],F:3.1%,M:1.2%	PRJNA270931
Cephalopoda	Coleoidea	*Octopus sinensis*	Chromosome	2719136158	490217	29784	C:98.4%[S:69.2%,D:29.2%],F:0.8%,M:0.8%	C:98.1%[S:70.6%,D:27.5%],F:0.8%,M:1.1%	PRJNA541812
Cephalopoda	Coleoidea	*Sepia pharaonis*	Contig	4785531890	1926397	81669	C:71.5%[S:66.0%,D:5.5%],F:7.3%,M:21.2%	C:76.9%[S:70.6%,D:6.3%],F:7.5%,M:15.6%	PRJEB33343

*based on WoRMS (https://www.marinespecies.org/)

Supplementary Methods. Whole-genome assembly, scaffolding, and annotation of *Kalloconus canariensis.*

Ana Herráez-Perez, José Ramón Pardos-Blas, Carlos M.L. Afonso, Manuel J. Tenorio and Rafael Zardoya

DNA extraction, library preparation and sequencing

DNA extraction, library preparation, sequencing, assembly, scaffolding, and annotation were performed by Dovetail Genomics (Scotts Valley, CA, USA). HMW DNA was isolated from muscle foot samples with Genomic-tip 20G (Qiagen, Toronto, ON, Canada) columns and was quantified using Qubit 2.0 Fluometer (Life Technologies, Carlsbad, CA, USA). The quality of DNA extraction was assessed by gel electrophoresis. PacBio SMRTbell libraries (>20 kb) were generated and sequencing was performed on a PacBio Sequel II using the CLR mode.

For the Omni-C library preparation, the chromatin was fixed with formaldehyde in the nucleus. The cross-linked chromatin was then digested in situ with DNase I [1]. Following digestion, the cells were lysed with SDS to extract the chromatin fragments and the chromatin fragments were bound to Chromatin Capture Beads. Next, the chromatin ends were repaired and ligated to a biotinylated bridge adapter followed by proximity ligation of adapter-containing ends. After proximity ligation, the crosslinks were reversed, the associated proteins were degraded, and the DNA was purified then converted into a sequencing library using Illumina-compatible adaptors. Biotin-containing fragments were isolated using streptavidin beads prior to PCR amplification.

Genome assembly, scaffolding and quality evaluation

The data from the *de novo* genome assembly and the OmniC library (Mapping Quality score, MQ>50 reads) were used for assembly using HiRiSE^{TM}, a software pipeline designed specifically for using proximity ligation data to scaffold genome assemblies [2]. OmniC library

sequences were aligned to the draft input assembly using BWA (BWA, RRID:SCR_010910) [3]. The separations of OmniC read pairs mapped within draft scaffolds were analyzed by HiRiSE™ to produce a likelihood model for genomic distance between read pairs, and the model was used to identify and break putative misjoins, to score prospective joins, and finally, make joins above a threshold.

Genome annotation

RNA extraction: foot samples of three cone snails (TF36, TF37, and TF39) plus venom glands of two more individuals (TF42 and TF43) were Illumina sequenced. For RNA extraction, each tissue was incubated in TRIzol LS reagent (Thermo Fisher Scientific, Waltham, MA, USA) and ground with ceramic beads in a Precellys Evolution tissue homogenizer. The upper aqueous phase with RNA was separated from lower phenol-chloroform phase by adding chloroform and centrifuging at 12,000g for 15 min at 4ºC. Then, RNA was precipitated in 1 volume of isopropanol and incubated overnight at −80ºC. The Direct-zol RNA miniprep kit (Zymo Research, Irvine, CA, USA) was used to purify total RNA following manufacturer's instructions.

Repeat families were identified *de novo* and classified using RepeatModeler (RepeatModeler, RRID:SCR_015027) v2.0.1. [4], which depends on two *de novo* repeat finding programs, RECON v1.08 [5] and RepeatScout v1.0.6 [6] to identify repetitive elements within the genome. The custom repeat library was used to discover, identify and mask the repeats in the assembly file using RepeatMasker (RepeatMasker, RRID:SCR_012954) v4.1.0 [7]. Coding sequences from *Kalloconus trochulus* (SRR11807506), *Pionoconus consors* (SRR1958882, SRR1958824, SRR1954996, SRR1958823, SRR1964034 and SRR1955039), *Lautoconus ventricosus* (http://dx.doi.org/10.5524/100892) and the RNA-seq raw reads generated in this work (TF36, TF37, and TF39 foot tissues plus TF42 and TF43 venom glands) were used to train the initial *ab initio* gene model for *K. canariensis* using AUGUSTUS (Augustus, RRID:SCR_008417) v2.5.5 [8] and six rounds of prediction optimization were performed. The same coding sequences were also used to train a separate *ab initio* gene model using SNAP

v2006-07-28 [9]. RNA-seq reads were mapped onto the genome using the STAR (STAR, RRID:SCR_004463) v2.7 aligner [10] and intron hints were generated with the bam2hints tools within AUGUSTUS. Then, SNAP and AUGUSTUS (with intron-exon boundary hints provided from RNA-Seq) were used to predict genes in the repeat-masked reference genome with the MAKER (MAKER, RRID:SCR_005309) v3.01.01 pipeline [11].

To guide the prediction process, Swiss-Prot peptide sequences from the UniProt database were downloaded and used in conjunction with the protein sequences from mollusks used to generate peptide evidence in the Maker pipeline. Only genes that were predicted by both SNAP and AUGUSTUS were retained in the final gene sets. To help assess the quality of the gene prediction, Annotation Edit Distance (AED) scores were generated for each of the predicted genes as part of the MAKER pipeline. Genes were further characterized for their putative function by performing a BLASTP search of the peptide sequences against the UniProt database. In addition, tRNAs were predicted using the software tRNAscan-SE (tRNAscan-SE, RRID:SCR_010835) v2.05 [12].

References

1. Ramani V, Cusanovich DA, Hause RJ, et al. Mapping 3D genome architecture through in situ DNase Hi-C. *Nat Protoc.* ; 2016;**11**:2104-2121.
2. Putnam NH, O'Connell BL, Stites JC, et al. Chromosome scale shotgun assembly using an in vitro method for long-range linkage. *Genome Res*. 2016;**26**:342–50.
3. Li H. Aligning sequence reads, clone sequences and assembly contigs with BWA-MEM. *arXiv preprint*. 2013, *arXiv*:1303.3997v2 [q-bio.GN].
4. Flynn JM, Hubley R, Goubert C, et al. RepeatModeler2 for automated genomic discovery of transposable element families. *Proc Natl Acad Sci USA*. 2020;**117**(17):9451–7
5. Bao Z, Eddy SR. Automated de novo identification of repeat sequence families in sequenced genomes. *Genome Res*. 2002.;**12**:1269–76.
6. Price AL, Jones NC, Pevzner PA. De novo identification of repeat families in large genomes. *Bioinformatics*. 2005;**21**:i351–8.
7. Smit AFA, Hubley R, Green P. RepeatMasker Open-4.1.0. 2019-2020. Available from: http://www.repeatmasker.org/.
8. Stanke M, Steinkamp R, Waack S, et al. AUGUSTUS: a web server for gene finding in eukaryotes. *Nucleic Acids Res*. 2005;**32**(Suppl. 2):W309–12.
9. Korf I. Gene finding in novel Genomes. *BMC Bioinformatics*. 2004;**5**:59
10. Dobin A, Davis CA, Schlesinger F, et al. STAR: ultrafast universal RNA-seq aligner. *Bioinformatics*. 2013;**29**:15–21.
11. Holt C, Yandell M. MAKER2: an annotation pipeline and genome-database management tool for second-generation genome projects. *BMC Bioinformatics*. 2011;**12**:491.
12. Chan PP, Lowe TM. tRNAscan-SE: searching for tRNA genes in genomic sequences. *Methods Mol Biol*. 2019;**1962**:1–14.
