# Supplementary material for: Chromosome-level genome of the venomous snail Kalloconus canariensis: a valuable model for venomics and comparative genomics
Source: Gigascience. 2023 Sep 30;12:giad075. doi: 10.1093/gigascience/giad075 (PMC10541794; doi:10.1093/gigascience/giad075)
Supplement: giad075_Response_to_Reviewer_Comments_Original_Submission [file giad075_response_to_reviewer_comments_original_submission.pdf]

Author's Response To Reviewer Comments

Response to Reviewers

Reviewer #1:
The paper by Ana Herráez-Pérez et al. provides a high quality genome of a new Conus species. All research appears to be conducted appropriately except the authors used SPAdes for de novo transcriptome assembly tool, which has inferior performance to Trinity, particularly in venom/toxins encoding species.

We appreciate all reviewer comments and positive feedback. Regarding the choice of de novo transcriptome assembly software, comparative analyses of different assembly methods showed that rnaSPAdes does not present remarkable differences regarding quality annotation compared to Trinity (Bushmanova et al. 2019; GigaScience 8, giz100). Nonetheless, in order to address the referee suggestion, we have assembled the transcriptomes with Trinity and repeated the phylogenomic analysis, obtaining consistent results. The new phylogenomic tree has been incorporated into the article.

The authors also report using these transcriptomes to guide genome assembly but the sequences identified in the venom gland transcriptome need to be presented in the paper and discussed as part of the paper. An explanation for the lack of any venom genomes also needs to be provided, or if these were identified they need to be included in the manuscript.

We obtained RNA-seq data from three muscle (foot) and two venom glands samples of Kalloconus canariensis. These raw reads together with those from other cone snails available at SRA were used to train ab initio models for annotation. This has been clarified in the main text and in supplementary materials.
This is a Data Note, not a full research paper. Thus, we focus on describing the gene content of K. canariensis but cannot get into an in-depth analysis of venom genes and synteny comparisons with other cone snail genomes, which is the subject of a coming paper.

Reviewer #2:
The authors of the manuscript entitled "Chromosome-Level Genome of the Venomous Snail Kalloconus canariensis: A Valuable Model for Venomics and Comparative Genomics" provided the first reference genome for the species based on PacBio CLR long reads and Omni-C data. The genome presented in this work, showed very high standards in terms of contiguity (chromosome level) and completeness. Overall, the manuscript is well explained and data generated will be very useful, not only for researchers interested in this group of molluscs, but any interested in venomics.

We thank the reviewer for the kind words and the constructive suggestions.

However, I have a couple of issues that in my opinion should be addressed before the acceptance of the manuscript. First of all, I would strongly recommend to include some kind of contamination analysis (e.g. blobtools https://github.com/DRL/blobtools). It is something fast and easy to run and could discard any contings-sccaffolds that come from other organisms. I don't think that data here presented showed any problem of contamination, but I think is an approach that should always be done to check the quality of raw data.

Following the suggestion of the referee we checked potential contaminations with BlobToolKit. In the revised version, we describe this analysis in the main text and include the blobplot as supplementary figure S5.

My second major concern is related to annotation quality, that showed a 13% of missing BUSCOs. The high contiguity of the genome here provided, with very good completeness (only 2.7% of missing BUSCOs), makes me think that the annotation could be improved. This idea is also supported by the use of an old version of Augustus (v2.5.5.), because since many time ago the version 3.0 is available and it has showed to recover better predictions. In any case, I would recommend to authors to try another annotation

pipelines such as GeMoMa, EVidenceModeller or BRAKER2 to try to recover a better annotation scheme.

Following the suggestion of the reviewer, we ran an alternative annotation with BRAKER2. This is now mentioned in the ‘genome annotation’ section of Materials & Methods (pages 5-6, new lines 24-5). New results and discussion are also included in page 9, lines 7-22.

I also have attached some minor comments in the pdf version of the main manuscript and supplementary material that could help to improve the manuscript:
Here I would be more precise saying something like: "Their genomes sizes are comparable and range from 2.87 to 3.59 Gb". This difference of 700 Mb is important since there are many species (including molluscks, e.g. Chrysomallon squamiferum) that they have genome sizes within this value.

We agree with the reviewer. Changed as suggested in page 3, new lines 18-19.

You could also say "we collected the specimens off the coast of Tenerife" to be more specific about the location. Giving geographical coordinates could also be helpful in providing precise information about where the specimens were collected.

Page 3, new lines 24-25 "...were collected in Playa de Porís, Tenerife, Canary Islands (Spain; GPS coordinates: 28.16447, -16.43185) in September 2020."

Remove and

Removed! (page 4, line 3).

I consider this step fundamental. Did you provide any estimated genome size? any other parameter? if not indicate that was run under default parameters.

As suggested, the clarification is now added in page 4, lines 21-22 "using wtdgb2 v.2.5 with default parameters."

Why did you select directly this tool? Did you check any other long-reads assemblers such as flye or canu?

As the reviewer mention, there are multiple alternatives for genome assembly based on long reads. Wtdgb2 was chosen because it provides faster assembly speeds for large genomes with comparable contiguity and assembly accuracy and tend to have less duplicates (Ruan & Li, 2020; Nat Methods 2020 17, 155-158), making it a good choice for our genome. Besides that, it provided us with excellent results when assembling the genome of Lautoconus ventricosus (the reasons for choosing Wtdgb2 are now mentioned in the main text (page 4, lines 22-23).

In my opinion, the approach used for transcriptome assembly is not clear. Did you combine all foot and venom glands from the 5 specimens together to obtain two transcriptomes (foot and venom)? or did you assembled all transcriptomes independently?

Actually, the reviewer is right and the procedure was not well explained. We use all raw reads from three muscle (foot) and two venom glands to directly guide the genome annotation with both MAKER and BRAKER2. Thus, we have modified the statement accordingly (page 5, lines 7-8). "RNA-sequence reads of three muscle (foot) and two venom glands of K. canariensis were pooled together and used for gene annotation."

This is a pretty old version of Augustus (March 2012)... could be the reason why you have this low BUSCO values in the annotation? I strongly recommend to use and updated version of augustus (3.4; 3.5…)

Following the suggestion of the reviewer, we updated to v3.5.0 of Augustus and reran the annotation step. The new assembly annotation retrieves 90.9% of complete genes, 6.1 % of fragmented genes and 3.0% of missing genes. The main text of the paper has been updated accordingly.

I think is not clear how you assembled transcriptomes. See my previous comment.

Following the advice of the reviewer, we have clarified the procedure that we followed for the assembly of the transcriptomes that were used for phylogenomic reconstruction. We now explain in the M&M section: "RNA-seq raw reads retrieved from SRA NCBI were assembled using Trinity v2.12.0 to generate the transcriptome of each of the 18 species."

This model was obtained with IQ-TREE or did you use other tool such as PartitionFinder or jModelTest?

We now explain in page 7, lines 20-22 "The ModelFinder module was used to select LG+R4 as the best-fit model, according to the Bayesian Information Criterion (BIC)."

Protein Coding Genes?

Changed by “genes” in page 9, line 12

I would say by aligning the translated proteins against the genome assembly

This statement was deleted, as we made a new genome annotation recovering a higher percentage of genes.

Taking into account that you recovered a high-quality genome assembly, I found this value really low. In the table S4 that you provide, not many species have 10% of missing BUSCOs (and two that have that values are at contig level).

We agree with the reviewer. The new annotation has a lower percentage (3%) of missing BUSCOs. The text has been changed accordingly.

It is true but the only reference at chormosome level has 100% of BUSCO. There are other gastropoda genomes assemblies in other databases at chromosome level. It would be interesting if it something really extraordinary of Pomacea. Chrysomallon squamiferum:
https://datadryad.org/stash/dataset/doi:10.5061/dryad.24053dn; Achatina fulica:
http://gigadb.org/dataset/100647

We agree with the reviewer, now we include in table S4 the BUSCOs for the proteins of Chrysomallon squamiferum (86.3% with eukaryota odb10 and 83.8% with metazoa odb10) and Achatina fulica (57.3% with eukaryota odb10 and 59.7% with metazoa odb10).

This value could indicate that annotation is not as good as expected...
As we mentioned before, these values have been improved with the new annotation. We now included it in Table 1.
