# Supplementary material for: Chromosome-level genome of the venomous snail Kalloconus canariensis: a valuable model for venomics and comparative genomics
Source: Gigascience. 2023 Sep 30;12:giad075. doi: 10.1093/gigascience/giad075 (PMC10541794; doi:10.1093/gigascience/giad075)
Supplement: giad075_Reviewer_2_Report_Original_Submission — Luis Javier Chueca, PhD -- 3/21/2023 Reviewed [file giad075_reviewer_2_report_original_submission.pdf]

Reviewer Report

Title: Chromosome-Level Genome of the Venomous Snail Kalloconus canariensis: A Valuable Model for Venomics and Comparative Genomics

Version: Original Submission **Date:** 3/21/2023

Reviewer name: Luis Javier Chueca, PhD

Reviewer Comments to Author:

The authors of the manuscript entitled "Chromosome-Level Genome of the Venomous Snail Kalloconus canariensis: A Valuable Model for Venomics and Comparative Genomics" provided the first reference genome for the species based on PacBio CLR long reads and Omni-C data. The genome presented in this work, showed very high standards in terms of contiguity (chromosome level) and completeness. Overall, the manuscript is well explained and data generated will be very useful, not only for researchers interested in this group of molluscs, but any interested in venomics.However, I have a couple of issues that in my opinion should be addressed before the acceptance of the manuscript.First of all, I would strongly recommend to include some kind of contamination analysis (e.g. blobtools https://github.com/DRL/blobtools). It is something fast and easy to run and could discard any contings-sccaffolds that come from other organisms. I don't think that data here presented showed any problem of contamination, but I think is an approach that should always be done to check the quality of raw data.My second major concern is related to annotation quality, that showed a 13% of missing BUSCOs. The high contiguity of the genome here provided, with very good completeness (only 2.7% of missing BUSCOs), makes me think that the annotation could be improved. This idea is also supported by the use of an old version of Augustus (v2.5.5.), because since many time ago the version 3.0 is available and it has showed to recover better predictions. In any case, I would recommend to authors to try another annotation pipelines such as GeMoMa, EVidenceModeller or BRAKER2 to try to recover a better annotation scheme.I also have attached some minor comments in the pdf version of the main manuscript and supplementary material that could help to improve the manuscript.

Level of Interest

Please indicate how interesting you found the manuscript: Choose an item.

Quality of Written English

Please indicate the quality of language in the manuscript: Choose an item.

Declaration of Competing Interests

Please complete a declaration of competing interests, considering the following questions:

- Have you in the past five years received reimbursements, fees, funding, or salary from an organisation that may in any way gain or lose financially from the publication of this manuscript, either now or in the future?
- Do you hold any stocks or shares in an organisation that may in any way gain or lose financially from the publication of this manuscript, either now or in the future?
- Do you hold or are you currently applying for any patents relating to the content of the manuscript?
- Have you received reimbursements, fees, funding, or salary from an organization that holds or has applied for patents relating to the content of the manuscript?
- Do you have any other financial competing interests?
- Do you have any non-financial competing interests in relation to this paper?

If you can answer no to all of the above, write 'I declare that I have no competing interests' below. If your reply is yes to any, please give details below.

I declare that I have no competing interests

I agree to the open peer review policy of the journal. I understand that my name will be included on my report to the authors and, if the manuscript is accepted for publication, my named report including any attachments I upload will be posted on the website along with the authors' responses. I agree for my report to be made available under an Open Access Creative Commons CC-BY license (http://creativecommons.org/licenses/by/4.0/). I understand that any comments which I do not wish to be included in my named report can be included as confidential comments to the editors, which will not be published.

Choose an item.

To further support our reviewers, we have joined with Publons, where you can gain additional credit to further highlight your hard work (see: https://publons.com/journal/530/gigascience). On publication of this paper, your review will be automatically added to Publons, you can then choose whether or not to claim your Publons credit. I understand this statement.

Yes Choose an item.
